# Accurate and Efficient Resolution of Overlapping Isotopic Envelopes in Protein Tandem Mass Spectra

**DOI:** 10.1038/srep14755

**Published:** 2015-10-06

**Authors:** Kaijie Xiao, Fan Yu, Houqin Fang, Bingbing Xue, Yan Liu, Zhixin Tian

**Affiliations:** 1 Department of Chemistry and Shanghai Key Laboratory of Chemical Assessment and Sustainability, Tongji University, Shanghai 200092, China

## Abstract

It has long been an analytical challenge to accurately and efficiently resolve extremely dense overlapping isotopic envelopes (OIEs) in protein tandem mass spectra to confidently identify proteins. Here, we report a computationally efficient method, called OIE_CARE, to resolve OIEs by calculating the relative deviation between the ideal and observed experimental abundance. In the OIE_CARE method, the ideal experimental abundance of a particular overlapping isotopic peak (OIP) is first calculated for all the OIEs sharing this OIP. The relative deviation (RD) of the overall observed experimental abundance of this OIP relative to the summed ideal value is then calculated. The final individual abundance of the OIP for each OIE is the individual ideal experimental abundance multiplied by 1 + RD. Initial studies were performed using higher-energy collisional dissociation tandem mass spectra on myoglobin (with direct infusion) and the intact *E. coli* proteome (with liquid chromatographic separation). Comprehensive data at the protein and proteome levels, high confidence and good reproducibility were achieved. The resolving method reported here can, in principle, be extended to resolve any envelope-type overlapping data for which the corresponding theoretical reference values are available.

Protein characterization is performed with several commercially successful techniques, such as soft ionization (e.g., electrospray ionization and ESI^1^), high-resolution affordable mass analyzers (e.g., Orbitrap^2^), efficient gas-phase dissociation (e.g., collision-induced dissociation (CID), higher-energy collisional dissociation (HCD)^3^ and electron-transfer dissociation (ETD)^4^), and state-of-the-art bioinformatics tools^5^,^6^,^7^,^8^,^9^,^10^,^11^,^12^,^13^,^14^,^15^,^16^,^17^,^18^,^19^,^20^,^21^,^22^,^23^,^24^,^25^,^26^,^27^. Tandem mass spectrometry has also become a state-of-the-art platform for the characterization of high-throughput peptides and proteins. The interpretation of tandem mass spectra is the final, yet paramount, step of this platform. Overlapping isotopic envelopes (OIEs) are very common in these peptide and protein tandem mass spectra because many isotopic envelopes peak in a very narrow *m/z* range. In addition, it has long been a challenge to accurately and efficiently resolve these OIEs to maximize matching product ions and identify proteins^28^,^29^,^30^,^31^,^32^.

Few resolving algorithms using Averagine and its variant models have been reported. THRASH resolves OIEs through sequential subtraction of best-fit abundance distributions; in the FTMS spectrum of GluC digest of a 191 kDa protein, THRASH found 65 more isotopic clusters (out of 824)^33^. In 2006, L. Chen *et al.* reported the AID-MS algorithm and a Lorentzian-based peak-subtraction technique to resolve OIEs in high-peak-density regions^34^. In the analysis of the plasma electron-capture dissociation (ECD) spectra of ubiquitin and carbonic anhydrase, AID-MS found 509 and 611 isotopic envelopes, respectively. In 2008, K. Park *et al.* reported an isotopic peak-intensity ratio based on an algorithm for which all possible pseudo-envelopes were considered and OIEs could be identified^30^. MS-Deconv efficiently finds optimal set of envelopes through explicitly scoring combinations of candidate envelopes^35^.

It is worth noting that quite a few algorithms have been reported to interpret OIEs of peptide precursor ions in the MS spectra. The observed overlap here is due to the small mass differences from either the peptides themselves^36^,^37^, post-translational modifications (PTMs, citrullination^38^, deamidation and ^18^O-labeling^39^, metal-binding and sulfhydryl reduction^40^), or quantitative chemical labeling (dimethyl^41^, acrylamide^42^, and mTRAQ^43^).

Here, we report an alternative resolving method for OIEs based on exact isotopic envelopes computed from the elemental composition of the product ions’ actual amino acids. This method is in contrast to the strategies existing in published work that are used to determine the theoretical isotopic envelopes, which are composed of Averagine units. In this method, the ideal experimental abundance of a particular overlapping isotopic peak (OIP) is first calculated for all the OIEs sharing this OIP. An OIP is an experimental isotopic peak with its m/z values matched by theoretical isotopic peak m/z values from two or more product ions within the *m/z* tolerance. The relative deviation (RD) of the observed experimental abundance of this OIP relative to the summed ideal value is then calculated. The final individual abundance of the OIP in each OIE is defined as its ideal experimental abundance multiplied by 1 + RD. Currently, this method has been implemented in our automated intact protein database search engine ProteinGoggle^44^ with user-friendly graphical user interfaces. ProteinGoggle, which runs on personal computers with Windows operating systems, is currently freely available at http://proteingoggle.tongji.edu.cn/. Tandem mass spectra from both individual proteins (with direct infusion) and proteome mixtures (with liquid chromatography separation) can be interpreted automatically. Initial results from the HCD spectra of myoglobin and *E. coli* are presented in this manuscript. The method reported here is expected to perform equally well with the tandem mass spectra of small peptides and better with the tandem mass spectra of large proteins in comparison with the current methods reported in the literature.

## Results

### HCD of Myoglobin

Three technical replicate HCD spectra of myoglobin, with abundances of 1.65E6, 1.61E6 and 1.67E6, were acquired and searched using ProteinGoggle with a search tolerance of IPACO = 20%, IPMD = 15 ppm and IPAD = 50%. IPACO, IPMD and IPAD are acronyms for “isotopic peak abundance cutoff” (in %), “isotopic peak *m/z* deviation” (in ppm) and “isotopic peak-abundance deviation” (in %), respectively.

The subsequent matching b and y ions, sequence coverage, peptide bond coverage, interpreted isotopic peaks, and interpreted abundance from the forward search of the three replicate spectra were listed in Table 1. The peptide bond coverage is defined as the percentage of peptide bonds that have at least one matching b or y ion. The sequence coverage is defined as the percentage of amino acids in the protein sequence that is covered by matching b and y ions. For a protein has n amino acids and the biggest matching b and y ions are b_j_ and y_k_, respectively, if j + k ≥ n, the sequence coverage is 100%; if j + k < n, then the sequence coverage equals to (j + k)*100%/n. An average of 92 matching b or y ions was found after a full 100% sequence coverage. The average peptide bond coverage was 42.5%. Both experimental isotopic peaks and abundances were comprehensively interpreted: the percentages were 95.6 ± 0.1 and 99.2 ± 0.0, respectively.

**Figure 1:**
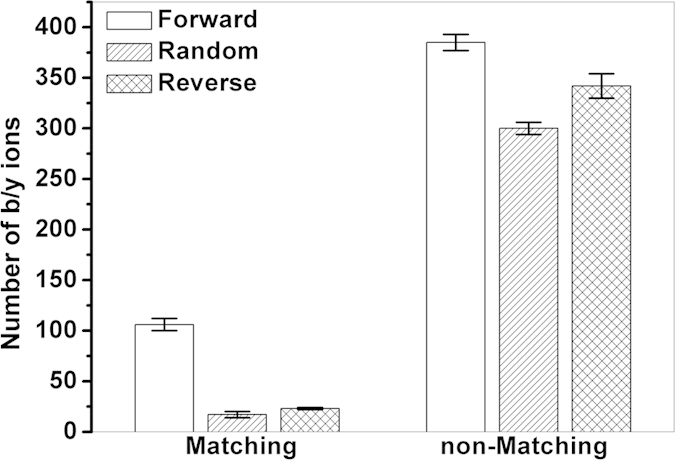
Matching vs. non-matching b/y ions from the forward, random and reverse database search of the HCD spectra of myoglobin. The error bars are the result of three technical replicates.

Besides forward search described above, random and reverse searches were also carried out for the three HCD of myoglobin. The average numbers of matching and non-matching b and y ions with standard deviation from the random and reverse searches vs. those from the forward search were plotted in Fig. 1; the detailed lists of these matching and non-matching b and y ions are provided in supplemental Tables S1 and S2, respectively.

### RPLC-MS/MS of the *E. coli* Intact Proteome

Three technical replicates of reversed-phase liquid chromatography (RPLC)-MS/MS datasets from *E. coli* were acquired. The total HCD spectra were 16573, 16533, and 16591. The abundance of the MS-only base-peak chromatograms were 2.62E8, 2.92E8, and 2.77E8, respectively (Supplemental Figure S1). ProteinGoggle was used to search the datasets. Two distinct tolerance parameters for MS and MS/MS spectra, as described in the following Methods section, were used for this analysis.

The resultant protein spectrum matches (PrSMs), unique proteoforms together with their averaged sequence coverage, peptide bond coverage, interpreted isotopic peaks, and interpreted abundance from each dataset were summarized in Table 2. With a spectrum-level false discovery rate (FDR) of 1%, an average of 5105 ± 544 PrSMs with PMPs ≥ 5 were identified from the three datasets. PMPs, the percentage of matching product ions, is defined as the minimum percentage of the experimental matching product ions for the identification of a PrSM. “Amino acid sequence” and the corresponding “PTMs” were used as criteria to group PrSMs from each dataset in Microsoft Excel to remove duplicates and obtain unique proteoforms. A proteofrom may be identified multiple times from the same precursor ion in different TopN cycles or different precursor ions of different charge states; only the PrSM with the most matching b and y ions are kept for the final protein ID (i.e., proteoform). With grouping, an average of 105 ± 2 unique proteoforms were identified from the three datasets. The detailed information (including retention time, protein ID, sequence length, PTMs, PTM Score, -log(P Score), sequence coverage, peptide bond coverage, interpreted isotopic peaks, and interpreted abundance) for each unique proteoform in the three datasets was provided in Supplemental Tables S3, S4, and S5, respectively. PTM Score is defined as the total number of non-redundant matching product ions containing the PTM that independently define the unique localization of a PTM; a product ion with multiple charge states are only counted once. The proteoforms have an average sequence coverage of 74.6 ± 1.8 and a peptide bond coverage of 26.7 ± 0.7. A higher coverage of sequence peptide bonds led to more confident protein identification and a greater chance of unique localization of PTMs.

**Figure 2:**
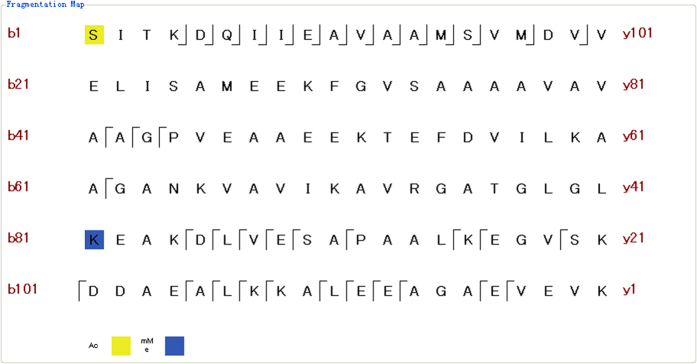
Graphical fragmentation map of the identified proteoform RL7_ECOLI (P0A7K2) with S1 acetylation and K81 methylation.

Among the 128 unique proteoforms that were identified from 3 technical replicates, 13 were modified with acetylation, biotinylation, monomethylation, trimethylation, O-(pantetheine 4′-phosphoryl), or a combination thereof. PTMs on 12 of these modified proteoforms were uniquely localized with high PTM scores. For example, in protein RL7_ECOLI (accession number P0A7K2), S1 acetylation and K81 methylation were identified with PTM scores of 25 and 4, respectively. These scores imply that 25 and 4 matching b or y ions independently defined the unique locations of these two modifications (Fig. 2).

## Discussion

The aforementioned matching product ions (short for MPs, including both b and y ions) have ideal experimental isotopic envelopes. An experimental isotopic envelope is considered an ideal isotopic envelope if all of its experimental isotopic peaks (above IPACO) are observed and their *m/z* and relative abundance are within the tolerance of IPMD and IPAD, respectively. On the other hand, an experimental isotopic envelope is a non-ideal isotopic envelope if any of its experimental isotopic peaks (above IPACO) are not observed or if the relative abundance of any observed experimental isotopic peak is larger than IPAD. The product ions with non-ideal isotopic envelopes are defined as non-matching product ions (short for non-MPs) accordingly.

The presence of MPs vs. non-MPs was evaluated using two search methods: first, by performing random and reverse searches for the three myoglobin HCD spectra, and second, by a forward database search (Fig. 1). In both search methods, the non-MPs exhibited much higher randomness than MPs. The number of non-MPs in both random and reverse searches is on the same order of magnitude as that of the forward search. It is, therefore, paramount to use only MPs for protein identification, as well as for PTM localization. Using the ProteinGoggle database, in which all theoretical ions are pre-stored, the search for a tandem mass spectrum leads to a match with a product ion only if its theoretically highest (i.e., 100%) isotopic peak is observed with an *m/z* deviation smaller than or equal to the IPMD tolerance. Whether this ion is matching or non-matching is further categorized using the IPACO and IPAD tolerances. Due to the nature of both the reverse and random databases, a greater number of matching and non-matching products ions are usually found in the corresponding forward search.

A benefit of using the OIE_CARE method to resolve OIEs is that the appropriate individual experimental abundance of the shared OIPs is retrieved. Therefore, certain otherwise non-MPs are turned into MPs. More MPs were generally found for both the myoglobin and *E. coli* proteoforms. Four more proteins were identified from the *E. coli* datasets.

**Figure 3:**
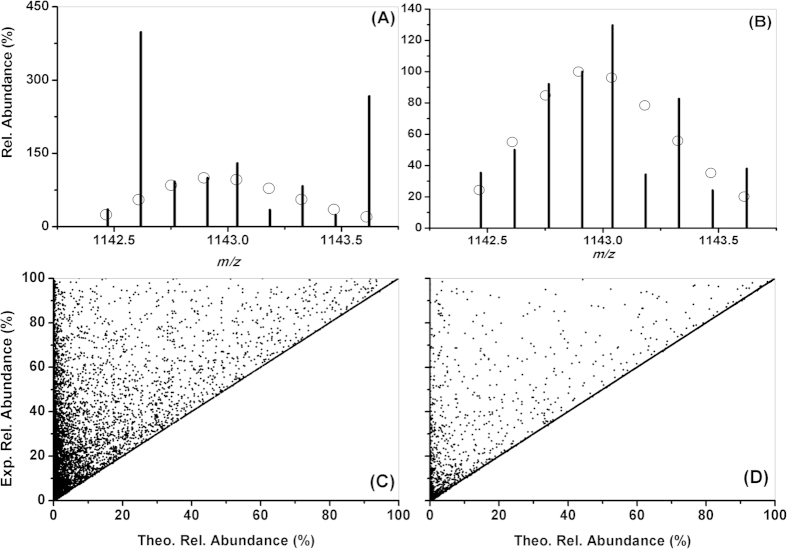
The iEF maps of y72-7+ and orthogonal plots of experimental vs. theoretical relative abundance of all interpreted isotopic peaks (with IPAD ≥ 0) without (A,C) and with (B,D) OIE_CARE resolving of OIEs for one of the HCD spectra of myoglobin. The bars and circles in (**A**,**B**) are the experimental and theoretical data, respectively. Rel. = relative, Exp. = experimental, and theo. = theoretical.

For HCD of myoglobin, IPADs of OIP *m/z* 1142.617676 in y10-1+ and y72-7+ were reduced from 214 to –2 and 343 to –5, respectively (Table 3). Thus, these two non-MPs were converted into MPs. A total of 141 unique matching b or y ions were found from the three replicate spectra. The number reduced to 134 when the OIE_CARE method was disabled. This implies that 7 more matching b/y ions (b76-7+, y10-1+, y136-13+, y149-15+, y58-6+, y72-7+, y76-7+) were found by resolving OIPs using OIE_CARE. As an example, the iEF maps of y72-7+ without and with using OIE_CARE are shown in Fig. 3(A,B), respectively. The theoretical *m/z*, theoretical relative abundance, experimental *m/z*, experimental relative abundance before and after the resolution for each isotopic peak in Fig. 3 are provided in Table S6. It is worth noting that resolving the experimental abundance of OIPs in a tandem mass spectrum increases the number of matching ions (and decreases number of non-matching ones) in general. At the same time, the total number of ions (including both matching and non-matching) remains the same. When a, b, and y ions and their neutral loss (NL) ions were included in the database search, 84.2 ± 0.3% of the interpreted isotopic peaks in myoglobin HCD spectra were found to be OIPs. The overlapping percentage of matching b and y ions was 96.8 ± 0.9%. When a stringent IPMD of 5 ppm was used in the search, the percentages of matching OIPs and overlapping b and y ions were 43.7 ± 2.3% and 60.8 ± 5.3%, respectively. Therefore, the efficient and accurate resolving of such a high percentage of OIPs and OIEs is indispensable to confidently maximizing the matching product ions and protein identification. The OIE_CARE and partition of overlapping abundance of OIPs were used to resolve the experimental relative abundance of all interpreted isotopic peaks with IPAD ≥ 0 from all matching and non-matching ions. The result was that these were also comprehensively brought very close to their corresponding theoretical values. Comparative results from one of the myoglobin HCD spectra with or without using OIE_CARE are presented in Fig. 3(C,D); where the experimental relative abundance of all interpreted isotopic peaks (in all matching and non-matching product ions) are plotted against the corresponding theoretical relative abundance. These abundance together with m/z values are default output of ProteinGoggle for both matching and non-matching product ions. It should be noted that isotopic peaks with IPAD > 0 are, in general, OIPs with a shared experimental abundance. Equivalent plots of the interpreted isotopic peaks with IPAD < 0 show no observed essential changes and are provided in Supplemental Figure S2. As seen from Table 3, to resolve an OIP with n OIEs (or product ions), only 2n + 1 simple arithmetic (addition, subtraction, multiplication, or division) calculations are necessary. This linear computation load relationship with the size of the OIEs is especially advantageous for OIPs with many OIEs. For the HCD spectra of myoglobin, the isotopic peak of *m/z* is 1123.608521 and is shared by 26 product ions (b111-2H_2_O-11+, b111-H_2_O-NH_3_-11+, b111-2NH_3_-11+, y142-2H_2_O-14+, y142-H_2_O-NH_3_-14+, b142-2H_2_O-14+, y142-2NH_3_-14+, b142-H_2_O-NH_3_-14+, a111-11+, b142-2NH_3_-14+, a92-2H_2_O-9+, b70-7+, a152-H_2_O-15+, y10-H_2_O-1+, y71-7+, b152-2H_2_O-15+, b152-H_2_O-NH_3_-15+, b152-2NH_3_-15+, y142-H_2_O-14+, b121-12+, y142-NH_3_-14+, a71-2H_2_O-7+, y103-2H_2_O-10+, a152-15+, and y103-2NH_3_-10+).

**Figure 4:**
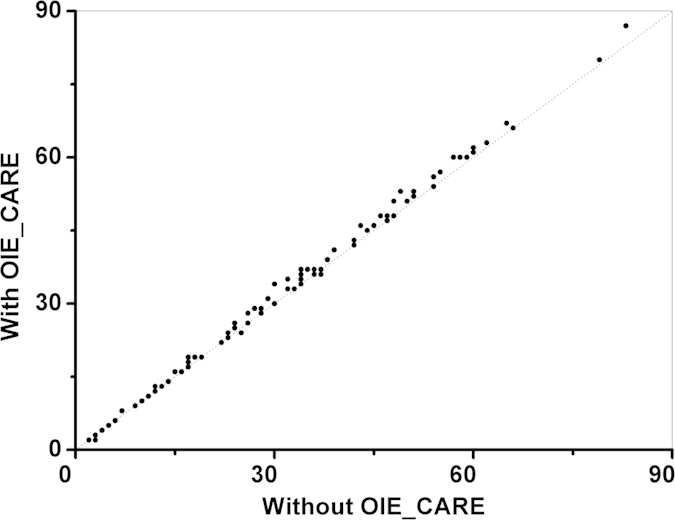
The orthogonal plot of matching b and y ions with vs. without using the OIE_CARE method for the unique *E. coli* proteoforms. The orthogonal dotted line is added as a visual guide.

For *E. coli* protein identification at the proteome level, more matching b and y ions were found with the OIE_CARE resolving OIEs for most of the identified proteoforms (Fig. 4). For example, 3, 6, and 3 more matching b and y ions were found for GRCA_ECO45, IHFB_ECO24 and DBHB_ECO57, respectively. The corresponding labeled MS/MS spectra are provided in Supplemental Figure S3. With OIE_CARE and more matching b and y ions, four new proteins (ASR_ECOLU, C562_ECO57, YNFD_ECOLI, and RNFH_ECO7I) were also identified. The graphical fragmentation maps, along with matching b and y ions of these four new proteins, are provided in Supplemental Figure S4.

**Figure 5:**
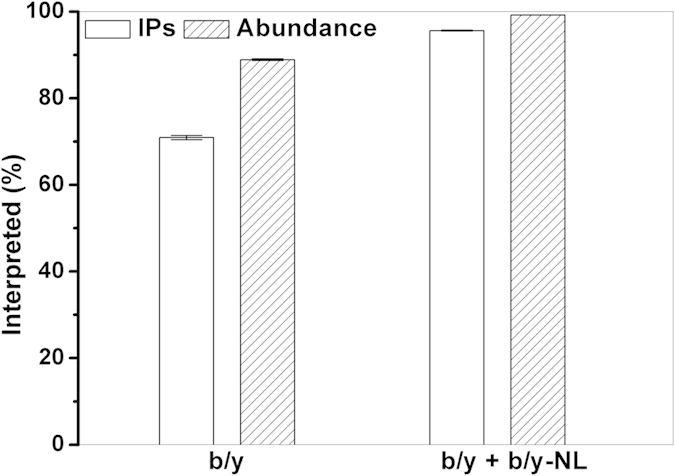
Interpreted isotopic peaks and abundance of the myoglobin HCD spectra when two incremental combinatorial ions series, b/y and b/y + b/y-NL (including a and a-NL), are searched separately. The error bars arise from three technical replicates. IPs = Isotopic Peaks.

Protein-level comprehensiveness, in terms of the percentage of interpreted experimental isotopic peaks and abundance, has been achieved when b, y and their NL ions (including “a” and “a-NL” ions) are included in the database search (Table 1). To evaluate the individual contribution of the various ion series (only b and y ions), the HCD spectra of myoglobin were also independently searched using the same set of tolerance parameters as described above. An increase in the interpreted isotopic peaks and abundance versus these two combinatorial ion series are shown in Fig. 5. The b or y ions are approximately 70% in the number of isotopic peaks and approximately 90% in abundance. This implies that the b or y ions are the most abundant ion series in the HCD spectra of myoglobin. The b or y-NL (including a and a-NL) are approximately 26% in the number of isotopic peaks but approximately 10% in the total abundance. The remaining less than 4% of the isotopic peaks belonged to internal ions or their NL ions. Their total abundance (<1%) is negligible in this case. For comprehensiveness at the proteome level, the identification rate of the *E. coli* tandem mass spectra from the three technical replicate RPLC-MSMS runs is 73.3 ± 3.4%. The identification rate is defined as the total number of PrSMs from the dataset divided by the total number of MS/MS spectra between the first and last PrSMs. Here the MS/MS spectra were acquired only for precursors with ≥5 or with unassigned charge states. This rate could be further improved by additional search of the proteolytic peptidome, as well as by more comprehensive annotation of PTMs. The current protein-annotation rate in terms of ‘MOD_RES’ in the flat text file was only 5.2%. These extra utilities for ProteinGoggle are under development.

**Figure 6:**
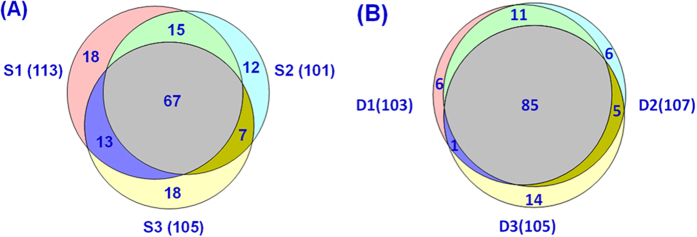
Venn diagrams of the matching b/y ions of the three technical replicate HCD spectra (S1, S2, and S3) of myoglobin (A) and identified unique proteoforms of the three technical replicate RPLC-MSMS datasets (D1, D2, and D3) of the *E. coli* intact proteome (B).

The protein-level reproducibility was characterized with matching b and y ions, which are the two ion series used for protein identification scoring and PTM localization. The results are shown in Fig. 6(A). The shared matching b and y ions among all three replicates are more than 60%. The proteome-level reproducibility was characterized using identified unique proteoforms, and those shared among the three technical replicates of the RPLC-MS/MS analysis of the *E. coli* intact proteome were more than 80% (Fig. 6(B)). Better reproducibility at the proteome level would be possible with additional dimension(s) of separation to increase the dynamic detection range.

Overall, OIEs are very common in protein tandem mass spectra, and the efficient resolving of these OIEs is essential for maximizing the matching product ions, improving the confidence in protein identification, and achieving unique localization of PTMs. Using theoretical isotopic envelopes as a reference, the OIE_CARE method, as implemented in ProteinGoggle, efficiently disentangles OIEs at the raw experimental data level. This not only produces good orthogonality between the experimental and theoretical data, but it also maximizes the matching product ions and confidence in the protein identification. This computationally efficient method could, in principle, be extended to resolve any envelope-type overlapping data for which the corresponding theoretical reference values are available.

## Methods

### Reagents

Myoglobin (from horse heart, M1882), acetonitrile (CHROMASOLV gradient grade, 34851) and formic acid (FA, eluent additive for LC-MS, 56302) were purchased from Sigma-Aldrich (St. Louis, MO, USA). Tryptone (TG217), yeast extract (G0961), NaCl (F20051212), PBS (SB0627), PMSF (PB0425), and a BCA Reagent Kit (SK3021) were bought from Sangon Biotech (Shanghai, China). Ultrapure water was produced in the laboratory using the Millipore Simplicity system.

### Cell Culture of *E. coli* and Protein Extraction

A conical flask with 2 g tryptone, 1 g yeast extract, 2 g NaCl and 200 mL doubly distilled H_2_O was covered with aluminum foil and sterilized in a Shen’an high-pressure steam sterilizer (LDZX-50FBS, Shanghai, China) at 121 °C for 21 min. After cleaning the outer wall with 75% alcohol, the flask was transferred into a Suzhou Antai ultraclean bench (SW-CJ-2FD, Suzhou, Jiangsu, China) and pre-disinfected with UV for 15 min while cooling down to room temperature. A fresh *E. coli* colony was then injected into the flask and cultured overnight at 37 °C and 220 rpm in a ZHICHENG shaker (ZWY-240, Shanghai, China). After centrifugation for 5 min at 8000 rpm and 4 °C (Eppendorf, Centrifuge 5804R, Hamburg, Germany), the cell pellet was washed three times with 20 mL PBS. The pellet was then re-suspended in 5 mL PBS with 50 μL PMSF. Cells were lysed in a 1.5 mL centrifuge tube over ice using a Ningbo Scientz (Ningbo, Zhejiang, China) ultrasonic cell disruptor. Each cycle consisted of running the sample for 5 s (at 300 J, 4 °C) and pausing for 10 s. This cycle was continuously run for 5 min. After centrifugation for 15 min at 10000 rpm and 4 °C (Eppendorf, Centrifuge 5804R, Hamburg, Germany), the protein concentration in the supernatant was measured using a BCA assay in TECAN Infinite F50 (Salzburg, Austria) according to the manufacturer’s protocol. The *E. coli* proteome solution was finally aliquoted into 1.5-mL centrifuge tubes and stored in a refrigerator (at −80 °C) for future use.

### HCD of Myoglobin

HCD tandem mass spectra of myoglobin in the profile mode were acquired using a Thermo Scientific Q Exactive Orbitrap mass spectrometer (Waltham, MA, USA). The myoglobin solution (2 μM, CH_3_OH/H_2_O 3:1 (v/v), HCOOH 1%) was electrosprayed, and a 15+ ion (*m/z* 1131), was isolated with an isolation width of 6.0 *m/z* and fragmented at an NCE of 24%. An AGC (automatic gain control) target of 5E5 was used and three technical replicate spectra (S1, S2, and S3) were acquired at a 70 K resolution using 10 microscans.

### RPLC-MS/MS of *E. coli* Proteome

RPLC tandem mass spectrometry using HCD of the *E. coli* intact proteome was performed using a Thermo Scientific Q Exactive mass spectrometer coupled with a Dionex UltiMate 3000 RSLCnano high-performance liquid chromatography (HPLC) system. The analytical column (75 μm i.d., 60 cm long) was packed in-house with C4 (5 μm, 300 Å). The trap column was packed with the same particles, but with an i.d. of 200 μm and a length of 5 cm. Buffer A consisted of 5% ACN, 94.8% H_2_O and 0.2% FA. Buffer B consisted of 95% ACN, 4.8% H_2_O and 0.2% FA. After being trapped on the column, the *E. coli* proteome was eluted using the following linear gradient: 0 min, 1% B; 1 min, 15% B; 92 min, 65% B; 98 min, 75% B; and 103 min, 99% B. At 99% B, the system was held for an additional 15 min. The MS spectra of the precursor ions were acquired with the following settings: microscans, 2; resolution, 70,000 (*m/z* 200); AGC, 3E6; and scan range, *m/z* 600–2,000. The data-dependent Top10 tandem HCD spectra acquisition settings were as follows: microscans, 1; resolution, 35,000 (*m/z* 200); AGC, 5E5; maximum IT, 250 ms; isolation window, 10 *m/z*; NCE, 30%; charge exclusion, 1–4; and dynamic exclusion, 20.0 s. Both MS and MS/MS spectra were acquired in the centroid mode. Overall, three technical replicate RPLC-MS/MS datasets (D1, D2, and D3) were acquired.

### Database Search Using ProteinGoggle

The intact protein database search using ProteinGoggle, implemented with the isotopic mass-to-charge (*m/z*) ratio and envelope fingerprinting (iMEF) search algorithm, has been fully reported elsewhere, and only a brief description is given here. Theoretical precursor isotopic envelope databases were created for all possible charge states of every proteoform in the MS acquisition window. The theoretical product-ion isotopic envelope databases were created with ion series of a/b/y and a/b/y-NL (NL = NH_3_, H_2_O, NH_3_ + H_2_O, 2NH_3_, and 2H_2_O). H_2_O loss was a result of product ions containing the amino acids D/E/S/T. NH_3_ loss was a result of product ions containing the amino acids K/N/Q/R. For the above data-dependent spectra, both the precursor ions and product ions were “fished” from the theoretical isotopic envelope database and fully confirmed using isotopic *m/z* fingerprinting (iMF) and isotopic envelope fingerprinting (iEF), respectively. Two sets of values (40/15/100 and 20/15/50) were used in this study for the precursor and product ion search, respectively. These search parameters were pre-optimized at the proteome level for most protein IDs, with orthogonal combinatorial parameter design and FDR control (data not shown). In addition to the above search parameters, a value of PMPs ≥ 5 was used for the identification and output of PrSMs. Final protein identification with an FDR of 1% at the spectrum level was achieved through a decoy search using a random database and a P Score cutoff.

Flat text protein databases were downloaded from UniProt (www.uniprot.org). For myoglobin (145 AAs with initial methionine), the entry name is MYG_HORSE with an accession number of P68082. For the *E. coli* proteome, the text database includes 7,658 proteins (2589 unique proteins by the amino acid sequence). This was downloaded with the following criteria: ‘Organism [OS]’ = escherichia coli, ‘Sequence_Fragment’ = No, ‘Sequence_Sequence length’ = 1–200, and ‘Reviewed’ = Yes. The corresponding customized ProteinGoggle database was created using shotgun imagery. With all annotated PTMs (listed in Supplemental Table S7) treated dynamically, a total of 2,883 proteoforms for *E. coli* were created; i.e., 294 of these proteoforms have one or more PTM(s). For example, RL10_ECO7I has annotated acetylation (ac) on K37 and K105, respectively, and a total of 4 individual proteoforms (no PTM, K37ac, K105ac, and K37acK105ac) were created.

The resolving of OIPs using the OIE_CARE method proceeds according to the following three steps. Given an OIP shared by n OIEs, the ideal experimental abundance of this OIP for the ith ion (DEA_i_) is first calculated using Equation 1, where TA_i_ is the theoretically relative abundance of the OIP in this ion; EA_r_ and TA_r_ are the experimental absolute abundance and theoretical relative abundance of the reference isotopic peak of this ion, respectively. The reference isotopic peak is the normalization isotopic peak used to transform the absolute experimental abundance of all isotopic peaks of this ion into the relative experimental abundance. Second, the RD of the observed experimental abundance of this OIP (EA_OIP_) relative to the corresponding total ideal value 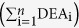 is then calculated (Equation 2). The final partitioned individual abundance of the OIP in the ith ion is its ideal value multiplied by the sum of one plus the relative deviation (Equation 3).


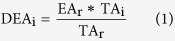



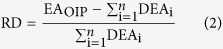



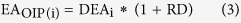


An example of resolving the OIP of *m/z* 1142.617676 shared by y10-1+, y20-2+ and y72-7+ using the OIE_CARE method is illustrated step-by-step in Table 3. According to Equation 1, the ideal experimental abundance for this particular OIP in y10-1+ (E3), y20-2+ (F3) and y72-7+ (G3) is firstly calculated using B7*D6/B6 and B10*D11/B11, and B14*D15/B15, respectively; e. g., the DEA of this OIP in y10-1+ was calculated as B7*D6/B6 = 23.01*128926.921875/63.64 = 46615.469396. This brings the total ideal value 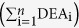 of this OIP to be 529680.016342 (E3 + F3 + G3). Given the EA_OIP_ = 480992.312500 (D3), the RD is computed to be −0.09 (H3) using Equation 2. The final individual experimental abundance for this OIP in y10-1+ (D7), y20-2+ (D10) and y72-7+ (D14) is calculated using E3*(1 + H3), F3*(1 + H3), G3*(1 + H3), respectively; e. g., the final experimental abundance of this OIP in y10-1+ is calculated to be 42330.617979 from 46615.469396*(1–0.09) using Equation 3. After resolving with OIE_CARE, the IPAD values of *m/z* 1142.617676 are changed from 214, 15, and 343 to –2, –9, and –5 in y10-1+, y20-2+ and y72-7+, respectively. This shows that the total abundance of this OIP is efficiently partitioned into the individual OIEs. In addition to the OIP of *m/z* 1142.617676, these three ions in combination with y144-14+ share another four OIPs (m/z 1140.619995, 1143.622437, 1144.620605, and 1145.624756). The experimental abundance of these OIPs can be resolved independently using the same three steps as described above. The full steps of the above five OIPs shared by the four product ions are provided in Supplemental Table S6.

The OIE_CARE resolving strategy and steps have been implemented in our intact protein database search engine ProteinGoggle. The database can be searched for individual tandem mass spectra from standard proteins with direct infusion or datasets from a proteome mixture with liquid chromatographic separation. The computer code corresponding to this part of the functionality is provided in Supplemental Scheme S1.

## Additional Information

**How to cite this article**: Xiao, K. *et al.* Accurate and Efficient Resolution of Overlapping Isotopic Envelopes in Protein Tandem Mass Spectra. *Sci. Rep.* **5**, 14755; doi: 10.1038/srep14755 (2015).

## Supplementary Material

Supplementary Information

## Figures and Tables

**Table 1:** Database Search Results of Three Technical Replicate HCD Spectra (S1, S2 and S3) of Myoglobin.

	Matching b/yions	Sequencecoverage (%)	Peptide bondcoverage (%)	Interpretedisotopic peaks (%)	Interpretedabundance (%)
S1	99	100.0	47.4	95.6	99.2
S2	88	100.0	40.8	95.6	99.2
S3	88	100.0	39.5	95.6	99.2
	92 ± 6	100.0 ± 0.0	42.5 ± 4.2	95.6 ± 0.1	99.2 ± 0.0

**Table 2:** Database Search Results of Three Technical Replicate RPLC-MSMS Datasets (D1, D2 and D3) of the *E. coli* Intact Proteome with a Spectrum-Level FDR of 1%. PrSMs = Protein Spectrum Matches.

	PrSMs	Proteo-forms	Sequencecoverage(%)	Peptidebondcoverage (%)	Interpretedisotopic peaks(%)	Interpretedabundance(%)
D1	4478	103	76.7	26.0	61.1	71.5
D2	5445	107	73.4	26.5	62.9	73.4
D3	5392	105	73.7	27.4	61.7	71.5
	5105 ± 544	105 ± 2	74.6 ± 1.8	26.7 ± 0.7	61.9 ± 0.9	72.1 ± 1.1

**Table 3:** Resolving the OIPs among y10-1+, y20-2+ and y72-7+ in the HCD Spectrum of Myoglobin Using the OIE_CARE Method.*

	A	B	C	D	E	F	G	H
1					**y10-1+**	**y20-2+**	**y72-7+**	
2			Exp. *m/z*	Exp. abun. (observed)	Exp. abun. (ideal)	Exp. abun. (ideal)	Exp. abun. (ideal)	Deviation
3			1142.617676	480992.312500	46615.469396	416521.895787	66542.651159	−0.09
4	**y10-1+**							
5	Theo. *m/z*	Theo. rel. abun.	Exp. *m/z*	Exp. abun.	Exp. rel. abun.	IPMD	IPAD	IPAD#
6	1141.607714	63.64	1141.613770	128926.921875	63.64	5	0	
7	1142.610419	23.01	1142.617676	42330.617979	20.89	6	−2	214
8	**y20-2+**							
9	Theo. *m/z*	Theo. rel. abun.	Exp. *m/z*	Exp. abun.	Exp. rel. abun.	IPMD	IPAD	IPAD#
10	1142.611961	100.00	1142.617676	378235.583145	90.81	5	−9	15
11	1143.113357	67.65	1143.120117	281777.062500	67.65	6	0	
12	**y72-7+**							
13	Theo. *m/z*	Theo. rel. abun.	Exp. *m/z*	Exp. abun.	Exp. rel. abun.	IPMD	IPAD	IPAD#
14	1142.607764	55.06	1142.617676	60426.111376	50.00	9	−5	343
15	1142.894250	100.00	1142.910522	120854.796875	100.00	14	0	

For OIP of *m/z* 1142.617676:

**Step 1:** The ideal exp. abundance for y10-1+ (**E3**), y20-2+ (**F3**) and y72-7+ (**G3**) is calculated using B7*D6/**B6** and B10*D11/**B11**, and B14*D15/**B15**, respectively.

**Step 2:** The deviation (**H3**) between the observed and ideal values is calculated using (**D3** − (**E3** + **F3** + **G3**))/(**E3** + **F3** + **G3**).

**Step 3:** The final individual exp. abun. for y10-1+ (**D7**), y20-2+ (**D10**) and y72-7+ (**D14**) is calculated using E3*(1 + **H3**), F3*(1 + **H3**), G3*(1 + **H3**), respectively.

^*^Only OIP of *m/z* 1142.617676 and the isotopic peaks used for normalization are listed here. The resolving of the other four OIPs of these three product ions and y144-14+ was performed using the same method. The full iEF information is provided in Table S6.

^#^The equivalent IPAD values without OIE_CARE. Exp. = experimental, theo. = theoretical, and abun. = abundance.
