# Supplementary material for: Accurate and Efficient Resolution of Overlapping Isotopic Envelopes in Protein Tandem Mass Spectra
Source: Sci Rep. 2015 Oct 6;5:14755. doi: 10.1038/srep14755 (PMC4593959; doi:10.1038/srep14755)
Supplement: Supplementary Information [file srep14755-s1.pdf]

Supplemental Information (106 pages)

Accurate and Efficient Resolution of Overlapping Isotopic Envelopes in Protein Tandem Mass Spectra

Kaijie Xiao, Fan Yu, Houqin Fang, Bingbing Xue, Yan Liu, Zhixin Tian*

Department of Chemistry and Shanghai Key Lab of Chemical Assessment and Sustainability, Tongji University, Shanghai 200092, China

*Corresponding author, zhixintian@tongji.edu.cn.

Contents

Figure S1. MS-only base-peak chromatograms from the three technical replicate RPLC-MS/MS analyses of *E. coli* intact proteome. (Page 4)

Figure S2. The orthogonal plots of experimental vs. theoretical relative abundance of all interpreted isotopic peaks (with IPAD<0) without (A) and with (B) OIE_CARE resolving OIEs of the first HCD spectra of myoglobin. Rel.=Relative, Exp.=Experimental, and Theo.=Theoretical. (Page 5)

Figure S3. Labeled MS/MS spectra of *E. coli* proteins of GRCA_ECO45, IHFB_ECO24 and DBHB_ECO57. The product ions marked as read are unique ones from resolving OIEs using OIE_CARE. (Page 6-8)

Figure S4. The graphical fragmentation maps and matching b and y ions of the 4 new proteins idenfied with OIE_CARE. (A), ASR_ECOLU; (B), C562_ECO57; (C), YNFD_ECOLI; (D), RNFH_ECO7I). (Page 9)

Table S1. Matching b/y ions from the forward, random and reverse searches of the three technical replicate HCD spectra of myoglobin. (Page 10-13)

Table S2. Non-matching b/y ions from the forward, random and reverse searches of the three technical replicate HCD spectra of

Table S3. Unique proteoforms identified from the 1st RPLC-MS/MS analysis (Dataset 1) of *E. coli* intact proteome. (Page 14-28)

Table S4. Unique proteoforms identified from the 2nd RPLC-MS/MS analysis (Dataset 2) of *E. coli* intact proteome. (Page 29-32)

Table S5. Unique proteoforms identified from the 3rd RPLC-MS/MS analysis (Dataset 3) of *E. coli* intact proteome. (Page 33-37)

myoglobin. (Page 38-41)

Table S6. Resolving of continuously overlapping y10-1+, y20-2+, y72-7+ and y144-14+ using OIE_CARE. (Page 42-44)

Table S7. PTMs annotated in the customized ProteinGoggle databases for the search of *E. coli* proteoforms in this study. (Page 45-49)

Scheme S1. OIE_CARE source code as implemented in ProteinGoggle. (Page 50-106)

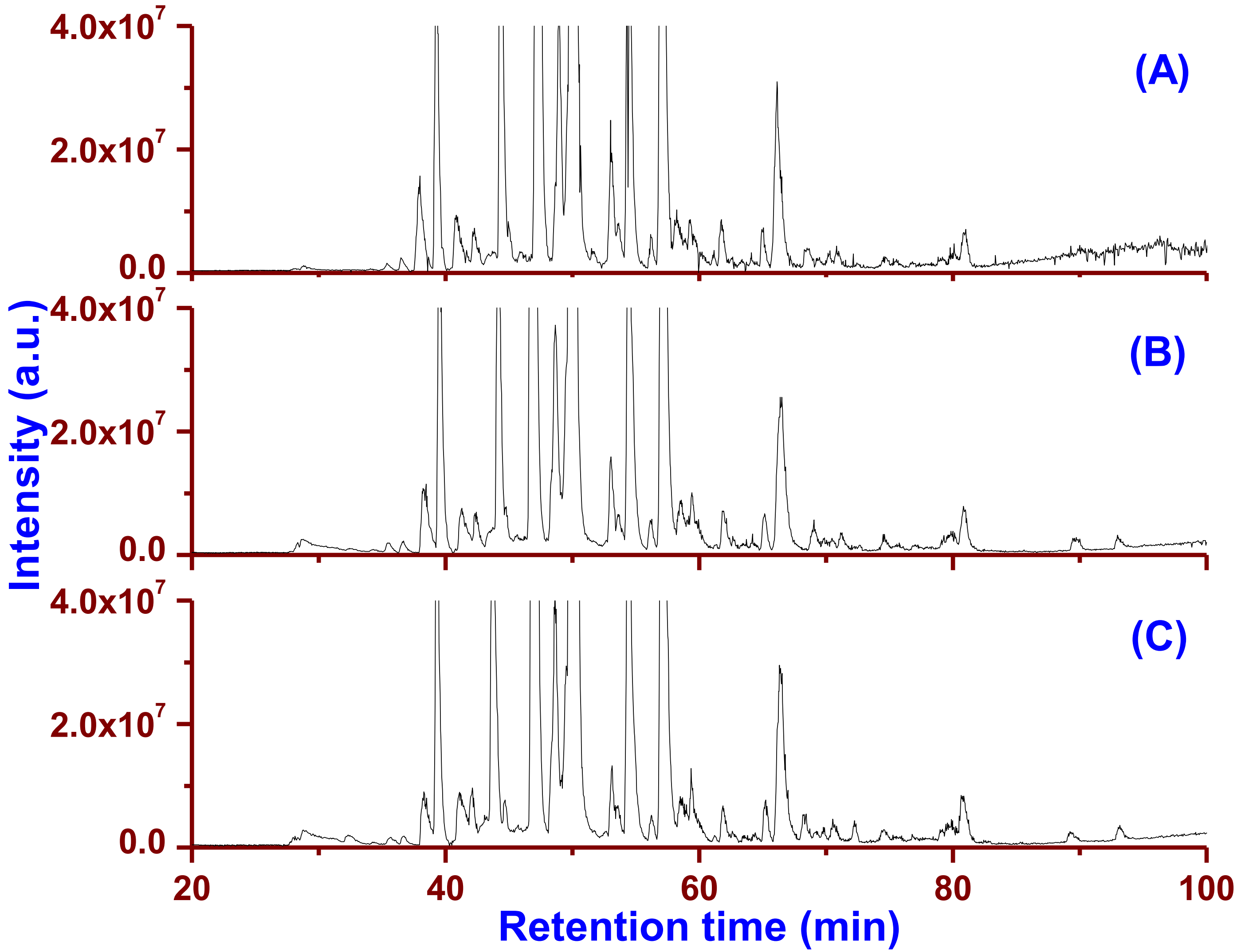

Figure S1. MS-only base-peak chromatograms from the three technical replicate RPLC-MS/MS analyses of *E. coli* intact proteome; a.u. = arbitrary unit.

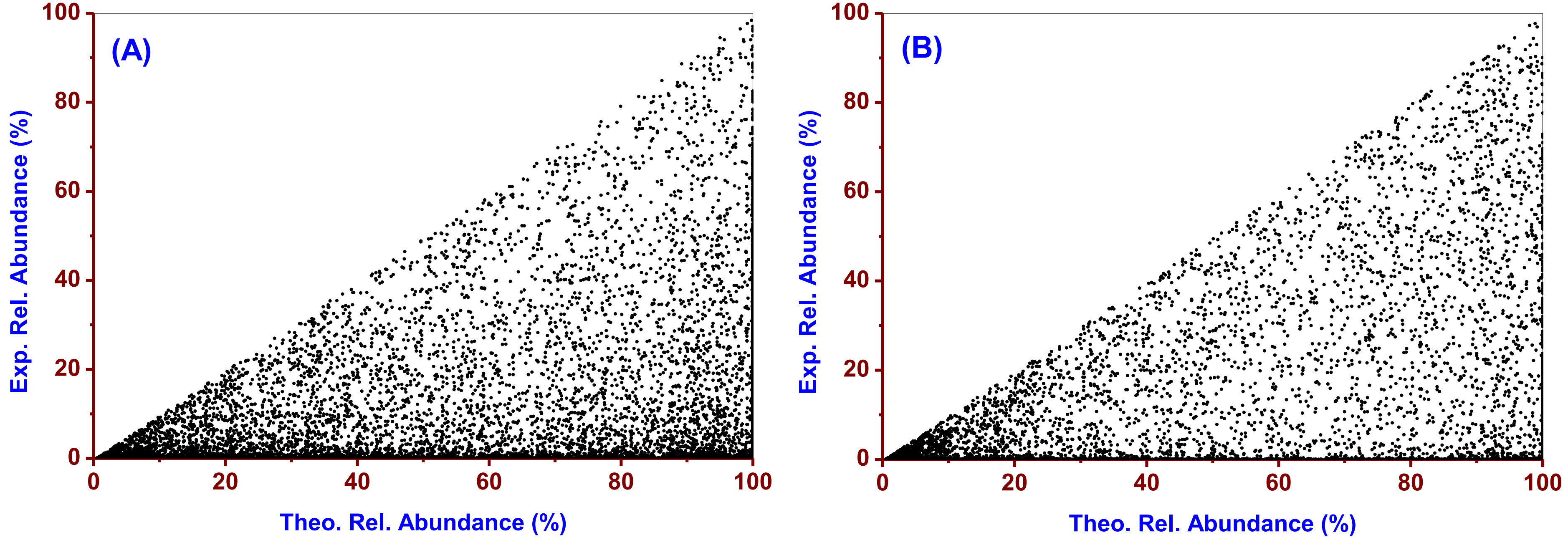

Figure S2. The orthogonal plots of experimental vs. theoretical relative abundance of all interpreted isotopic peaks (with IPAD<0) without (A) and with (B) OIE_CARE resolving of OIEs of one of the three HCD spectra of myoglobin. Rel. = Relative, Exp. = Experimental, and Theo. = Theoretical.

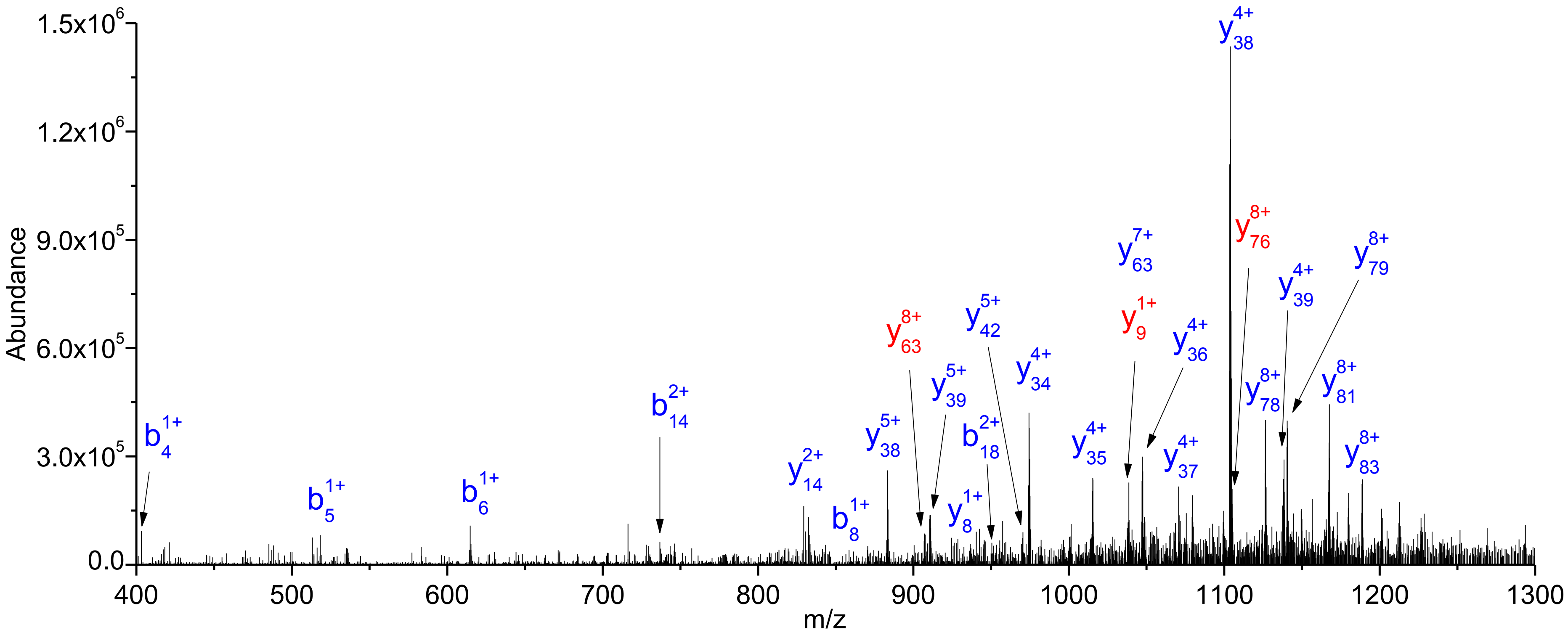
Abundance
m/z
1.5x10^6
1.2x10^6
9.0x10^5
6.0x10^5
3.0x10^5
0.0
400
500
600
700
800
900
1000
1100
1200
1300
b4 1+
b5 1+
b6 1+
b14 2+
y14 2+
b8 1+
y38 5+
y63 8+
y39 5+
y8 1+
b18 2+
y42 5+
y34 4+
y35 4+
y63 7+
y9 1+
y36 4+
y37 4+
y38 4+
y76 8+
y78 8+
y39 4+
y79 8+
y81 8+
y83 8+

(A)

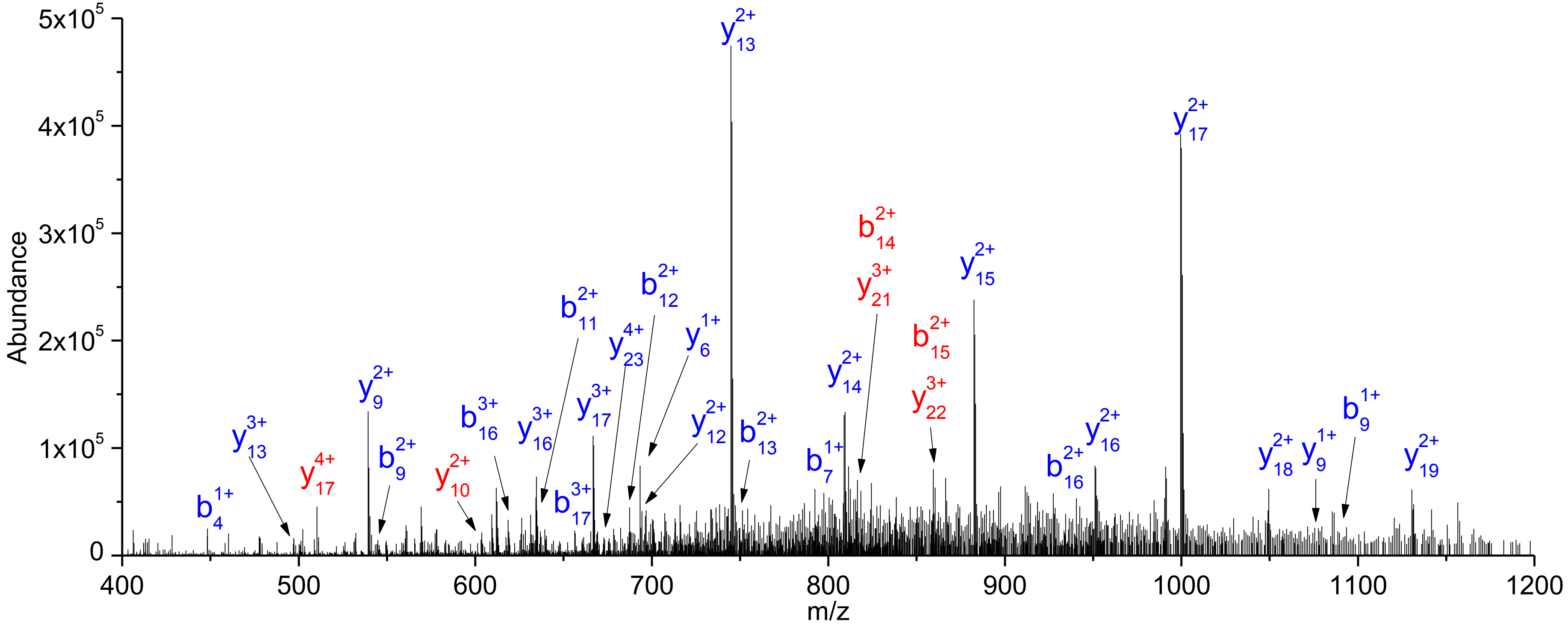
Abundance
m/z
0
1x10^5
2x10^5
3x10^5
4x10^5
5x10^5
400
500
600
700
800
900
1000
1100
1200
y13 2+
y17 2+
b14 2+
y21 3+
y15 2+
b15 2+
y22 3+
b11 2+
b12 2+
y23 4+
y6 1+
y14 2+
y9 2+
b16 3+
y16 3+
y17 3+
y12 2+
b13 2+
b7 1+
y13 3+
y17 4+
b9 2+
y10 2+
b17 3+
b4 1+
b16 2+
y16 2+
y18 2+
y9 1+
b9 1+
y19 2+

(B)

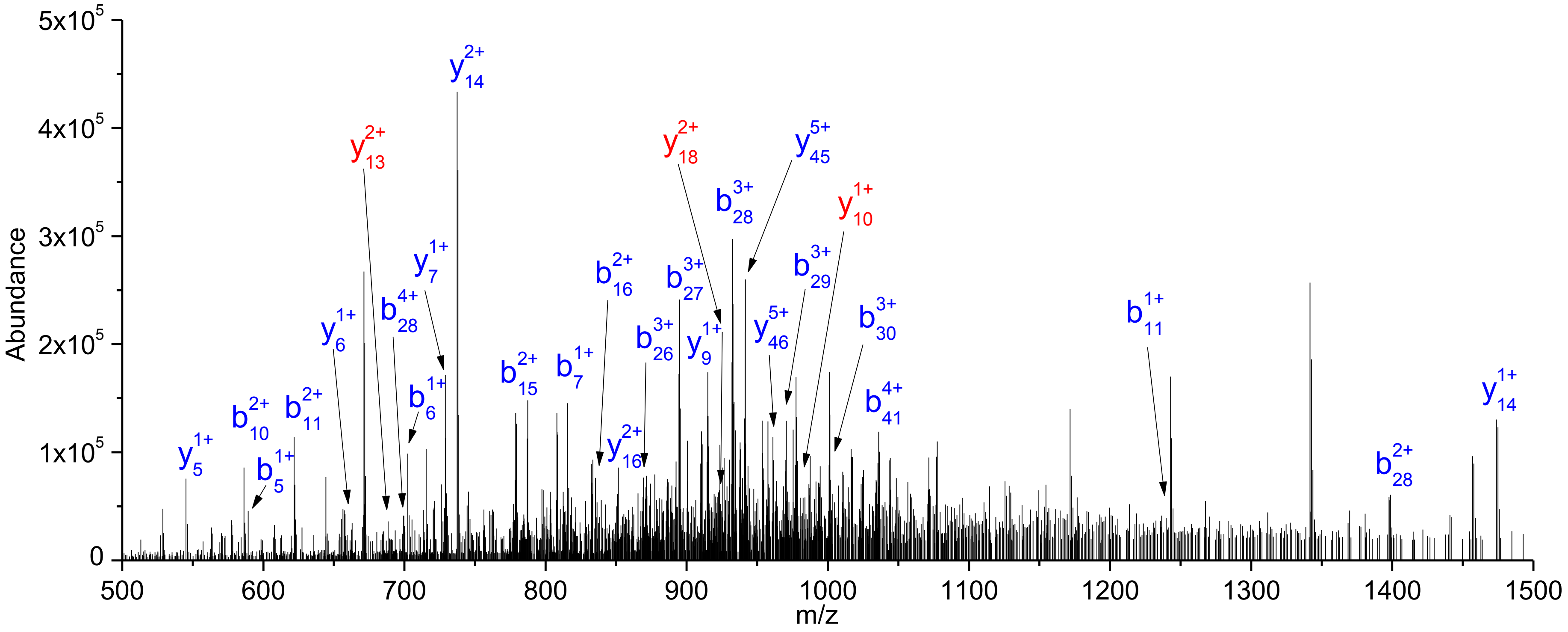

(C)

Figure S3. Labeled MS/MS spectra of *E. coli* proteins of GRCA_ECO45 (A), IHFB_ECO24 (B) and DBHB_ECO57 (C). The product ions marked as read are unique ones from resolving OIEs using OIE_CARE.

(A)

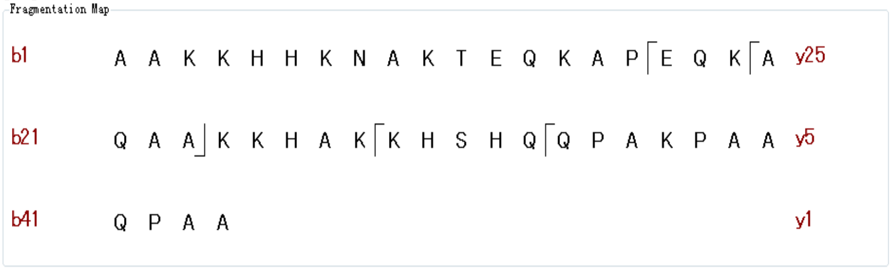

b23-3+O, y11-1+, y16-2+O, <u>y25-3+O</u>, y28-4+

(B)

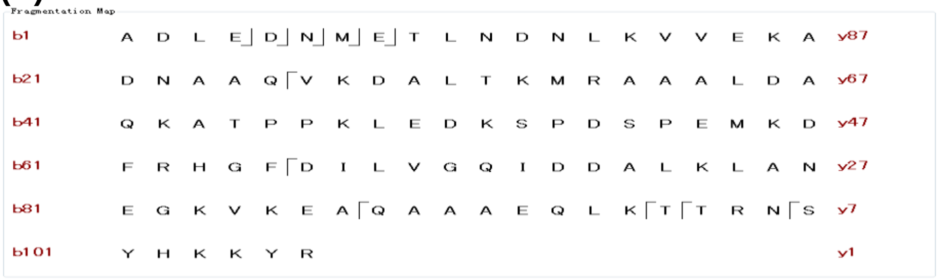

b4-1+, b5-1+, b6-1+, b7-1+, b8-1+, y7-1+, y10-1+ y11-1+, y19-2+, <u>y41-4+O</u>, <u>y81-8+O</u>

(C)

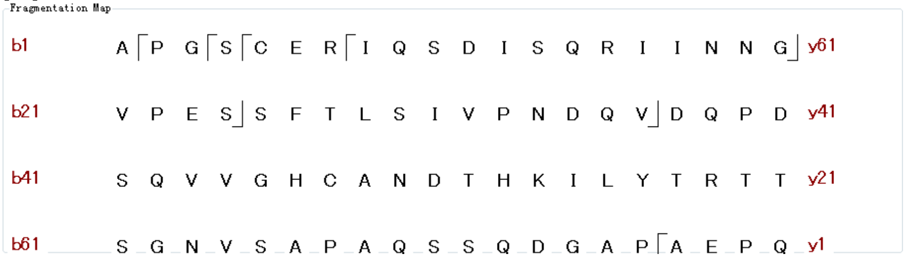

b20-2+, <u>b24-2+O</u>, b36-3+O, y4-1+, y73-6+O <u>y76-7+O</u>, y77-7+, y79-7+

(D)

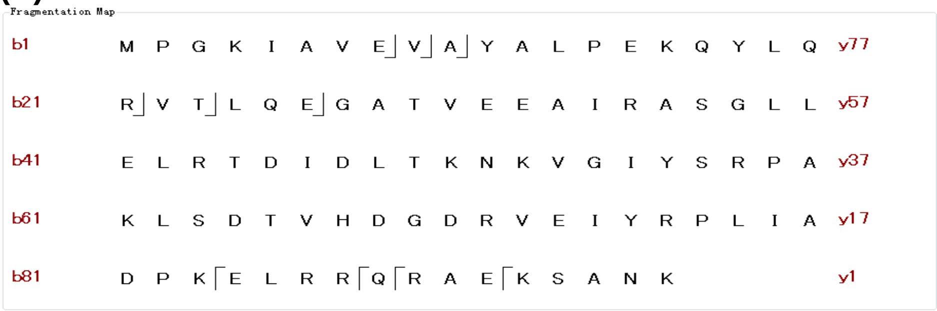

<u>b8-1+O</u>, b9-1+, b10-1+, b21-3+, b23-3+, b26-3+, y5-1+ y8-1+, y9-1+, y13-2+

Figure S4. The graphical fragmentation maps and matching b and y ions of the 4 new proteins idenfied with OIE_CARE. (A), ASR_ECOLU; (B), C562_ECO57; (C), YNFD_ECOLI; (D), RNFH_ECO7I). The underlined b or y ions are unique to OIE_CARE.

Table S1. Matching b/y ions from the forward, random and reverse searches of the three technical replicate HCD spectra of myoglobin.

Forward			Random			Reverse		
**S1**	**S2**	**S3**	**S1**	**S2**	**S3**	**S1**	**S2**	**S3**
**99**	**88**	**88**	**7**	**6**	**9**	**15**	**14**	**15**
b7-1+	b7-1+	b7-1+	b7-1+	b148-14+O	b10-1+	b9-1+	b21-2+O	b21-2+O
b8-1+	b8-1+	b8-1+	b148-14+O	b148-13+	b148-14+	b21-2+O	b26-3+	b26-3+
b9-1+	b9-1+	b9-1+	y8-1+	y8-1+	b151-14+O	b26-3+	b35-4+	b34-4+
b10-1+	b10-1+	b10-1+	y13-2+	y13-2+	y8-1+	b35-4+	b54-6+	b35-4+
b11-1+O	b12-1+	b13-2+	y13-1+	y13-1+	y10-1+	b145-14+	b145-14+	b145-14+
b13-2+	b13-2+	b13-1+	y25-2+	y76-7+	y12-1+O	b146-14+E	b146-14+E	b146-14+E
b13-1+	b13-1+	b14-1+	y152-15+		y13-2+	b146-13+O	b146-13+O	b146-13+
b17-2+	b14-1+	b36-3+O			y76-7+	b147-14+	b147-14+	b147-14+
b20-2+O	b17-2+	b152-15+			y150-14+O	b148-14+	b148-14+	b148-14+
b36-3+O	b18-2+	y6-1+				b151-14+E	b151-14+E	b150-14+
b99-10+	b20-2+O	y7-1+				b152-15+O	b152-15+O	b151-14+E
b146-14+O	b24-3+	y8-1+O				y16-2+	y16-2+	b152-15+O
b151-14+O	b36-3+	y9-1+				y146-14+E	y146-14+E	y16-2+
b152-15+O	b152-15+O	y10-1+E				y151-14+E	y151-14+E	y146-14+E
y6-1+	y6-1+	y12-2+				y152-15+O		y151-14+E
y7-1+	y7-1+	y12-1+						
y8-1+O	y8-1+O	y16-2+						
y9-1+	y12-2+	y17-2+						
y10-1+E	y12-1+	y19-2+O						
y12-2+	y16-2+	y20-2+O						
y12-1+	y17-2+	y21-3+						
y15-2+	y18-2+	y22-3+						

y16-2+	y19-2+O	y22-2+						
y17-2+	y20-3+	y23-3+						
y18-2+O	y20-2+O	y25-3+						
y19-2+O	y21-2+E	y25-2+						
y20-2+O	y22-3+	y26-3+						
y21-2+O	y22-2+O	y27-3+						
y22-3+	y23-3+	y27-2+						
y22-2+O	y24-2+	y28-3+O						
y24-2+	y25-3+	y29-3+						
y25-3+	y25-2+	y30-3+O						
y25-2+	y26-3+	y31-3+O						
y26-3+	y26-2+	y31-2+						
y27-3+	y27-3+	y34-4+						
y27-2+	y28-3+O	y34-3+O						
y28-3+O	y30-3+O	y35-4+O						
y29-3+O	y31-3+	y38-4+O						
y30-3+O	y34-4+	y39-4+						
y31-3+	y34-3+	y39-3+O						
y31-2+	y35-4+O	y40-4+						
y34-4+	y38-4+O	y41-5+						
y34-3+O	y39-4+O	y41-4+O						
y35-4+O	y39-3+O	y42-5+						
y38-4+O	y40-4+	y42-4+O						
y39-4+O	y41-5+	y43-5+						
y39-3+O	y41-4+O	y44-5+						
y40-4+	y41-3+O	y45-5+						
y41-5+	y42-5+	y45-4+						

y42-5+	y42-4+O	y46-5+O						
y42-4+O	y44-5+	y46-4+						
y43-4+	y45-4+	y48-5+						
y44-5+	y46-4+O	y48-4+						
y45-4+	y48-5+	y49-5+O						
y46-4+	y48-4+	y49-4+						
y48-5+	y49-5+O	y50-5+						
y49-5+O	y49-4+	y50-4+						
y49-4+	y50-5+	y51-4+O						
y50-5+	y51-4+O	y52-6+						
y50-4+	y52-6+	y54-6+O						
y51-4+O	y54-6+	y54-5+O						
y52-6+	y54-5+O	y54-4+						
y54-6+	y54-4+O	y66-7+						
y54-5+O	y57-6+O	y66-6+O						
y55-5+	y66-7+	y66-5+						
y57-6+O	y66-6+O	y70-6+						
y57-5+O	y68-7+O	y72-7+O						
y59-5+O	y68-6+O	y74-7+O						
y66-7+	y70-6+	y75-7+E						
y66-6+O	y78-7+O	y81-8+O						
y66-5+	y81-8+O	y81-7+						
y70-6+	y81-7+	y90-9+						
y71-7+O	y90-9+	y103-9+						
y71-6+	y90-8+O	y104-9+O						
y72-7+O	y93-10+	y131-12+O						
y74-7+O	y131-12+O	y136-13+O						

y76-7+O	y144-13+O	y143-14+E						
y78-8+	y145-14+O	y144-14+O						
y81-8+O	y145-13+	y144-13+O						
y81-7+O	y146-14+	y145-14+O						
y90-9+	y147-14+	y145-13+						
y90-8+	y147-13+O	y146-14+O						
y93-10+	y148-14+	y146-13+						
y103-9+	y149-14+	y147-14+O						
y104-9+O	y150-15+E	y149-14+O						
y131-12+O	y150-14+	y150-14+						
y144-14+O	y151-15+O	y151-15+O						
y145-14+O	y151-14+	y151-14+O						
y145-13+								
y146-14+								
y147-15+								
y147-14+O								
y147-13+O								
y148-14+E								
y149-14+O								
y150-14+								
y151-15+								
y151-14+								
y152-15+O								

*"O"=overlapping, which means partial isotopic peaks of this ion are overlapping isotopic peaks; "E"=embedded, which means all isotopic peaks of this ion are overlapping isotopic peaks; "T"=topping, which means the theoretical most abundant isotopic peak (i.e., the relative abundance is 100%) of this ion is shared with other ion(s).

Table S2. Non-matching b/y ions from the forward, random and reverse searches of the three technical replicate HCD spectra of myoglobin.

Forward			Random			Reverse		
**S1**	**S2**	**S3**	**S1**	**S2**	**S3**	**S1**	**S2**	**S3**
378	393	383	294	304	303	345	329	352
b12-1+O	b11-1+O	b11-1+O	b6-1+E	b6-1+E	b6-1+O	b12-1+O	b9-1+O	b6-1+
b12-2+	b19-2+O	b12-1+	b9-1+E	b7-1+T	b7-1+T	b13-1+O	b12-1+O	b12-1+O
b14-1+	b24-2+E	b14-2+O	b24-3+O	b9-1+E	b11-1+	b18-2+T	b16-2+O	b16-2+O
b18-2+O	b32-3+O	b17-2+O	b27-3+O	b11-1+E	b16-2+	b20-2+O	b17-2+O	b18-2+E
b19-2+O	b33-4+O	b18-2+O	b30-3+O	b16-2+	b19-2+O	b23-4+	b18-2+O	b20-2+O
b24-2+E	b35-3+O	b19-2+O	b34-3+	b28-3+O	b30-3+O	b24-3+O	b20-2+O	b23-2+O
b27-2+	b39-4+O	b20-2+O	b36-4+O	b30-3+O	b32-3+O	b26-2+O	b23-4+	b23-4+
b31-4+	b39-5+	b24-2+E	b37-4+O	b36-4+O	b34-3+O	b27-3+O	b24-3+O	b23-3+T
b32-3+O	b41-4+O	b24-3+O	b39-4+O	b37-4+O	b36-4+O	b31-3+O	b26-2+O	b24-3+O
b34-4+E	b42-4+O	b32-3+O	b40-4+O	b38-4+O	b37-4+O	b32-3+E	b27-3+O	b25-2+O
b35-4+E	b42-5+O	b38-4+O	b42-4+O	b39-4+O	b38-4+O	b33-3+O	b29-3+O	b26-2+O
b38-4+O	b44-4+O	b41-4+O	b42-5+O	b40-4+O	b39-4+O	b34-3+O	b31-3+O	b27-3+O
b39-5+O	b45-4+O	b42-4+O	b45-5+O	b42-5+O	b40-4+O	b34-4+O	b32-3+E	b29-3+O
b41-3+	b45-5+O	b42-5+O	b46-4+O	b46-5+O	b42-4+O	b35-3+O	b33-3+O	b30-3+E
b42-4+O	b46-5+O	b43-5+O	b49-5+O	b49-5+O	b42-5+O	b37-4+O	b34-3+O	b31-3+O
b43-5+O	b46-6+	b44-4+O	b50-5+O	b50-5+O	b43-4+O	b39-4+O	b34-4+O	b32-3+O
b44-4+O	b47-4+O	b45-5+O	b51-5+O	b52-6+O	b45-4+O	b41-5+O	b35-3+O	b33-3+O
b45-4+O	b47-5+O	b46-5+O	b54-5+O	b54-5+O	b45-5+O	b42-5+	b37-4+O	b34-3+O
b46-6+O	b49-5+O	b46-6+O	b55-5+O	b55-6+O	b46-5+O	b43-4+O	b38-4+O	b35-3+O
b47-4+O	b50-5+O	b47-4+O	b55-6+O	b56-6+O	b49-5+O	b43-5+O	b39-4+O	b38-5+E
b47-5+O	b50-6+O	b47-5+O	b57-6+O	b57-6+O	b50-5+O	b44-4+O	b41-4+O	b41-4+O
b49-5+O	b51-5+O	b49-5+O	b58-6+O	b58-6+O	b51-5+O	b49-4+O	b43-4+O	b41-5+O

b51-5+O	b52-6+O	b49-6+O	b59-7+O	b59-7+O	b53-6+O	b49-5+O	b43-5+	b42-5+O
b51-7+O	b53-6+O	b52-6+O	b59-11+	b59-11+O	b54-5+O	b50-6+O	b45-4+O	b43-4+O
b53-6+O	b54-5+O	b53-6+O	b62-5+	b61-7+O	b57-6+O	b52-5+O	b47-4+O	b46-5+O
b54-5+O	b54-6+O	b54-5+O	b62-6+O	b62-6+O	b58-6+O	b52-6+E	b47-5+O	b49-4+O
b56-6+O	b55-5+O	b54-6+O	b63-9+O	b63-9+O	b59-7+O	b54-6+O	b48-4+E	b49-5+O
b57-5+O	b56-6+O	b55-5+O	b64-7+O	b65-6+O	b59-11+	b55-5+O	b49-4+O	b50-6+O
b59-6+O	b56-7+	b57-5+O	b66-7+O	b65-7+O	b62-6+O	b57-6+O	b49-5+O	b51-5+T
b60-5+O	b56-10+	b60-5+O	b68-7+O	b66-8+O	b62-8+	b58-5+O	b50-6+O	b52-5+O
b60-6+O	b57-5+O	b60-6+O	b68-12+	b68-7+O	b62-10+	b58-6+O	b52-5+O	b52-6+O
b61-5+O	b59-5+O	b61-5+O	b69-6+O	b68-12+	b63-7+O	b59-5+O	b52-6+O	b55-5+O
b61-6+O	b60-5+O	b61-6+O	b69-10+O	b69-10+O	b63-9+O	b60-6+O	b57-6+O	b57-5+O
b61-7+O	b60-6+O	b62-7+O	b69-5+T	b70-8+O	b64-6+O	b61-6+O	b57-10+	b57-6+O
b62-5+O	b61-5+	b63-5+O	b70-8+O	b73-6+O	b65-6+O	b61-7+O	b58-5+O	b58-6+O
b63-7+O	b61-6+O	b63-7+O	b71-8+O	b73-8+O	b66-7+O	b62-6+O	b58-6+O	b59-5+O
b64-7+O	b61-7+O	b64-7+O	b73-6+O	b74-10+O	b68-7+O	b65-6+O	b60-6+O	b59-7+O
b65-6+O	b63-5+O	b65-8+E	b73-7+O	b75-8+O	b68-12+	b66-7+O	b61-6+O	b60-5+O
b65-7+O	b63-6+O	b68-5+E	b73-8+O	b78-8+O	b69-10+O	b67-6+O	b62-5+E	b60-6+O
b65-8+E	b63-7+O	b69-6+O	b73-10+O	b78-10+	b73-6+	b67-7+O	b62-6+O	b61-6+O
b66-7+O	b64-7+O	b69-7+O	b74-10+	b78-11+	b73-8+O	b67-8+O	b62-7+O	b62-6+O
b68-8+O	b65-6+O	b69-8+O	b75-8+O	b79-8+O	b74-10+	b68-7+O	b63-6+O	b64-6+O
b69-6+O	b65-8+T	b70-6+O	b75-10+O	b79-9+O	b75-8+O	b69-7+O	b65-6+O	b67-6+O
b69-7+O	b66-7+O	b70-7+O	b77-9+O	b80-8+O	b77-6+	b70-6+O	b67-6+O	b67-8+O
b70-6+O	b67-6+O	b70-8+O	b78-8+O	b82-7+E	b78-8+O	b71-7+O	b67-7+O	b67-11+
b70-7+O	b68-5+E	b71-6+O	b78-11+E	b83-13+	b78-11+	b72-7+O	b67-8+	b68-8+O
b71-6+O	b69-6+O	b71-8+O	b79-8+O	b84-9+O	b79-8+O	b73-8+M	b69-7+O	b70-6+O
b71-7+O	b69-7+O	b72-7+O	b80-8+O	b84-12+T	b80-8+O	b74-9+M	b70-6+O	b71-7+O
b71-8+O	b69-8+O	b73-6+O	b82-7+O	b85-10+O	b81-8+T	b74-10+M	b71-7+O	b72-7+O

b74-6+O	b70-6+O	b75-7+	b83-13+	b86-8+O	b82-7+O	b75-7+O	b72-7+O	b73-7+O
b74-7+O	b70-7+O	b77-13+O	b84-6+O	b86-9+O	b82-8+O	b77-9+O	b74-9+M	b74-9+M
b75-7+O	b71-6+O	b78-7+O	b84-12+T	b86-15+	b82-10+	b77-10+O	b75-7+O	b75-7+O
b77-7+O	b71-7+O	b78-8+O	b86-7+O	b87-15+	b83-13+	b77-12+O	b75-8+O	b76-7+O
b77-13+O	b71-9+O	b80-8+O	b86-15+	b88-8+O	b84-9+O	b78-7+O	b77-9+O	b77-9+O
b78-8+O	b71-8+T	b81-6+O	b87-15+	b88-9+O	b84-12+T	b78-9+O	b78-7+O	b78-7+O
b78-10+O	b73-6+O	b81-7+O	b88-8+O	b91-8+O	b85-8+O	b79-8+M	b78-9+O	b78-9+O
b81-6+O	b74-6+O	b81-8+O	b88-9+O	b91-9+O	b86-8+O	b79-9+M	b79-8+M	b79-8+M
b81-7+O	b77-13+O	b81-9+O	b89-9+O	b91-12+	b86-9+O	b80-7+M	b80-8+M	b80-8+M
b81-8+O	b78-7+O	b82-8+O	b90-10+O	b92-8+O	b86-15+	b80-8+M	b80-11+M	b80-11+M
b81-9+O	b78-8+O	b83-9+O	b91-8+O	b92-9+O	b87-15+O	b80-11+M	b81-7+O	b81-7+O
b82-8+O	b80-7+O	b83-11+T	b91-9+O	b93-9+O	b88-9+O	b81-7+O	b81-8+O	b81-8+O
b83-8+O	b81-6+O	b84-7+O	b91-10+O	b94-12+O	b91-8+O	b81-8+O	b82-7+O	b82-7+O
b83-9+O	b81-8+O	b84-10+O	b91-12+	b95-10+O	b91-9+O	b83-8+O	b83-8+O	b82-9+O
b83-11+O	b81-9+O	b85-8+O	b92-9+O	b95-12+O	b91-12+	b83-13+	b84-8+O	b83-8+O
b84-8+O	b82-8+O	b86-8+O	b93-8+O	b96-8+O	b92-8+O	b84-7+O	b85-8+O	b84-8+O
b84-9+	b82-9+O	b86-9+O	b93-9+O	b96-9+O	b92-9+O	b85-8+O	b85-12+	b85-8+O
b84-10+O	b84-8+O	b87-8+O	b93-10+O	b96-10+O	b92-12+O	b85-9+O	b86-8+O	b86-7+O
b86-8+O	b84-10+O	b87-9+O	b95-12+O	b96-12+O	b93-8+O	b85-12+O	b86-9+O	b86-8+O
b86-9+O	b85-8+O	b88-9+O	b96-8+O	b99-9+O	b93-9+O	b86-7+O	b86-11+	b87-7+O
b87-7+O	b85-10+O	b89-8+O	b96-9+O	b100-10+O	b93-11+O	b86-8+O	b87-7+O	b89-8+O
b87-8+O	b86-8+O	b89-15+	b96-12+O	b101-9+O	b95-12+O	b86-11+	b88-9+O	b89-9+O
b87-9+O	b86-9+O	b90-7+O	b100-10+O	b101-10+O	b96-8+O	b89-8+O	b89-8+O	b89-12+O
b88-9+O	b87-7+O	b91-9+O	b101-9+O	b102-9+O	b96-9+O	b89-9+O	b89-9+O	b90-9+O
b88-12+	b87-9+O	b92-8+O	b101-10+O	b103-9+O	b96-10+O	b89-12+O	b89-12+O	b91-7+
b89-8+O	b89-8+O	b92-10+O	b101-11+O	b103-12+O	b96-12+O	b90-8+O	b90-8+O	b91-9+O
b89-15+O	b89-9+O	b93-9+O	b102-8+	b103-13+O	b100-8+	b90-9+O	b91-9+O	b92-7+

b90-7+	b89-10+O	b93-10+O	b103-9+O	b104-12+	b100-10+O	b91-9+O	b92-9+O	b92-9+O
b91-8+O	b89-15+O	b94-10+O	b103-11+O	b105-10+O	b101-9+O	b91-10+E	b95-8+O	b92-10+O
b91-9+O	b90-7+	b95-9+O	b103-13+O	b106-8+O	b101-10+O	b92-8+O	b95-9+O	b93-8+O
b92-8+T	b90-10+O	b96-9+O	b104-12+	b106-10+O	b102-8+O	b92-9+O	b95-10+O	b93-12+O
b93-9+O	b91-8+O	b97-9+O	b105-9+O	b106-11+O	b102-9+O	b93-8+O	b97-9+O	b95-9+O
b94-8+O	b91-9+O	b97-10+O	b105-10+O	b106-12+O	b103-9+O	b93-10+O	b98-9+O	b95-10+O
b95-9+O	b92-10+T	b98-9+O	b106-8+O	b108-9+O	b103-13+O	b95-9+O	b99-12+O	b97-9+O
b96-8+O	b94-8+O	b98-10+O	b106-10+O	b108-12+O	b105-10+O	b95-10+O	b101-10+O	b99-13+
b96-9+O	b96-9+O	b99-9+O	b106-11+O	b110-10+O	b106-8+O	b95-14+	b103-9+O	b101-8+O
b97-9+O	b96-10+O	b99-10+O	b106-12+O	b110-11+O	b106-10+O	b96-8+O	b103-10+O	b101-9+O
b97-10+O	b97-9+O	b99-13+O	b107-11+O	b111-9+O	b106-11+O	b97-9+O	b103-11+O	b101-10+O
b98-9+O	b97-10+O	b100-12+O	b108-15+O	b111-10+O	b106-12+O	b99-9+O	b103-14+T	b102-9+O
b99-9+O	b98-9+O	b103-8+	b110-10+O	b111-11+O	b107-11+O	b99-10+O	b104-10+O	b102-15+
b99-13+O	b99-9+O	b103-9+O	b111-10+O	b111-12+O	b107-12+O	b100-11+O	b105-9+O	b103-10+O
b100-12+O	b99-10+O	b103-10+O	b111-11+O	b112-13+O	b108-12+O	b101-8+O	b105-10+O	b103-11+O
b103-11+O	b99-13+O	b103-11+O	b111-12+O	b112-14+	b110-10+O	b101-9+O	b105-11+O	b103-14+O
b104-9+O	b100-12+O	b104-9+O	b111-9+T	b113-12+O	b110-11+O	b101-10+O	b106-9+O	b104-9+O
b104-10+O	b103-11+O	b104-10+O	b112-13+O	b114-10+O	b111-10+O	b101-12+O	b107-10+O	b104-10+O
b105-10+O	b104-10+O	b105-10+O	b114-10+O	b115-11+O	b111-11+O	b103-9+O	b109-9+O	b105-10+O
b105-11+O	b105-10+O	b105-11+O	b115-11+O	b115-13+O	b111-12+O	b103-10+O	b109-12+	b105-11+O
b106-9+O	b105-11+O	b106-9+O	b116-10+O	b116-11+O	b111-9+T	b103-11+O	b111-10+O	b106-10+O
b106-13+O	b106-9+E	b106-10+O	b116-11+O	b116-12+O	b112-10+O	b103-14+T	b111-12+O	b106-11+O
b107-10+O	b106-10+O	b106-13+O	b116-12+O	b123-11+O	b112-13+O	b104-10+O	b112-12+O	b107-10+O
b109-9+O	b106-12+O	b107-10+O	b117-12+O	b125-11+O	b114-10+O	b105-9+O	b112-13+O	b108-12+E
b109-10+O	b107-10+O	b109-9+O	b120-10+O	b125-13+O	b115-11+O	b105-10+O	b113-12+O	b109-9+O
b109-11+O	b108-11+O	b109-10+O	b120-14+O	b126-13+O	b115-13+O	b105-11+O	b114-12+O	b109-11+O
b110-9+O	b109-9+E	b109-11+O	b124-13+O	b127-13+O	b116-10+O	b106-9+O	b114-14+O	b111-10+O

b110-11+O	b109-10+O	b110-9+O	b125-11+O	b128-12+O	b116-11+O	b106-10+O	b115-11+O	b112-10+O
b111-10+O	b109-11+O	b111-10+O	b125-13+O	b129-11+O	b116-12+O	b107-10+O	b115-14+O	b112-12+O
b111-11+O	b110-9+	b111-11+O	b125-14+O	b129-13+O	b117-10+O	b108-9+O	b117-10+O	b112-13+
b112-9+E	b110-11+O	b112-9+E	b126-11+O	b129-14+O	b117-13+O	b109-12+E	b117-12+O	b113-12+O
b112-10+O	b111-10+O	b112-10+O	b127-13+O	b130-11+O	b120-14+O	b110-10+O	b118-10+O	b114-12+O
b112-11+O	b111-11+O	b112-11+O	b128-12+O	b130-12+O	b123-11+O	b111-10+O	b118-11+O	b114-14+O
b112-12+O	b112-9+O	b112-12+O	b129-11+O	b130-13+O	b124-12+O	b111-12+O	b118-13+O	b115-10+O
b113-10+O	b112-10+O	b113-10+O	b130-12+O	b130-14+O	b125-11+O	b112-10+O	b119-14+O	b115-11+O
b113-14+	b112-11+O	b114-10+O	b130-13+O	b131-13+O	b125-13+O	b112-13+O	b120-10+O	b115-12+O
b114-10+O	b112-12+O	b115-10+O	b131-13+O	b132-13+O	b126-11+O	b114-12+O	b120-11+O	b115-14+O
b115-13+O	b113-10+O	b115-11+O	b133-13+O	b133-13+O	b126-13+O	b114-14+O	b120-12+O	b117-10+O
b116-10+O	b113-14+O	b115-12+O	b136-13+O	b133-14+E	b128-12+O	b115-10+O	b120-13+O	b117-11+O
b116-12+O	b114-10+O	b116-10+O	b136-14+O	b134-12+O	b129-11+O	b115-11+O	b121-11+O	b117-12+O
b116-14+	b116-10+O	b116-12+O	b138-12+O	b136-13+O	b130-12+O	b115-14+	b121-14+O	b118-10+O
b116-15+T	b116-12+O	b116-15+T	b138-13+O	b137-14+O	b130-13+O	b117-10+O	b122-11+O	b118-11+O
b118-10+O	b116-15+T	b120-10+O	b138-15+O	b139-12+O	b131-12+O	b117-12+O	b122-12+O	b118-15+E
b118-12+O	b118-10+O	b120-11+O	b139-12+O	b139-14+O	b131-13+O	b118-11+O	b123-10+O	b119-11+O
b119-12+O	b118-13+O	b121-11+O	b139-14+O	b140-12+O	b131-14+O	b119-11+O	b123-11+O	b119-14+O
b119-13+O	b119-12+O	b121-12+O	b140-12+O	b141-12+O	b133-13+O	b119-14+O	b123-13+O	b120-11+O
b120-10+O	b119-13+O	b122-13+O	b140-14+O	b141-13+O	b133-14+O	b120-11+O	b123-14+O	b120-12+O
b120-11+O	b120-11+O	b123-12+O	b140-15+O	b141-14+O	b134-12+O	b120-12+O	b124-11+O	b120-13+O
b121-11+O	b121-11+O	b124-13+O	b141-12+O	b142-12+O	b135-12+O	b121-14+E	b125-11+O	b121-11+O
b121-12+O	b121-12+O	b124-14+O	b141-13+O	b142-13+O	b136-13+O	b122-12+O	b125-12+O	b122-10+O
b122-13+T	b122-13+T	b125-14+O	b141-14+O	b142-14+O	b136-15+O	b122-13+O	b126-10+	b122-11+O
b125-11+O	b124-14+O	b126-12+O	b142-12+O	b143-15+O	b138-12+O	b123-11+O	b126-11+O	b122-12+O
b125-14+O	b127-15+O	b126-13+O	b142-14+O	b144-15+O	b138-15+O	b123-13+O	b126-13+O	b122-13+O
b126-11+O	b128-12+O	b127-15+O	b143-12+O	b145-14+O	b139-12+O	b124-11+O	b127-12+O	b123-11+O

b126-14+O	b129-11+O	b128-12+O	b143-13+O	b146-12+O	b139-14+O	b126-12+O	b128-11+O	b123-13+O
b127-15+O	b129-13+O	b129-11+O	b143-15+O	b146-13+O	b140-12+O	b126-13+O	b128-13+O	b124-11+O
b128-12+O	b130-11+O	b129-13+O	b145-14+O	b146-15+O	b140-15+O	b127-12+O	b129-12+O	b125-11+O
b129-11+O	b130-12+O	b130-11+O	b146-13+O	b147-13+O	b141-12+O	b127-13+O	b129-13+O	b126-12+O
b129-13+O	b130-13+O	b130-12+O	b146-14+O	b148-15+O	b141-13+O	b129-12+O	b130-12+O	b127-12+O
b130-11+O	b130-14+O	b130-13+O	b148-13+O	b149-13+O	b141-14+O	b129-15+E	b131-14+O	b128-13+O
b130-12+O	b131-10+O	b130-14+O	b148-15+O	b149-14+O	b141-15+O	b130-12+O	b132-12+O	b129-13+O
b130-13+O	b131-14+O	b131-10+O	b149-13+O	b149-15+O	b142-12+O	b131-11+O	b132-13+O	b130-12+O
b130-14+O	b131-15+O	b131-11+O	b149-15+O	b150-13+O	b142-14+O	b131-13+O	b133-12+O	b131-13+O
b131-11+O	b132-11+O	b131-12+O	b150-13+O	b151-15+O	b143-13+O	b131-14+O	b134-12+O	b132-12+O
b131-12+O	b133-12+O	b131-13+O	b151-13+O	b152-14+O	b143-14+O	b132-12+O	b135-13+O	b132-13+O
b131-13+O	b133-13+O	b131-14+O	b151-14+O	b152-15+O	b143-15+O	b132-13+O	b135-14+O	b132-14+O
b131-15+O	b134-12+M	b132-11+O	b151-15+O	y11-1+E	b144-14+O	b133-12+O	b136-12+O	b133-11+O
b132-11+O	b134-13+M	b133-12+O	b152-14+O	y20-2+O	b144-15+O	b135-13+O	b136-13+O	b133-12+O
b132-15+O	b134-14+M	b134-12+M	b152-15+O	y21-2+O	b145-14+O	b136-13+O	b136-14+O	b134-11+E
b133-13+O	b134-15+M	b134-13+M	y11-1+E	y22-2+O	b146-13+O	b137-14+O	b137-14+O	b134-12+O
b134-12+M	b135-12+O	b134-14+O	y20-2+O	y25-2+O	b148-13+O	b138-15+O	b138-11+O	b135-11+O
b134-13+M	b135-13+O	b134-15+O	y22-2+O	y26-3+O	b149-13+O	b139-12+O	b138-14+O	b135-12+O
b134-15+M	b136-14+O	b135-12+O	y23-2+O	y32-4+	b149-15+O	b139-13+O	b139-11+O	b136-12+O
b135-12+O	b137-12+O	b136-13+O	y26-3+O	y34-3+O	b150-14+O	b140-11+O	b139-12+O	b136-13+O
b135-13+O	b137-14+O	b139-14+O	y32-4+O	y34-4+E	b151-13+O	b140-12+O	b139-13+O	b137-14+O
b135-14+O	b138-12+O	b140-12+O	y34-3+O	y36-3+O	b151-15+O	b140-13+O	b139-14+O	b138-14+O
b136-12+O	b139-14+O	b140-14+O	y36-3+O	y37-4+O	b152-14+O	b140-14+O	b140-11+O	b138-15+O
b136-14+O	b140-12+O	b141-12+O	y36-5+E	y38-3+O	b152-15+O	b141-12+O	b140-12+O	b139-11+
b140-12+O	b140-14+O	b141-13+O	y37-4+O	y43-4+O	y11-1+E	b141-13+O	b140-13+O	b139-12+O
b140-14+O	b142-12+O	b142-12+O	y38-3+O	y44-5+O	y13-1+O	b141-14+O	b140-14+O	b139-13+O
b141-13+O	b142-13+O	b142-13+O	y40-4+O	y45-4+O	y20-2+O	b141-15+O	b141-13+O	b139-14+O

b142-12+O	b142-14+O	b142-15+O	y43-4+O	y45-5+O	y22-2+O	b142-13+O	b141-14+O	b140-11+O
b142-13+O	b143-15+O	b143-13+O	y44-5+O	y48-4+O	y25-2+O	b142-14+O	b141-15+O	b140-12+O
b142-15+O	b144-11+O	b143-15+O	y45-4+O	y49-4+O	y31-3+O	b143-13+O	b143-13+O	b140-13+O
b143-13+O	b144-13+O	b144-11+O	y46-4+O	y50-4+O	y32-4+O	b145-13+O	b144-12+O	b140-14+O
b143-15+O	b144-14+O	b144-13+O	y48-4+O	y50-6+O	y34-3+O	b147-13+O	b145-13+O	b141-13+O
b144-13+O	b145-14+O	b144-14+O	y49-4+O	y51-5+O	y36-3+O	b147-15+O	b147-13+O	b141-14+O
b144-14+O	b145-15+O	b145-14+O	y50-4+O	y53-5+O	y37-4+O	b148-15+O	b147-15+O	b142-13+O
b145-13+O	b147-13+O	b146-12+O	y50-6+O	y55-5+O	y41-4+O	b149-13+M	b148-13+O	b142-14+O
b145-14+O	b147-14+O	b147-13+O	y51-5+O	y55-6+O	y43-4+O	b149-14+M	b148-15+O	b142-15+O
b147-12+O	b147-15+O	b147-14+O	y52-5+O	y57-5+O	y45-4+O	b150-15+O	b149-12+M	b143-13+O
b147-14+O	b148-13+O	b148-13+O	y53-5+O	y57-6+O	y45-5+O	b151-15+O	b149-14+M	b144-12+O
b148-13+O	b148-14+O	b148-14+O	y55-5+O	y59-10+	y48-4+O	y11-1+O	b150-15+O	b144-14+O
b148-14+O	b148-15+O	b148-15+O	y55-6+O	y60-5+O	y50-4+O	y16-1+O	b151-15+O	b146-15+O
b148-15+O	b149-13+O	b149-14+O	y57-5+O	y61-6+O	y50-5+O	y18-2+E	b152-14+O	b147-13+O
b149-15+O	b149-14+O	b149-15+O	y60-5+O	y62-7+O	y51-5+O	y22-2+T	y11-1+E	b147-15+O
b150-15+O	b149-15+O	b150-15+O	y62-7+	y64-6+O	y53-5+O	y25-3+O	y16-1+	b148-15+O
b151-15+O	b150-14+O	b151-14+O	y64-6+O	y65-6+O	y55-5+O	y29-3+O	y18-2+T	b149-13+M
y13-1+O	b150-15+O	b151-15+O	y65-6+O	y65-7+O	y55-6+O	y30-3+O	y22-2+E	b149-14+M
y17-3+O	b151-15+O	y13-1+O	y66-5+E	y66-5+E	y57-5+O	y31-4+O	y25-3+O	b150-15+O
y20-3+O	b152-14+O	y15-2+O	y66-6+O	y66-7+O	y60-5+O	y34-3+T	y30-3+O	b151-15+O
y21-3+O	y15-2+	y17-3+O	y66-7+O	y68-7+O	y60-6+O	y38-4+O	y38-4+E	y11-1+O
y23-2+O	y17-3+	y18-2+O	y68-7+O	y69-6+O	y64-5+O	y39-4+O	y39-4+O	y16-1+
y23-3+O	y21-3+	y23-2+O	y69-6+O	y69-10+	y64-6+O	y39-5+	y39-5+	y18-2+T
y24-3+O	y23-2+O	y24-2+O	y69-8+O	y70-6+O	y65-6+O	y40-3+	y40-4+O	y22-2+E
y26-2+O	y24-3+O	y24-3+O	y69-10+	y71-6+O	y65-7+O	y40-6+O	y40-6+O	y25-3+O
y26-4+	y26-4+	y26-2+O	y70-6+O	y71-7+O	y66-5+E	y41-4+O	y41-4+O	y29-3+O
y27-4+O	y27-2+O	y26-4+O	y71-6+O	y72-6+O	y66-7+O	y42-4+E	y42-4+O	y30-3+O

y28-2+	y27-4+	y27-4+	y71-7+O	y73-7+O	y68-7+O	y43-5+O	y43-5+O	y32-4+O
y30-2+	y28-2+	y28-2+O	y72-7+O	y75-6+O	y69-8+O	y45-4+O	y45-5+O	y39-4+O
y31-4+O	y28-4+O	y29-2+	y72-10+T	y75-7+O	y69-10+	y46-5+E	y46-5+E	y40-3+
y32-3+O	y29-3+O	y30-2+	y72-6+T	y75-8+O	y70-6+O	y48-5+E	y49-6+O	y40-4+O
y33-3+O	y30-2+	y32-3+O	y73-6+O	y77-7+O	y71-6+O	y49-6+O	y51-4+O	y40-6+O
y34-2+O	y31-2+	y33-3+O	y74-6+O	y80-7+O	y71-7+O	y51-4+O	y51-6+O	y41-4+O
y34-5+	y32-3+O	y34-2+O	y75-6+O	y81-7+O	y72-6+O	y51-5+O	y52-6+O	y42-4+O
y35-3+O	y33-3+O	y34-5+O	y75-8+O	y81-9+	y72-7+O	y51-6+O	y53-6+O	y43-5+O
y36-3+O	y34-2+O	y35-3+O	y76-6+O	y83-7+O	y73-7+O	y52-5+O	y54-5+O	y44-6+O
y36-4+O	y34-5+O	y36-4+O	y76-7+O	y83-8+O	y75-6+O	y52-6+O	y56-6+O	y45-5+O
y37-4+O	y36-3+O	y37-4+O	y76-8+O	y83-9+O	y77-6+O	y53-6+O	y58-5+O	y49-6+O
y38-3+O	y37-4+O	y38-3+O	y77-7+O	y84-7+O	y77-7+O	y54-5+O	y59-6+O	y51-4+O
y40-3+O	y38-3+O	y39-5+	y77-6+T	y84-8+O	y78-9+	y56-5+O	y60-5+	y51-6+O
y40-5+	y40-3+O	y40-3+O	y78-9+	y85-7+O	y78-10+	y58-5+O	y60-6+O	y52-6+O
y41-3+O	y40-5+O	y40-5+O	y81-7+O	y85-8+O	y81-7+O	y59-6+O	y61-6+O	y53-6+O
y41-4+O	y42-3+O	y41-3+O	y81-8+O	y86-7+O	y81-8+O	y60-5+	y61-8+O	y54-5+O
y42-3+O	y43-4+O	y42-3+O	y83-8+O	y88-7+O	y82-8+O	y60-6+O	y63-6+O	y54-9+
y43-5+O	y43-5+O	y43-4+O	y84-8+O	y88-8+O	y83-7+O	y61-6+O	y65-6+O	y56-5+O
y44-4+O	y44-3+	y44-3+E	y84-7+	y88-9+O	y83-9+O	y61-8+O	y66-6+O	y56-6+O
y44-6+	y44-4+O	y44-4+O	y85-8+O	y89-8+O	y84-7+O	y63-6+O	y67-7+O	y58-5+O
y45-5+O	y44-6+	y44-6+	y88-8+O	y91-9+O	y84-8+O	y63-10+	y69-6+O	y59-6+O
y46-5+O	y45-5+O	y45-7+	y89-8+O	y92-12+O	y84-9+O	y65-6+O	y70-6+O	y60-5+
y47-4+O	y45-7+	y46-3+O	y89-9+O	y93-9+O	y85-8+O	y66-6+O	y74-9+O	y60-6+O
y47-5+O	y46-5+O	y47-4+O	y92-9+O	y93-10+O	y85-9+O	y67-8+	y75-7+O	y60-7+O
y48-4+O	y47-4+O	y47-5+O	y92-12+O	y94-8+O	y86-7+O	y69-6+O	y76-8+O	y61-6+O
y49-8+O	y47-5+O	y51-5+E	y94-9+O	y94-9+O	y88-7+O	y70-6+O	y77-6+	y61-8+O
y51-5+E	y50-4+O	y52-4+O	y94-10+O	y94-10+O	y88-8+O	y73-8+O	y77-8+O	y63-6+O

y51-6+O	y51-6+O	y53-5+O	y95-7+	y95-7+O	y89-7+O	y74-9+O	y77-9+O	y64-6+O
y52-4+O	y52-4+O	y55-4+O	y95-8+O	y95-9+O	y89-8+O	y74-10+	y77-10+O	y65-6+O
y54-4+O	y53-5+O	y55-5+O	y95-9+O	y96-11+O	y89-9+O	y76-8+O	y78-7+O	y66-6+O
y55-4+	y53-6+O	y55-6+O	y96-11+O	y97-7+	y91-9+O	y77-8+O	y78-8+O	y69-7+O
y55-6+O	y55-4+	y56-6+O	y97-14+	y97-9+O	y92-9+O	y77-9+O	y79-8+O	y70-6+O
y56-6+O	y55-5+O	y57-4+O	y99-9+O	y97-11+O	y93-10+O	y77-10+E	y80-8+O	y71-8+O
y57-4+	y55-6+O	y57-5+O	y100-10+O	y97-14+	y94-9+O	y77-6+T	y80-11+E	y72-7+O
y57-7+O	y56-6+O	y59-7+E	y100-13+E	y99-9+O	y95-7+O	y79-8+O	y81-8+O	y74-9+O
y58-6+O	y57-5+O	y60-5+O	y102-8+O	y100-13+E	y96-11+O	y79-9+O	y82-7+O	y75-7+O
y59-6+O	y57-7+O	y60-6+O	y102-9+O	y101-8+O	y97-9+O	y80-7+O	y82-8+O	y75-8+O
y60-5+O	y58-5+O	y61-6+O	y102-10+O	y102-8+O	y97-14+	y80-8+O	y83-8+O	y76-8+O
y60-6+O	y58-6+O	y62-6+O	y103-10+O	y102-9+O	y98-8+O	y80-11+E	y84-8+O	y77-6+O
y61-5+O	y59-5+O	y62-7+O	y104-9+O	y102-10+O	y99-9+O	y82-7+O	y84-9+O	y77-8+O
y61-6+O	y59-6+O	y64-6+O	y104-10+O	y103-10+O	y99-10+O	y82-8+O	y85-7+O	y77-9+O
y61-7+O	y60-5+O	y65-6+O	y105-9+O	y104-9+O	y100-10+O	y83-8+O	y85-8+O	y79-8+O
y62-6+O	y60-6+O	y67-6+O	y105-10+O	y104-10+O	y100-13+	y84-9+O	y86-8+O	y80-8+O
y64-6+O	y61-6+O	y68-6+O	y106-10+O	y104-11+O	y101-10+O	y85-7+E	y86-9+O	y80-11+E
y65-6+O	y62-6+O	y68-7+O	y107-9+O	y105-10+O	y102-8+O	y85-8+O	y86-13+O	y82-7+O
y67-6+O	y62-7+O	y68-9+O	y107-10+O	y106-9+O	y102-10+O	y86-8+O	y87-8+O	y82-8+O
y67-7+O	y64-6+O	y69-6+O	y107-11+O	y106-10+O	y103-10+O	y86-9+O	y87-9+O	y83-8+O
y68-7+O	y65-6+O	y71-6+O	y108-9+O	y106-11+O	y104-9+O	y86-13+O	y88-9+O	y83-10+O
y68-9+O	y65-7+O	y71-8+O	y108-10+O	y107-10+O	y104-10+O	y87-8+O	y89-12+	y84-8+O
y69-6+O	y66-5+O	y72-8+O	y110-11+O	y108-9+O	y105-9+O	y88-9+O	y90-9+O	y85-7+E
y70-7+O	y67-6+O	y73-7+O	y110-12+O	y109-11+O	y105-10+O	y89-12+	y91-7+E	y85-8+O
y70-8+O	y67-7+O	y74-6+O	y111-10+O	y110-10+O	y106-10+O	y90-9+O	y91-11+O	y86-8+O
y72-8+O	y68-9+O	y74-8+O	y112-9+O	y110-11+O	y107-10+O	y91-7+E	y93-8+O	y86-9+O
y74-8+O	y69-6+O	y75-8+O	y112-10+O	y110-12+O	y107-11+O	y91-11+O	y93-9+O	y86-13+O

y75-8+O	y70-8+O	y76-7+O	y114-11+O	y111-9+O	y108-10+O	y93-8+O	y94-8+O	y87-8+O
y76-8+O	y71-8+O	y76-8+O	y115-10+O	y111-10+O	y109-9+O	y93-9+O	y95-9+O	y87-9+O
y77-7+O	y72-8+O	y77-6+O	y115-11+O	y112-10+O	y110-11+O	y94-8+O	y95-10+O	y88-9+O
y77-9+O	y73-7+O	y77-7+O	y115-12+O	y114-11+O	y110-12+O	y95-9+O	y95-11+O	y89-12+O
y78-7+O	y74-6+O	y77-9+E	y116-11+O	y114-13+	y111-9+O	y95-11+O	y96-9+O	y89-13+O
y78-9+O	y74-7+O	y78-7+O	y117-10+O	y115-10+O	y111-10+O	y96-9+O	y99-9+O	y91-7+E
y79-7+O	y74-8+O	y78-8+O	y117-11+O	y116-10+O	y111-11+O	y96-12+	y99-10+O	y91-11+O
y79-8+O	y75-8+O	y78-9+O	y117-12+O	y116-11+O	y111-15+O	y97-10+O	y99-11+	y93-8+O
y79-9+O	y76-7+O	y79-7+O	y118-11+O	y117-10+O	y112-10+O	y99-9+O	y101-8+O	y93-9+O
y80-8+O	y76-8+O	y80-8+O	y119-10+O	y117-11+O	y114-11+O	y99-10+O	y101-13+O	y95-9+O
y80-9+O	y76-9+O	y81-9+O	y119-11+O	y117-12+O	y114-13+O	y99-11+	y102-9+O	y95-10+O
y81-9+O	y77-6+O	y81-10+	y119-12+O	y118-11+O	y116-11+O	y100-9+O	y103-9+O	y95-11+O
y82-7+O	y77-7+O	y82-7+O	y120-11+O	y119-10+O	y117-10+O	y102-10+O	y103-10+O	y96-9+O
y83-8+O	y77-9+O	y82-9+O	y120-12+O	y119-11+O	y117-11+O	y103-10+O	y103-12+O	y97-10+O
y84-7+O	y78-8+O	y83-7+O	y121-11+O	y119-12+O	y118-10+O	y103-12+O	y104-9+O	y99-9+O
y84-8+O	y78-9+O	y83-8+O	y121-13+O	y120-11+O	y118-11+O	y104-9+O	y104-10+O	y99-10+O
y84-9+O	y79-7+O	y83-9+O	y122-12+O	y120-12+O	y119-11+O	y104-10+O	y104-11+O	y99-11+
y85-7+O	y79-8+O	y84-7+O	y122-13+O	y121-11+O	y119-12+O	y105-11+O	y106-8+	y100-9+O
y85-8+O	y79-9+O	y84-8+O	y123-13+O	y121-12+O	y120-11+O	y105-12+O	y106-9+O	y100-11+O
y86-7+O	y80-7+O	y84-9+O	y124-11+O	y122-11+O	y120-12+O	y106-9+O	y106-10+O	y101-13+O
y86-8+O	y80-8+O	y85-8+O	y124-12+O	y124-10+O	y121-11+O	y106-10+O	y106-11+O	y103-9+O
y86-11+O	y80-9+O	y85-9+O	y127-12+O	y124-11+O	y122-11+O	y106-11+O	y106-12+O	y103-10+O
y87-8+O	y82-7+O	y86-7+O	y130-14+O	y124-12+O	y122-12+O	y106-12+O	y107-9+O	y103-11+O
y87-9+O	y82-9+O	y86-8+O	y131-12+O	y125-14+O	y124-11+O	y107-11+O	y108-10+O	y103-12+T
y87-11+E	y83-7+O	y87-8+O	y131-13+O	y127-12+O	y124-12+O	y108-9+O	y109-9+O	y104-9+O
y88-8+O	y83-8+O	y87-9+O	y132-12+O	y128-15+O	y127-12+O	y108-10+O	y109-11+O	y104-10+O
y89-9+O	y83-9+O	y87-11+E	y134-12+O	y130-10+	y128-15+O	y108-11+O	y111-10+O	y104-11+O

y89-11+	y84-7+O	y88-8+O	y134-13+O	y130-13+O	y130-10+	y109-15+O	y112-9+E	y105-11+O
y90-10+O	y84-8+O	y89-7+O	y135-12+O	y130-14+O	y130-14+O	y111-10+O	y112-11+O	y106-8+
y91-8+O	y84-9+O	y89-9+O	y136-14+O	y131-11+O	y131-11+O	y112-9+O	y112-12+O	y106-9+O
y91-9+O	y85-8+O	y89-11+	y137-12+O	y131-12+O	y131-12+O	y112-11+O	y113-11+O	y106-10+O
y91-11+O	y86-7+O	y90-10+O	y137-13+O	y132-11+O	y132-11+O	y112-12+O	y114-11+O	y106-11+O
y92-8+O	y86-8+O	y91-8+O	y137-14+O	y132-12+O	y132-12+O	y113-10+O	y114-12+O	y106-12+O
y92-9+O	y87-8+O	y91-9+O	y138-12+O	y132-14+O	y132-14+O	y113-11+O	y115-11+O	y107-11+O
y93-8+O	y87-9+O	y91-10+O	y138-13+O	y133-12+O	y134-12+O	y114-12+O	y116-9+E	y108-9+O
y94-10+O	y87-11+E	y91-11+O	y138-15+O	y134-12+O	y134-13+O	y115-11+O	y117-15+	y108-10+O
y95-8+O	y88-8+O	y92-8+O	y140-12+O	y134-13+O	y136-14+O	y118-11+O	y118-11+O	y109-11+O
y95-9+O	y88-9+O	y92-9+O	y140-13+O	y135-12+O	y137-12+O	y119-10+O	y119-11+O	y109-12+O
y96-8+O	y89-7+O	y92-12+O	y140-14+O	y136-14+O	y137-13+O	y119-11+O	y120-11+O	y111-10+O
y96-9+O	y89-8+O	y93-8+O	y141-14+O	y136-15+O	y137-14+O	y120-11+O	y120-12+O	y112-11+O
y96-10+O	y89-9+O	y93-10+O	y142-13+O	y137-12+O	y138-12+O	y120-12+O	y121-10+O	y112-12+O
y96-11+O	y89-11+	y95-8+O	y142-15+O	y137-13+O	y138-13+O	y121-10+O	y121-11+O	y113-11+O
y97-9+O	y90-7+O	y95-9+O	y143-13+O	y137-14+O	y138-15+O	y121-11+O	y123-12+O	y113-13+O
y97-10+O	y90-10+O	y96-8+O	y143-15+O	y138-13+O	y140-12+O	y121-13+O	y123-13+O	y114-12+O
y98-9+O	y91-8+O	y96-10+O	y144-13+O	y138-15+O	y140-14+O	y121-15+O	y124-14+O	y116-10+O
y98-10+O	y91-9+O	y97-9+O	y144-14+O	y139-15+O	y141-12+O	y122-10+O	y125-15+	y116-12+O
y99-8+O	y91-11+O	y97-10+O	y145-13+O	y140-12+O	y141-14+O	y123-13+O	y126-13+O	y117-15+
y99-9+O	y92-8+O	y98-9+O	y145-14+O	y140-14+O	y142-13+O	y124-11+O	y128-11+O	y118-11+O
y99-10+O	y92-9+O	y98-10+O	y145-15+O	y141-12+O	y142-15+O	y124-14+O	y129-12+O	y119-10+O
y100-8+O	y92-12+O	y99-8+O	y147-13+O	y141-14+O	y143-12+O	y125-15+E	y129-14+O	y119-11+O
y100-10+O	y93-8+O	y99-9+O	y148-12+O	y142-12+O	y143-13+O	y126-13+O	y130-12+O	y120-11+O
y101-9+O	y95-8+O	y99-10+O	y148-13+O	y142-13+O	y144-13+O	y128-11+O	y131-11+O	y120-12+O
y101-10+O	y95-9+O	y100-9+O	y148-15+O	y142-15+O	y144-14+O	y128-14+O	y131-14+O	y121-10+O
y103-10+O	y96-8+O	y100-10+O	y149-15+O	y144-13+O	y145-13+O	y129-13+O	y132-12+O	y121-11+O

y104-10+O	y96-10+O	y101-9+O	y150-14+O	y144-14+O	y145-14+O	y129-14+O	y132-13+O	y121-13+O
y105-9+O	y97-8+O	y101-10+O	y151-15+O	y145-13+O	y147-13+O	y130-12+O	y133-13+O	y122-10+O
y106-9+O	y97-9+O	y103-10+O		y145-14+O	y148-12+O	y130-14+O	y134-12+O	y123-12+O
y106-10+O	y97-10+O	y104-10+O		y145-15+O	y148-13+O	y131-11+O	y134-13+O	y123-13+O
y106-11+O	y98-9+O	y104-11+O		y147-13+O	y148-14+O	y131-12+O	y135-13+O	y124-11+O
y107-10+O	y98-10+O	y104-12+O		y147-14+O	y149-14+O	y131-13+O	y135-14+O	y124-14+O
y108-9+O	y99-8+O	y106-9+O		y148-12+O	y149-15+O	y131-14+O	y136-13+O	y125-15+E
y108-11+O	y99-9+O	y106-10+O		y148-13+O	y150-13+O	y132-12+O	y136-14+O	y127-15+O
y109-9+O	y99-10+O	y106-11+O		y148-15+O	y150-15+O	y132-13+O	y137-14+O	y128-11+E
y109-11+O	y100-8+O	y107-10+O		y149-13+O	y151-15+O	y133-12+O	y138-12+O	y129-13+O
y110-10+O	y100-10+O	y108-11+O		y149-15+O	y152-15+O	y133-13+O	y138-13+O	y130-12+O
y110-11+O	y101-10+O	y109-9+O		y151-15+O		y134-12+O	y138-14+O	y131-11+O
y111-10+O	y103-9+O	y109-10+O				y134-13+O	y139-13+O	y131-12+O
y111-11+O	y103-10+O	y109-11+O				y134-14+O	y139-15+	y131-13+O
y112-9+O	y104-9+O	y110-10+O				y135-13+O	y140-13+O	y131-14+O
y112-12+O	y104-10+O	y110-11+O				y136-14+O	y140-14+O	y131-15+O
y113-11+O	y105-9+O	y111-10+O				y137-14+O	y141-12+O	y132-12+O
y113-13+O	y105-10+O	y111-11+O				y138-12+O	y141-13+O	y132-13+O
y114-9+O	y106-9+O	y112-11+O				y138-13+O	y141-14+O	y133-13+O
y114-11+O	y106-10+O	y112-12+O				y139-13+O	y142-12+O	y134-12+O
y115-10+O	y106-11+O	y113-11+O				y139-15+O	y142-14+O	y134-13+O
y115-11+O	y107-10+O	y114-9+O				y140-13+O	y143-13+O	y135-13+O
y115-12+O	y108-9+O	y114-10+O				y140-14+O	y143-14+O	y136-13+O
y116-11+O	y108-11+O	y114-11+O				y141-12+O	y143-15+O	y136-14+O
y116-13+O	y109-9+O	y114-12+O				y141-13+O	y144-12+O	y138-12+O
y117-10+O	y109-11+O	y114-15+E				y141-14+O	y144-13+O	y138-13+O
y117-11+O	y110-9+O	y115-10+O				y141-15+O	y145-14+O	y138-14+O

y117-12+O	y110-10+O	y115-11+O				y142-12+O	y146-12+O	y138-15+O
y119-12+O	y110-11+O	y117-10+O				y142-13+O	y146-13+O	y139-13+O
y120-11+O	y111-10+O	y117-11+O				y142-14+O	y147-13+O	y139-15+O
y120-12+O	y111-11+O	y117-12+O				y142-15+O	y147-14+O	y140-13+O
y120-13+O	y112-11+O	y119-10+O				y143-13+O	y149-12+O	y140-14+O
y120-14+O	y112-12+O	y120-11+O				y143-14+O	y149-14+O	y140-15+O
y121-11+O	y113-10+O	y120-12+O				y143-15+O	y150-13+O	y141-12+O
y122-10+O	y113-11+O	y120-14+O				y144-12+O	y150-14+O	y141-13+O
y122-11+O	y114-9+O	y121-11+O				y144-13+O	y151-13+O	y141-14+O
y123-14+O	y114-10+O	y122-11+O				y144-14+O	y151-15+O	y142-12+O
y124-11+O	y114-11+O	y122-13+O				y144-15+O		y142-13+O
y125-14+O	y115-10+O	y123-11+O				y145-14+O		y142-14+O
y126-11+O	y115-11+O	y123-14+O				y146-12+O		y143-13+O
y127-13+O	y115-12+O	y124-11+O				y146-13+O		y143-14+O
y129-11+O	y116-11+O	y124-12+O				y147-13+O		y143-15+O
y129-12+O	y117-10+O	y124-13+O				y147-14+O		y144-13+O
y129-13+O	y117-11+O	y125-11+O				y147-15+O		y144-15+O
y129-14+O	y117-12+O	y125-13+O				y148-12+O		y145-14+O
y130-13+O	y119-12+O	y125-14+O				y148-14+O		y145-15+O
y131-13+O	y120-12+O	y126-13+O				y148-15+O		y146-12+O
y131-14+E	y120-14+O	y127-13+O				y149-13+O		y146-13+O
y132-12+O	y121-11+O	y128-13+O				y149-14+		y146-15+O
y132-13+O	y122-10+O	y129-12+O				y150-13+O		y147-13+O
y132-15+O	y124-11+O	y129-13+O				y150-14+O		y147-14+O
y133-12+O	y124-12+O	y129-14+O				y151-13+O		y147-15+O
y133-13+O	y124-13+O	y130-11+O				y151-15+O		y149-13+O
y133-15+O	y125-11+O	y130-13+O						y149-14+O

y134-10+O	y125-14+O	y131-13+O					y150-13+O
y134-12+O	y126-13+O	y131-14+O					y150-14+O
y134-13+O	y127-12+O	y132-12+O					y150-15+O
y134-15+O	y128-11+O	y132-13+O					y151-13+O
y135-12+O	y129-11+O	y133-12+O					y151-15+O
y135-13+O	y129-12+O	y133-13+O					y152-15+O
y136-12+O	y129-13+O	y133-14+O					
y136-13+O	y130-12+O	y134-10+O					
y136-14+O	y130-13+O	y134-12+O					
y137-13+O	y131-13+O	y134-13+O					
y137-14+O	y131-14+O	y134-15+O					
y138-10+E	y132-11+O	y135-12+O					
y138-14+O	y132-12+O	y136-12+O					
y139-12+O	y132-13+O	y136-14+O					
y139-13+O	y133-12+O	y137-12+O					
y139-14+O	y133-13+O	y137-13+O					
y139-15+O	y133-14+O	y137-14+O					
y140-12+O	y133-15+O	y138-13+O					
y141-12+O	y134-10+O	y138-14+O					
y141-13+O	y134-12+O	y139-12+O					
y141-14+O	y134-13+O	y139-13+O					
y141-15+O	y134-15+O	y139-14+O					
y142-12+O	y135-12+O	y139-15+O					
y143-12+O	y136-12+O	y140-12+O					
y143-13+O	y136-13+O	y141-14+O					
y143-14+O	y136-14+O	y141-15+O					
y143-15+O	y137-13+O	y142-12+O					

y144-13+O	y138-13+O	y143-12+O						
y144-15+O	y138-14+O	y143-13+O						
y146-13+O	y139-12+O	y143-15+O						
y148-15+O	y139-13+O	y147-13+O						
y149-15+O	y139-14+O	y147-15+O						
	y139-15+O	y148-12+O						
	y140-12+O	y148-15+O						
	y141-13+O	y149-15+O						
	y141-15+O	y151-13+O						
	y142-12+O	y152-15+O						
	y142-14+O							
	y143-12+O							
	y143-13+O							
	y143-14+O							
	y144-15+O							
	y146-12+O							
	y146-13+O							
	y147-15+O							
	y148-15+O							
	y149-13+O							

*”O”=overlapping, which means partial isotopic peaks of this ion are overlapping isotopic peaks; “E”=embedded, which means all isotopic peaks of this ion are overlapping isotopic peaks; “T”=topping, which means the theoretical most abundant isotopic peak (i.e., the relative abundance is 100%) of this ion is shared with other ion(s).

Table S3. Unique proteoforms identified from the 1st RPLC-MS/MS analysis (Dataset 1) of *E. coli* intact proteome.

retention time (min)	protein ID	sequence length	PTMs*	PTM Score	-log(P_Score)	sequence coverage	Pepetide Bond Coverage	interpreted isotopic peaks(%)	interpreted abundance(%)
87.80	SRA_ECOLI	45	null	null	172.3	100.0	18.2	64.3	84.7
88.17	YBGS_ECOLI	102	null	null	143.5	38.2	19.8	70.1	76.7
88.19	RL32_ECO57	56	null	null	322.4	100.0	29.1	53.9	47.8
88.34	RL34_ECO24	46	null	null	151.4	100.0	13.3	66.4	81.6
88.45	RL361_ECODH	38	null	null	140.5	100.0	18.9	48.1	72.3
88.82	RL33_ECOLI	54	null	null	168.9	72.2	22.6	53.3	59.3
88.95	RL33_ECOLI	54	A1Me	25	414.2	100.0	43.4	62.6	68.7
89.34	LPL_ECOL6	28	null	null	42.3	85.7	11.1	19.1	18.9
94.21	RL31_ECODH	70	K8Ac	0	180.6	50.0	14.5	55.7	69.4
96.35	RS21_ECO57	70	null	null	206.1	100.0	10.1	63.5	79.8
96.58	RL31_ECODH	70	null	null	925.2	100.0	43.5	73.7	85.1
97.58	RL28_ECO57	77	null	null	506.8	100.0	27.6	78.4	88.4
97.79	RL24_ECO57	103	null	null	1050.8	98.1	46.1	74.4	86.7
99.08	YAHO_ECOLI	70	null	null	1289.4	100.0	79.7	72.0	88.4
100.10	RS14_ECO57	100	null	null	206.8	88.0	12.1	79.1	84.6
100.47	PSPE_ECOLI	85	null	null	186.9	56.5	20.2	52.0	60.8
100.72	RS19_ECO57	91	null	null	981.3	96.7	47.8	72.0	82.7
101.06	RS20_ECO57	86	null	null	258.2	100.0	17.7	49.1	59.6
102.37	LPTN_ECO57	24	null	null	61.9	100.0	21.7	19.8	24.3
104.27	YJBJ_ECOL6	69	null	null	250.7	79.7	33.8	66.5	77.0
104.27	YJBJ_ECO57	69	null	null	1362.2	100.0	60.3	79.9	94.1
104.43	RS18_ECOLI	74	A1Ac	34	666.0	100.0	39.7	70.3	85.2
104.68	RL25_ECO27	94	null	null	265.0	48.9	14.0	62.1	68.7
104.83	RL25_ECO24	94	null	null	907.8	67.0	30.1	67.7	78.4

104.88	RL25_ECO57	94	null	null	785.1	73.4	28.0	65.2	76.8
105.01	GLRX3_ECO57	82	null	null	303.7	100.0	27.2	70.8	79.7
105.65	RS15_ECOL6	88	null	null	191.1	96.6	11.5	59.8	68.8
105.74	RS17_ECO57	83	null	null	438.7	98.8	30.5	63.1	76.1
105.89	YDFZ_ECO57	67	null	null	631.8	100.0	40.9	66.4	80.5
105.94	YQFG_ECOLI	18	null	null	20.7	61.1	11.8	4.2	4.0
106.24	RS11_ECOLI	128	A1Me	15	269.5	30.5	11.8	75.3	80.5
106.56	RL30_ECO57	58	null	null	262.5	100.0	14.0	36.7	51.2
107.00	HDEB_ECOL6	79	K64Ac	13	130.7	100.0	16.7	67.7	76.9
107.56	CSPC_ECO57	68	null	null	859.3	100.0	65.7	64.1	79.7
107.92	RL19_ECO57	114	null	null	322.8	80.7	13.3	77.8	85.2
108.00	CSPA_ECOL6	69	null	null	204.8	49.3	25.0	39.8	45.1
108.86	CSRA_ECO24	61	null	null	1019.4	100.0	48.3	72.5	88.9
110.53	HDEB_ECOL6	79	null	null	228.1	86.1	28.2	57.1	66.5
110.71	CSPE_ECO57	68	null	null	642.1	100.0	50.8	68.8	79.6
111.67	RL18_ECO24	117	null	null	1015.1	100.0	23.3	80.7	92.8
112.27	FETP_ECO57	90	null	null	318.7	44.4	28.1	56.2	69.3
112.28	GNSB_ECOLI	57	null	null	73.2	59.7	19.6	41.5	49.3
112.33	ECOT_ECO27	142	null	null	328.8	26.1	13.5	75.3	83.7
112.49	YGIW_ECO57	110	null	null	283.9	77.3	25.7	69.1	79.5
112.55	HDEA_ECOLI	89	null	null	631.3	100.0	46.6	66.9	81.1
112.94	RS16_ECO24	82	null	null	415.2	100.0	23.5	73.3	84.3
112.95	SKP_ECO57	141	null	null	244.6	23.4	16.4	78.2	88.2
112.99	RPOZ_ECOLI	90	null	null	498.5	100.0	25.8	67.1	87.2
113.34	C562_ECOLX	106	null	null	775.7	38.7	28.6	62.8	73.1
113.49	RL22_ECO24	110	null	null	631.8	69.1	23.9	80.5	90.2
113.81	CH10_ECOUT	97	null	null	766.8	96.9	32.3	72.6	86.6

113.88	RS8_ECO57	129	null	null	151.2	54.3	10.9	69.2	80.5
114.28	RL23_ECOL5	100	null	null	478.9	59.0	26.3	81.6	88.9
114.30	RL29_ECO24	63	null	null	1188.1	100.0	58.1	79.9	93.1
114.95	YGAU_ECOL6	148	null	null	136.9	39.9	10.2	12.3	20.3
115.08	YCII_ECOLI	98	null	null	408.5	76.5	22.7	55.0	71.7
115.26	YEBV_ECOL6	78	null	null	654.2	87.2	46.8	56.5	77.7
115.51	RS13_ECO57	117	null	null	340.0	63.3	13.8	79.6	85.0
116.18	YHBY_ECO57	97	null	null	270.6	51.6	18.8	62.0	73.9
116.21	GRCA_ECO45	127	null	null	484.9	79.5	17.5	79.6	90.9
116.21	GRCA_ECO55	127	null	null	711.5	80.3	20.6	82.1	92.6
116.60	IHFB_ECO27	94	null	null	183.6	41.5	12.9	63.6	76.2
116.71	YNCL_ECOLI	31	null	null	53.0	100.0	13.3	34.0	45.7
116.83	ASR_ECO45	44	null	null	99.8	100.0	14.0	38.4	43.2
116.83	YTHA_ECOLI	41	null	null	80.5	100.0	15.0	32.2	33.5
116.99	IHFB_ECO24	94	null	null	509.4	42.6	25.8	69.8	80.6
117.13	DBHA_ECOL6	90	null	null	748.9	96.7	46.1	83.8	89.3
117.29	ASR_ECO27	44	null	null	100.9	100.0	11.6	39.6	42.3
117.69	RS9_ECO57	129	null	null	494.6	89.9	12.5	89.9	94.7
117.84	YRBA_ECOL6	84	null	null	218.8	51.2	21.7	38.6	47.8
118.27	HNS_ECOLI	136	null	null	569.6	76.5	17.0	82.7	94.2
118.48	GNSA_ECOL6	57	null	null	65.4	45.6	16.1	34.8	44.9
118.65	IHFA_ECOLI	98	null	null	78.4	56.1	10.3	61.1	74.2
119.21	IPYR_ECOL6	175	null	null	467.4	39.4	16.7	84.9	90.9
119.25	PTHP_ECOL6	85	null	null	685.0	100.0	48.8	70.6	84.6
119.34	YCIN_ECO57	83	null	null	213.2	71.1	17.1	53.3	63.3
119.54	RS5_ECOLI	166	A1Ac	27	437.0	32.5	16.4	91.1	95.2
119.69	RL11_ECOUT	141	A1tMeK3tMeK39tMe	0, 0, 0	386.2	63.1	19.3	66.7	78.3

119.86	YODD_ECOLI	75	null	null	274.4	97.3	25.7	33.9	45.2
120.74	RS7_ECO57	155	null	null	413.2	38.1	17.5	90.6	92.8
121.13	PPTA_ECO24	74	null	null	638.3	100.0	50.7	61.2	76.4
121.60	HPF_ECO57	95	null	null	341.0	100.0	27.7	71.2	83.4
124.41	BCCP_ECOL6	156	K122Bi	24	271.1	35.9	15.5	70.6	79.3
124.98	YCCJ_ECOLI	74	null	null	1232.4	100.0	71.2	71.8	90.1
125.92	ACP_ECOLI	77	S36PaP	54	719.6	96.1	71.1	57.7	73.5
126.18	ACP_ECO24	78	S37PaP	41	510.5	87.2	53.3	46.9	64.4
126.27	DBHB_ECO57	90	null	null	804.3	100.0	39.3	72.8	88.6
128.39	NDK_ECOLI	142	null	null	577.3	97.2	31.2	76.5	86.2
129.57	LPF_ECOLI	15	null	null	18.6	73.3	14.3	4.8	6.5
129.70	RBSD_ECO24	139	null	null	189.2	43.2	10.1	74.4	84.7
129.79	YHEU_ECO27	72	null	null	109.8	43.1	11.3	52.3	67.6
130.75	RIDA_ECOLI	127	null	null	617.8	44.1	24.6	72.1	86.7
131.30	YOEI_ECOLI	20	null	null	17.0	70.0	10.5	6.7	8.1
133.40	ASR_ECO57	44	null	null	44.8	61.4	11.6	33.1	41.2
134.75	USPA_ECO57	143	null	null	217.6	44.8	13.4	72.5	82.5
138.30	LPT_ECO57	27	null	null	24.6	66.7	11.5	19.3	26.3
138.34	YMIB_ECOLI	34	null	null	25.1	64.7	12.1	31.6	38.3
138.75	IROK_ECOLI	21	null	null	20.6	76.2	10.0	12.3	17.3
140.02	DPS_ECOLI	166	null	null	249.3	35.5	11.5	81.0	88.8
150.19	RL7_ECOLI	120	null	null	554.2	87.5	31.9	61.5	74.8
150.22	RL7_ECOLI	120	K81Me	4	535.0	86.7	33.6	62.0	75.5
153.74	RL7_ECOLI	120	S1Ac	6	686.3	80.0	32.8	62.9	78.0
153.74	RL7_ECOLI	120	S1AcK81Me	21, 3	536.6	77.5	30.3	62.0	77.4

* Ac=acetylation, Bi=biotinyl, Me=mono-methylation, null=no modification, PaP= O-(pantetheine 4'-phosphoryl), tMe=trimethylation,

Table S4. Unique proteoforms identified from the 2nd RPLC-MS/MS analysis (Dataset 2) of *E. coli* intact proteome.

retention time (min)	protein ID	sequence length	PTMs*	PTM Score	-log(P_Score)	sequence coverage	Pepetide Bond Coverage	interpreted isotopic peaks(%)	interpreted abundance(%)
87.80	SRA_ECOLI	45	null	null	345.3	100.0	27.3	71.7	89.9
88.11	YBGS_ECOLI	102	null	null	256.9	45.1	27.7	75.4	82.3
88.13	RL32_ECO57	56	null	null	567.7	100.0	45.5	64.2	78.0
88.19	RL361_ECODH	38	null	null	248.8	100.0	32.4	58.2	82.1
88.22	RL34_ECO24	46	null	null	121.0	100.0	11.1	65.9	79.4
88.81	RL33_ECOLI	54	A1Me	14	505.9	100.0	50.9	65.8	73.2
88.81	RL33_ECOLI	54	null	null	332.9	100.0	34.0	53.2	60.3
91.40	LPL_ECOL6	28	null	null	36.6	82.1	14.8	21.2	22.0
94.04	RL31_ECODH	70	K8Ac	0	160.8	50.0	11.6	51.9	66.4
96.40	RS21_ECO57	70	null	null	209.7	100.0	11.6	70.1	83.0
96.63	RL31_ECODH	70	null	null	794.8	100.0	44.9	72.1	83.4
98.20	RL24_ECOL6	103	null	null	569.9	77.7	25.5	72.0	78.2
98.24	RL24_ECO57	103	null	null	1052.7	100.0	42.2	75.3	87.9
98.27	RL28_ECO57	77	null	null	581.5	100.0	27.6	83.1	91.9
99.45	YAHO_ECOLI	70	null	null	1203.4	100.0	78.3	76.2	89.5
100.44	RS14_ECO57	100	null	null	216.4	100.0	11.1	79.7	85.7
100.75	PSPE_ECOLI	85	null	null	228.6	51.8	25.0	43.8	51.3
101.29	RS19_ECO57	91	null	null	955.0	96.7	47.8	73.7	81.9
101.99	RS20_ECO57	86	null	null	241.7	54.7	11.8	72.1	80.9
102.30	LPTN_ECO57	24	null	null	28.4	70.8	13.0	15.4	19.7
103.98	YJBJ_ECO57	69	null	null	1378.6	100.0	61.8	80.4	93.4
103.98	YJBJ_ECOL6	69	null	null	296.5	95.7	38.2	67.8	81.7
104.24	YNFD_ECOLI	80	null	null	198.6	100.0	10.1	23.5	32.0
104.57	RS18_ECOLI	74	A1Ac	28	611.1	94.6	38.4	58.2	72.0

104.98	GLRX3_ECO57	82	null	null	168.7	87.8	18.5	57.9	65.2
105.14	RL25_ECO57	94	null	null	823.9	66.0	26.9	67.3	78.6
105.14	RL25_ECO24	94	null	null	823.7	66.0	26.9	66.4	77.4
105.68	YDFZ_ECO57	67	null	null	613.6	100.0	40.9	66.4	81.6
105.72	RS17_ECO57	83	null	null	352.0	97.6	23.2	68.8	80.4
105.85	RS11_ECOLI	128	A1Me	15	296.3	30.5	11.8	73.9	80.0
106.04	RS15_ECOL6	88	null	null	189.3	93.2	10.3	62.0	74.6
106.25	RL30_ECO57	58	null	null	121.2	100.0	12.3	33.1	43.6
106.59	HDEB_ECOL6	79	K64Ac	13	85.7	41.8	16.7	67.8	80.4
106.89	CSPC_ECO57	68	null	null	864.9	100.0	59.7	64.9	80.5
107.78	RL19_ECO57	114	null	null	307.8	41.2	12.4	81.2	86.7
107.82	CSPA_ECOL6	69	null	null	71.5	29.0	11.8	40.0	43.2
108.55	CSRA_ECO24	61	null	null	1047.4	100.0	53.3	76.2	91.9
110.16	YJEI_ECOLI	95	null	null	96.6	40.0	10.6	50.0	56.0
110.35	CSPE_ECO57	68	null	null	760.4	100.0	55.2	65.8	83.4
110.75	HDEB_ECOL6	79	null	null	255.3	86.1	34.6	62.8	72.6
111.59	RL18_ECO24	117	null	null	915.2	100.0	25.0	80.3	91.9
112.13	GNSB_ECOLI	57	null	null	61.3	40.4	19.6	39.5	48.2
112.22	ECOT_ECO27	142	null	null	439.4	28.2	18.4	76.9	85.3
112.43	FETP_ECO57	90	null	null	439.8	44.4	33.7	57.7	71.9
112.66	HDEA_ECOLI	89	null	null	713.4	70.8	45.5	65.9	80.2
112.73	FETP_ECOL6	90	null	null	179.6	31.1	19.1	51.3	58.9
112.82	YGIW_ECO57	110	null	null	325.9	49.1	29.4	70.5	82.5
112.96	RS16_ECO24	82	null	null	613.9	100.0	19.8	72.6	88.1
113.03	RPOZ_ECOLI	90	null	null	520.6	100.0	30.3	67.8	86.7
113.04	SKP_ECO57	141	null	null	249.3	26.2	15.7	78.2	88.9
113.07	C562_ECOL6	106	null	null	671.7	38.7	25.7	65.9	76.0

113.07	C562_ECO57	106	null	null	124.2	84.0	10.5	20.1	35.9
113.10	C562_ECOLX	106	null	null	697.5	41.5	28.6	66.6	79.1
113.60	CH10_ECOUT	97	null	null	595.3	80.4	26.0	73.5	87.0
113.79	RL22_ECO24	110	null	null	529.1	69.1	22.9	81.2	90.9
113.81	RS8_ECO57	129	null	null	180.8	87.6	12.5	72.4	82.0
114.25	RL29_ECO24	63	null	null	1330.3	100.0	61.3	75.2	91.7
114.53	RL23_ECOL5	100	null	null	469.2	55.0	24.2	82.0	89.9
115.02	YCII_ECOLI	98	null	null	478.6	77.6	25.8	56.1	66.6
115.11	YGAU_ECOL6	148	null	null	151.7	23.7	10.9	9.1	13.1
115.28	YEBV_ECOL6	78	null	null	625.4	85.9	45.5	62.7	80.3
115.49	RS13_ECO57	117	null	null	246.2	65.0	13.8	77.1	83.9
116.02	GRCA_ECO55	127	null	null	716.3	83.5	23.0	80.7	91.7
116.29	GRCA_ECO45	127	null	null	718.5	100.0	24.6	76.8	89.3
116.50	YHBY_ECO57	97	null	null	164.4	51.6	13.5	54.0	61.9
116.72	IHFB_ECO24	94	null	null	531.2	45.7	26.9	79.3	90.5
116.77	IHFB_ECO27	94	null	null	419.0	43.6	21.5	78.4	90.3
116.90	ASR_ECO45	44	null	null	51.2	100.0	11.6	19.6	24.3
117.05	DBHA_ECOL6	90	null	null	874.8	100.0	48.3	80.3	89.3
117.08	ASR_ECOLU	44	null	null	72.9	100.0	11.6	21.3	26.9
117.08	ASR_ECO57	44	null	null	72.9	100.0	11.6	21.8	27.4
117.21	YTHA_ECOLI	41	null	null	97.6	100.0	10.0	34.6	35.0
117.24	ASR_ECO27	44	null	null	128.3	100.0	16.3	39.0	41.0
117.25	YPDK_ECOLI	23	null	null	45.2	65.2	18.2	5.6	7.6
117.65	RS9_ECO57	129	null	null	298.9	91.5	10.9	86.5	93.2
117.71	GLCG_ECOL6	134	null	null	149.9	32.1	17.3	67.4	76.5
118.02	YRBA_ECOL6	84	null	null	272.0	50.0	22.9	45.7	56.7
118.63	HNS_ECOLI	136	null	null	637.6	58.1	16.3	84.5	95.0

118.77	GNSA_ECOL6	57	null	null	66.1	59.7	16.1	38.0	50.7
118.99	IHFA_ECOLI	98	null	null	94.4	56.1	10.3	65.6	78.3
119.38	PTHP_ECOL6	85	null	null	686.9	100.0	48.8	71.5	86.1
119.45	IPYR_ECOL6	175	null	null	337.4	25.7	16.1	83.0	89.5
119.65	YCIN_ECO57	83	null	null	350.0	72.3	24.4	55.6	68.8
119.76	RS5_ECOLI	166	A1Ac	26	492.3	34.9	15.8	88.9	93.0
119.94	RL11_ECOUT	141	A1tMeK3tMeK39tMe	0, 0, 0	420.1	63.1	20.0	64.3	78.0
119.99	YODD_ECOLI	75	null	null	126.3	66.7	16.2	35.0	41.2
120.70	RS7_ECO57	155	null	null	382.0	38.1	16.9	91.2	93.6
121.22	PPTA_ECO24	74	null	null	888.6	100.0	57.5	68.2	82.7
121.85	HPF_ECO57	95	null	null	406.0	100.0	28.7	73.6	86.2
124.37	BCCP_ECOL6	156	K122Bi	21	225.1	35.9	13.6	70.7	80.4
125.08	YCCJ_ECOLI	74	null	null	1397.0	100.0	78.1	80.9	93.7
126.18	ACP_ECOLI	77	S36PaP	51	785.1	87.0	67.1	52.0	71.6
126.46	DBHB_ECO57	90	null	null	893.6	96.7	41.6	78.1	88.0
126.46	RNFH_ECO7I	96	null	null	137.9	40.6	10.5	21.1	34.0
126.50	ACP_ECO24	78	S37PaP	42	568.2	88.5	54.6	47.9	65.4
128.84	NDK_ECOLI	142	null	null	520.3	97.2	27.7	76.8	87.3
130.07	RBSD_ECO24	139	null	null	202.6	31.7	10.9	67.0	77.5
130.14	YHEU_ECO27	72	null	null	145.4	45.8	14.1	44.6	56.9
131.22	RIDA_ECOLI	127	null	null	686.0	47.2	26.2	73.3	86.9
134.58	USPA_ECO57	143	null	null	261.2	44.8	15.5	73.2	84.2
137.72	IROK_ECOLI	21	null	null	23.1	76.2	10.0	16.1	20.1
139.60	DPS_ECOLI	166	null	null	288.3	29.5	10.9	82.4	89.1
140.44	YFCL_ECO57	92	null	null	115.7	52.2	12.1	33.0	39.7
149.83	RL7_ECOLI	120	null	null	702.2	93.3	36.1	65.2	77.3
149.83	RL7_ECOLI	120	K81Me	4	629.1	92.5	36.1	66.9	81.1

153.19	RL7_ECOLI	120	S1AcK81Me	23, 4	554.1	77.5	31.9	60.6	75.2
153.49	RL7_ECOLI	120	S1Ac	4	689.5	82.5	32.8	61.0	77.1

* Ac=acetylation, Bi=biotinyl, Me=mono-methylation, null=no modification, PaP= O-(pantetheine 4'-phosphoryl), tMe=trimethylation,

Table S5. Unique proteoforms identified from the 3rd RPLC-MS/MS analysis (Dataset 3) of *E. coli* intact proteome.

retention time (min)	protein ID	sequence length	PTMs*	PTM Score	-log(P_Score)	sequence coverage	Pepetide Bond Coverage	interpreted isotopic peaks(%)	interpreted abundance(%)
87.97	SRA_ECOLI	45	null	null	830.2	100.0	56.8	77.1	92.7
88.15	YBGS_ECOLI	102	null	null	305.0	52.9	30.7	75.3	82.5
88.34	RL32_ECO57	56	null	null	464.8	98.2	40.0	59.9	71.0
88.45	RL361_ECODH	38	null	null	232.5	100.0	27.0	51.3	68.8
88.46	RL34_ECO24	46	null	null	116.0	100.0	11.1	60.6	78.6
88.92	RL33_ECOLI	54	null	null	294.4	100.0	32.1	51.8	60.0
89.33	RL33_ECOLI	54	A1Me	31	542.8	100.0	50.9	61.8	73.4
92.52	LPL_ECOL6	28	null	null	33.8	85.7	14.8	20.0	21.3
95.54	RL31_ECODH	70	K8Ac	0	153.7	50.0	14.5	60.1	72.5
96.60	RL31_ECODH	70	null	null	713.3	100.0	40.6	68.0	82.0
97.89	RS21_ECO57	70	null	null	157.1	98.6	10.1	65.9	80.7
98.10	RL28_ECO57	77	null	null	448.6	100.0	27.6	82.1	91.2
98.27	RL24_ECO57	103	null	null	1057.7	97.1	41.2	73.0	87.2
98.75	RL24_ECOL6	103	null	null	494.6	77.7	26.5	68.8	78.6
99.18	YAHO_ECOLI	70	null	null	1225.5	100.0	76.8	77.1	90.4
99.51	REPL1_ECOLI	24	null	null	19.1	100.0	13.0	16.5	17.3
100.37	RS14_ECO57	100	null	null	219.4	44.0	12.1	81.2	87.5
100.67	PSPE_ECOLI	85	null	null	317.1	52.9	28.6	46.6	55.0
101.00	RS19_ECO57	91	null	null	944.1	86.8	45.6	71.6	81.4
101.31	RS20_ECO57	86	null	null	162.9	60.5	10.6	23.9	34.6
103.72	YJBJ_ECO57	69	null	null	1349.2	100.0	60.3	79.6	93.9
104.01	YJBJ_ECOL6	69	null	null	263.9	88.4	33.8	64.9	78.9
104.42	RS18_ECOLI	74	A1Ac	31	738.7	100.0	41.1	63.8	78.3
104.48	RS18_ECOLI	74	null	null	208.1	83.8	17.8	50.1	57.4

104.65	RL25_ECO24	94	null	null	799.7	72.3	28.0	66.6	78.0
104.91	GLRX3_ECO57	82	null	null	156.9	100.0	18.5	70.9	76.0
105.31	RL25_ECO57	94	null	null	747.4	67.0	29.0	68.4	79.8
105.55	RS17_ECO57	83	null	null	421.4	98.8	26.8	64.0	75.0
105.59	YDFZ_ECO57	67	null	null	767.8	100.0	37.9	71.6	87.7
105.79	RS11_ECOLI	128	A1Me	14	272.3	30.5	11.0	75.4	82.0
107.04	HDEB_ECOL6	79	K64Ac	2	80.0	34.2	18.0	72.3	82.5
107.21	CSPC_ECO57	68	null	null	873.2	97.1	62.7	65.2	78.8
107.95	RL19_ECO57	114	null	null	363.8	100.0	13.3	71.8	83.5
108.69	YMJD_ECOLI	21	null	null	10.5	81.0	10.0	11.0	14.8
108.91	CSRA_ECO24	61	null	null	1053.0	100.0	53.3	77.3	92.3
109.80	LPFS_ECO57	28	null	null	40.2	100.0	14.8	20.5	23.8
110.48	CSPE_ECO57	68	null	null	841.0	100.0	56.7	67.0	83.6
110.51	YJEI_ECOLI	95	null	null	85.2	39.0	10.6	53.9	59.7
110.76	IBSD_ECOLI	19	null	null	11.9	36.8	11.1	1.3	1.1
110.77	HDEB_ECOL6	79	null	null	291.0	100.0	34.6	63.8	75.8
111.74	RL18_ECO24	117	null	null	783.4	100.0	24.1	80.4	92.9
112.17	GNSB_ECOLI	57	null	null	74.6	40.4	21.4	39.9	48.2
112.35	FETP_ECO57	90	null	null	435.7	43.3	33.7	56.5	67.8
112.35	ECOT_ECO27	142	null	null	343.8	27.5	16.3	75.1	83.7
112.37	YGIW_ECO57	110	null	null	337.7	64.6	31.2	73.5	84.1
112.65	HDEA_ECOLI	89	null	null	674.0	66.3	43.2	66.5	80.6
112.98	C562_ECOLX	106	null	null	823.8	39.6	28.6	64.7	75.0
113.03	RPOZ_ECOLI	90	null	null	482.7	100.0	30.3	67.5	87.3
113.13	C562_ECOL6	106	null	null	726.5	41.5	27.6	68.5	78.0
113.21	RS16_ECO24	82	null	null	409.4	100.0	25.9	76.3	87.5
113.26	SKP_ECO57	141	null	null	198.0	27.0	15.0	77.2	88.5

113.58	CH10_ECOUT	97	null	null	975.8	92.8	36.5	75.8	88.9
113.69	RL22_ECO24	110	null	null	618.9	75.5	23.9	81.3	91.7
113.99	RS8_ECO57	129	null	null	124.5	41.9	10.2	72.7	82.1
114.32	SAFA_ECO57	65	null	null	213.9	100.0	12.5	58.2	67.2
114.47	RL23_ECOL5	100	null	null	372.6	55.0	22.2	78.8	86.6
114.50	RL29_ECO24	63	null	null	1204.5	100.0	62.9	78.5	92.8
114.98	YEBV_ECOL6	78	null	null	726.2	87.2	54.6	60.2	76.1
115.00	YCII_ECOLI	98	null	null	459.1	96.9	34.0	67.3	80.7
115.09	YGAU_ECOL6	148	null	null	239.6	33.8	14.3	78.1	87.9
115.68	RS13_ECO57	117	null	null	293.9	63.3	14.7	80.0	84.7
116.25	GRCA_ECO55	127	null	null	730.1	78.0	23.0	82.5	92.1
116.33	GRCA_ECO45	127	null	null	582.2	78.0	20.6	80.0	91.0
116.70	YHBY_ECO57	97	null	null	162.2	51.6	12.5	56.0	65.3
116.83	IHFB_ECO27	94	null	null	456.9	42.6	19.4	78.9	88.3
116.86	IHFB_ECO24	94	null	null	553.0	91.5	26.9	77.0	89.4
117.11	ASR_ECO27	44	null	null	106.6	100.0	18.6	40.4	42.6
117.16	YTHA_ECOLI	41	null	null	78.4	100.0	10.0	34.8	35.6
117.23	DBHA_ECOL6	90	null	null	797.5	94.4	43.8	82.5	88.4
117.58	GLCG_ECOL6	134	null	null	159.6	32.8	12.8	69.2	72.4
117.66	IBSC_ECOLI	19	null	null	11.3	42.1	11.1	1.3	2.5
117.70	RS9_ECO57	129	null	null	554.6	89.9	13.3	90.0	95.0
117.94	PRTL_ECOLI	29	null	null	23.5	44.8	10.7	15.6	21.2
117.97	YRBA_ECOL6	84	null	null	261.9	50.0	20.5	42.3	52.7
118.26	HNS_ECOLI	136	null	null	444.4	68.4	13.3	85.6	95.3
119.33	IPYR_ECOL6	175	null	null	428.9	37.7	14.9	85.5	91.1
119.35	PTHP_ECOL6	85	null	null	780.8	100.0	52.4	73.8	87.0
119.53	YCIN_ECO57	83	null	null	498.7	91.6	32.9	58.4	73.2

119.60	RS5_ECOLI	166	A1Ac	24	405.3	32.5	14.6	90.1	94.6
119.81	YODD_ECOLI	75	null	null	65.8	44.0	13.5	30.4	31.3
119.93	RL11_ECOUT	141	A1tMeK3tMeK39tMe	0, 0, 0	404.3	63.1	19.3	64.6	76.7
120.93	RS7_ECO57	155	null	null	358.8	36.8	17.5	89.0	92.8
121.11	PPTA_ECO24	74	null	null	957.8	100.0	63.0	69.0	82.6
121.75	HPF_ECO57	95	null	null	406.2	100.0	28.7	72.8	85.4
124.55	BCCP_ECOL6	156	K122Bi	21	201.2	35.9	13.6	71.4	79.8
125.13	YCCJ_ECOLI	74	null	null	1176.2	100.0	74.0	79.1	91.6
126.41	DBHB_ECO57	90	null	null	979.8	96.7	42.7	78.6	88.1
126.44	ACP_ECOLI	77	S36PaP	55	712.6	96.1	72.4	53.2	70.2
126.53	ACP_ECO24	78	S37PaP	37	471.4	82.1	48.1	47.3	64.4
126.86	LPT_ECO55	21	null	null	22.7	61.9	15.0	13.5	16.1
127.15	PTGA_ECOL6	168	null	null	215.1	23.8	11.4	75.9	83.3
128.22	NDK_ECOLI	142	null	null	440.8	78.9	26.2	79.2	87.7
129.47	DSRB_ECO27	62	null	null	182.3	88.7	21.3	47.8	65.8
130.51	RIDA_ECOLI	127	null	null	644.0	44.1	27.8	73.4	87.2
132.18	LPF_ECOLI	15	null	null	21.7	73.3	21.4	5.7	8.2
134.51	USPA_ECO57	143	null	null	223.1	40.6	14.1	74.5	85.2
140.09	DPS_ECOLI	166	null	null	249.6	22.3	11.5	81.3	90.7
140.28	YFCL_ECO57	92	null	null	84.2	48.9	11.0	31.6	37.2
140.79	ECNA_ECOLI	41	null	null	18.9	36.6	10.0	18.3	20.5
141.41	RISB_ECO24	156	null	null	149.4	64.1	11.0	76.7	87.1
142.23	LPW_ECOL6	14	null	null	13.1	100.0	15.4	6.2	9.5
149.22	RL7_ECOLI	120	null	null	663.4	92.5	34.5	66.7	79.2
149.22	RL7_ECOLI	120	K81Me	3	576.9	92.5	34.5	66.3	79.3
153.44	RL7_ECOLI	120	S1Ac	3	632.3	79.2	32.8	66.1	80.3
153.50	RL7_ECOLI	120	S1AcK81Me	24, 4	559.5	82.5	35.3	61.4	76.8

* Ac=acetylation, Bi=biotinyl, Me=mono-methylation, null=no modification, PaP= O-(pantetheine 4'-phosphoryl), tMe=trimethylation,

Table S6. Resolving of continuously overlapping y10-1+, y20-2+, y72-7+ and y144-14+ using OIE_CARE

		OIPs		y144-14+	y10-1+	y20-2+	y72-7+	
		Exp. m/z	Exp. Abun. (obs.)	Exp. Abun.(ideal)	Exp. Abun. (ideal)	Exp. Abun. (ideal)	Exp. Abun. (ideal)	Deviation
		1140.619995	202815.59375	96820.019972	202587.872211	N/A	N/A	-0.32
		1142.617676	480992.31250	N/A	46615.469396	416521.895787	66542.651159	-0.09
		1143.622437	322338.03125	N/A	12256.566269	134245.007012	24352.241570	0.89
		1144.620605	152118.46875	N/A	2593.124764	48.341919	48.341919	55.55
		1145.624756	128238.96875	N/A	465.952106	999.652550	N/A	86.50
y144-14+								
Theo.m/z	Theo.abun.	Exp.m/z	Exp.abun.	Exp.rel.abun.	IPMD	IPAD	IPAD#	
1139.617780	0.08	1139.626221	140508.406250	55.64	7	56		
1139.689414	0.68	N/A	N/A					
1139.761045	3.01	1139.763184	62628.578125	24.80	2	22		
1139.832674	9.04	1139.835449	40559.347656	16.06	2	7		
1139.904300	20.61	1139.909790	83208.718750	32.95	5	12		
1139.975923	37.98	1139.980225	140659.203125	55.70	4	18		
1140.047545	58.92	1140.051514	140489.421875	55.63	3	-3		
1140.119165	79.14	1140.123657	212096.796875	83.99	4	5		
1140.190783	93.92	1140.192749	185808.468750	73.58	2	-20		
1140.262399	100.00	1140.265747	252530.046875	100.00	3	0		
1140.334014	96.70	1140.334351	180920.234375	71.64	0	-25		
1140.405627	85.76	1140.410278	239114.640625	94.69	4	9		
1140.477239	70.32	1140.481201	140664.109375	55.70	3	-15		
1140.548850	53.66	1140.549072	124679.992188	49.37	0	-4		
1140.620459	38.34	1140.619995	65584.810388	25.97	0	-12	42	
1140.692068	25.76	1140.699951	44760.613281	17.72	7	-8		
1140.763676	16.35	N/A	N/A					

1140.835283	9.84	1140.841919	46094.058594	18.25	6	8		
1140.906889	5.63	1140.906372	44991.183594	17.82	0	12		
1140.978494	3.07	N/A	N/A					
1141.050099	1.60	1141.036011	34251.699219	13.56	-12	12		
1141.121704	0.80	1141.121460	46430.578125	18.39	0	18		
1141.193300	0.38	1141.180298	53875.929688	21.33	-11	21		
1141.264893	0.18	1141.249878	28408.728516	11.25	-13	11		
1141.336474	0.08	1141.328003	26937.857422	10.67	-7	11		
1141.408050	0.03	N/A	N/A					
1141.479614	0.01	1141.485107	22134.361328	8.77	5	9		
1141.551174	0.01	1141.534546	44958.507813	17.80	-15	18		
y10-1+								
Theo.m/z	Theo.abun.	Exp.m/z	Exp.abun.	Exp.rel.abun.	IPMD	IPAD	IPAD#	
1140.604785	100.00	1140.619995	137230.7834	67.74	13	-32	0	
1141.607714	63.64	1141.61377	128926.9219	63.64	5	0		
1142.610419	23.01	1142.617676	42330.61798	20.89	6	-2	214	
1143.613035	6.05	1143.622437	23123.61269	11.41	8	5	153	
1144.615593	1.28	1144.620605	146650.6458	72.39	4	71	74	
1145.618114	0.23	1145.624756	40770.3519	20.12	6	20	63	
1146.620138	0.03	N/A	N/A					
y20-2+								
Theo.m/z	Theo.abun.	Exp.m/z	Exp.abun.	Exp.rel.abun.	IPMD	IPAD	IPAD#	
1142.110507	79.01	1142.118896	234434.9531	56.28	7	-23		
1142.611961	100.00	1142.617676	480992.3125	115.48	5	15	15	
1143.113357	67.65	1143.120117	281777.0625	67.65	6	0		
1143.614714	32.23	1143.622437	253270.735	60.81	7	29	45	

1144.116044	12.07	1144.119019	91763.52344	22.03	3	10		
1144.617353	3.76	1144.620605	2733.911495	0.66	3	-3	33	
1145.118650	1.01	1145.125	53927.35938	12.95	6	12		
1145.619949	0.24	1145.624756	87468.61685	21.00	4	21	31	
1146.121013	0.05	N/A	N/A					
1146.622345	0.01	N/A	N/A					
y72-7+								
Theo.m/z	Theo.abun.	Exp.m/z	Exp.abun.	Exp.rel.abun.	IPMD	IPAD	IPAD#	
1142.321240	5.52	1142.335938	48493.79297	40.13	13	35		
1142.464508	24.36	1142.471436	42773.50391	35.39	6	11		
1142.607764	55.06	1142.617676	60426.11138	50.00	9	-5	343	
1142.751011	84.81	1142.765625	111320.6875	92.11	13	7		
1142.894250	100.00	1142.910522	120854.7969	100.00	14	0		
1143.037482	96.16	1143.041626	156779.8125	129.73	4	34		
1143.180708	78.46	1143.184692	41471.28125	34.31	3	-44		
1143.323928	55.82	1143.329102	99924.15625	82.68	5	27		
1143.467145	35.30	1143.474365	29248.83984	24.20	6	-11		
1143.610358	20.15	1143.622437	45943.68356	38.02	11	18	247	
1143.753567	10.50	N/A	N/A					
1143.896775	5.03	N/A	N/A					
1144.039981	2.24	1144.053345	23893.9668	19.77	12	18		
1144.183186	0.93	1144.190186	162360.3594	134.34	6	133		
1144.326390	0.36	1144.324585	27627.71484	22.86	-2	23		
1144.469559	0.13	1144.476318	60021.69141	49.66	6	50		
1144.612736	0.04	1144.620605	2733.911495	2.26	7	2	126	
1144.755865	0.01	1144.770996	54980.99219	45.49	13	45		

#The equivalent IPAD values without OIE_CARE exp = experimental, theo = theoretical, abun = abundance

Table S7. PTMs annotated in the customized ProteinGoggle database for the search of *E. coli* proteoforms in this study. TG = target, PP = position of the modification in the polypeptide, CF = correction formula, PA = position of the modification on the amino acid.

ID	TG	Long Name	Short Name	PP	CF	PA
N-acetylalanine	Alanine	acetyl	Ac	N-terminal	C2 H2 O1	Amino acid backbone
N2-acetylarginine	Arginine	acetyl	Ac	N-terminal	C2 H2 O1	Amino acid backbone
N-acetylaspartate	Aspartate	acetyl	Ac	N-terminal	C2 H2 O1	Amino acid backbone
N-acetylcysteine	Cysteine	acetyl	Ac	N-terminal	C2 H2 O1	Amino acid backbone
N-acetylglutamate	Glutamate	acetyl	Ac	N-terminal	C2 H2 O1	Amino acid backbone
N-acetylglycine	Glycine	acetyl	Ac	N-terminal	C2 H2 O1	Amino acid backbone
N-acetylisoleucine	Isoleucine	acetyl	Ac	N-terminal	C2 H2 O1	Amino acid backbone
N6-acetyllysine	Lysine	acetyl	Ac	Anywhere	C2 H2 O1	Amino acid side chain
N-acetylated lysine	Lysine	acetyl	Ac	N-terminal	C2 H2 O1	Amino acid backbone
N-acetylmethionine	Methionine	acetyl	Ac	N-terminal	C2 H2 O1	Amino acid backbone
N-acetylproline	Proline	acetyl	Ac	N-terminal	C2 H2 O1	Amino acid backbone
O-acetylserine	Serine	acetyl	Ac	Anywhere	C2 H2 O1	Amino acid side chain
N-acetylserine	Serine	acetyl	Ac	N-terminal	C2 H2 O1	Amino acid backbone
O-acetylthreonine	Threonine	acetyl	Ac	Anywhere	C2 H2 O1	Amino acid side chain
N-acetylthreonine	Threonine	acetyl	Ac	N-terminal	C2 H2 O1	Amino acid backbone
N-acetyltyrosine	Tyrosine	acetyl	Ac	N-terminal	C2 H2 O1	Amino acid backbone
N-acetylvaline	Valine	acetyl	Ac	N-terminal	C2 H2 O1	Amino acid backbone
Glycyl adenylate	Glycine	adenyl	Ad	C-terminal	C10 H12 N5 O6 P1	Amino acid backbone
O-AMP-threonine	Threonine	adenyl	Ad	Anywhere	C10 H12 N5 O6 P1	Amino acid side chain
O-AMP-tyrosine	Tyrosine	adenyl	Ad	Anywhere	C10 H12 N5 O6 P1	Amino acid side chain
Aspartic acid 1-[(3-aminopropyl)(5'-adenosyl)phosphono]amide	Asparagine	aminopropyladenosylphosphono	PrA	C-terminal	C13 H19 N6 O6 P1	Amino acid backbone
N6-biotinyllysine	Lysine	biotinyl	Bi	Anywhere	C16 H26 N4 O3 S1	Amino acid side chain
3,4-dihydroxyarginine	Arginine	dihydroxy	dH	Anywhere	O2	Amino acid side chain

Cysteine sulfinic acid (-SO2H)	Cysteine	dihydroxy	dH	Anywhere	O2	Amino acid side chain
(3R,4R)-4,5-dihydroxyisoleucine	Isoleucine	dihydroxy	dH	Anywhere	O2	Amino acid side chain
(3S,4R)-3,4-dihydroxyisoleucine	Isoleucine	dihydroxy	dH	Anywhere	O2	Amino acid side chain
(4R)-4,5-dihydroxyleucine	Leucine	dihydroxy	dH	Anywhere	O2	Amino acid side chain
4,5-dihydroxylysine	Lysine	dihydroxy	dH	Anywhere	O2	Amino acid side chain
Methionine sulfone	Methionine	dihydroxy	dH	Anywhere	O2	Amino acid side chain
(3R,4R)-3,4-dihydroxyproline	Proline	dihydroxy	dH	Anywhere	O2	Amino acid side chain
(3R,4S)-3,4-dihydroxyproline	Proline	dihydroxy	dH	Anywhere	O2	Amino acid side chain
3,4-dihydroxyproline	Proline	dihydroxy	dH	Anywhere	O2	Amino acid side chain
N-formylglycine	Glycine	formyl	Fo	N-terminal	C1 O1	Amino acid backbone
N6-formyllysine	Lysine	formyl	Fo	Anywhere	C1 O1	Amino acid side chain
N-formylmethionine	Methionine	formyl	Fo	N-terminal	C1 O1	Amino acid backbone
(3R)-3-hydroxyarginine	Arginine	hydroxy	Hyd	Anywhere	O1	Amino acid side chain
4-hydroxyarginine	Arginine	hydroxy	Hyd	Anywhere	O1	Amino acid side chain
(3R)-3-hydroxyasparagine	Asparagine	hydroxy	Hyd	Anywhere	O1	Amino acid side chain
(3S)-3-hydroxyasparagine	Asparagine	hydroxy	Hyd	Anywhere	O1	Amino acid side chain
3-hydroxyasparagine	Asparagine	hydroxy	Hyd	Anywhere	O1	Amino acid side chain
(3R)-3-hydroxyaspartate	Aspartate	hydroxy	Hyd	Anywhere	O1	Amino acid side chain
(3S)-3-hydroxyaspartate	Aspartate	hydroxy	Hyd	Anywhere	O1	Amino acid side chain
3-hydroxyaspartate	Aspartate	hydroxy	Hyd	Anywhere	O1	Amino acid side chain
Cysteine sulfenic acid (-SOH)	Cysteine	hydroxy	Hyd	Anywhere	O1	Amino acid side chain
4-hydroxyglutamate	Glutamate	hydroxy	Hyd	Anywhere	O1	Amino acid side chain
(3S)-3-hydroxyhistidine	Histidine	hydroxy	Hyd	Anywhere	O1	Amino acid side chain
(3R,4S)-4-hydroxyisoleucine	Isoleucine	hydroxy	Hyd	Anywhere	O1	Amino acid side chain
5-hydroxylysine	Lysine	hydroxy	Hyd	Anywhere	O1	Amino acid side chain
(5R)-5-hydroxylysine	Lysine	hydroxy	Hyd	Anywhere	O1	Amino acid side chain
(5S)-5-hydroxylysine	Lysine	hydroxy	Hyd	Anywhere	O1	Amino acid side chain

Methionine (R)-sulfoxide	Methionine	hydroxy	Hyd	Anywhere	O1	Amino acid side chain
Methionine (S)-sulfoxide	Methionine	hydroxy	Hyd	Anywhere	O1	Amino acid side chain
Methionine sulfoxide	Methionine	hydroxy	Hyd	Anywhere	O1	Amino acid side chain
3-hydroxyphenylalanine	Phenylalanine	hydroxy	Hyd	Anywhere	O1	Amino acid side chain
3-hydroxyproline	Proline	hydroxy	Hyd	Anywhere	O1	Amino acid side chain
4-hydroxyproline	Proline	hydroxy	Hyd	Anywhere	O1	Amino acid side chain
Hydroxyproline	Proline	Hydroxy	Hyd	Anywhere	O1	Amino acid side chain
3-hydroxytryptophan	Tryptophan	hydroxy	Hyd	Anywhere	O1	Amino acid side chain
7'-hydroxytryptophan	Tryptophan	hydroxy	Hyd	Anywhere	O1	Amino acid side chain
3',4'-dihydroxyphenylalanine	Tyrosine	hydroxy	Hyd	Anywhere	O1	Amino acid side chain
3-hydroxyvaline	Valine	hydroxy	Hyd	Anywhere	O1	Amino acid side chain
D-4-hydroxyvaline	Valine	hydroxy	Hyd	Protein core	O1	Amino acid backbone
N6-lipoyllysine	Lysine	lipoyl	li	Anywhere	C14 H24 N2 O2 S2	Amino acid side chain
N-methylalanine	Alanine	methyl	Me	N-terminal	C1 H2	Amino acid backbone
5-methylarginine	Arginine	methyl	Me	Anywhere	C1 H2	Amino acid side chain
N5-methylarginine	Arginine	methyl	Me	Anywhere	C1 H2	Amino acid side chain
Omega-N-methylarginine	Arginine	methyl	Me	Anywhere	C1 H2	Amino acid side chain
N4-methylasparagine	Asparagine	methyl	Me	Anywhere	C1 H2	Amino acid side chain
S-methylcysteine	Cysteine	methyl	Me	Anywhere	C1 H2	Amino acid side chain
Cysteine methyl ester	Cysteine	methyl	Me	C-terminal	C1 H2	Amino acid backbone
Glutamate methyl ester (Glu)	Glutamate	methyl	Me	Anywhere	C1 H2	Amino acid side chain
N5-methylglutamine	Glutamine	methyl	Me	Anywhere	C1 H2	Amino acid side chain
2-methylglutamine	Glutamine	methyl	Me	Protein core	C1 H2	Amino acid backbone
N-methylglycine	Glycine	methyl	Me	Anywhere	C1 H2	Amino acid backbone
Methylhistidine	Histidine	Methyl	Me	Anywhere	C1 H2	Amino acid side chain
Pros-methylhistidine	Histidine	methyl	Me	Anywhere	C1 H2	Amino acid side chain

Tele-methylhistidine	Histidine	methyl	Me	Anywhere	C1 H2	Amino acid side chain
N-methylisoleucine	Isoleucine	methyl	Me	N-terminal	C1 H2	Amino acid backbone
Leucine methyl ester	Leucine	methyl	Me	C-terminal	C1 H2	Amino acid backbone
N-methylleucine	Leucine	methyl	Me	N-terminal	C1 H2	Amino acid backbone
N6-methyllysine	Lysine	methyl	Me	Anywhere	C1 H2	Amino acid side chain
Lysine methyl ester	Lysine	methyl	Me	C-terminal	C1 H2	Amino acid backbone
N-methylmethionine	Methionine	methyl	Me	N-terminal	C1 H2	Amino acid backbone
N-methylphenylalanine	Phenylalanine	methyl	Me	N-terminal	C1 H2	Amino acid backbone
N-methylproline	Proline	methyl	Me	N-terminal	C1 H2	Amino acid backbone
N-methylserine	Serine	methyl	Me	N-terminal	C1 H2	Amino acid backbone
O-methylthreonine	Threonine	methyl	Me	Anywhere	C1 H2	Amino acid side chain
Threonine methyl ester	Threonine	methyl	Me	C-terminal	C1 H2	Amino acid backbone
N-methyltyrosine	Tyrosine	methyl	Me	N-terminal	C1 H2	Amino acid backbone
3-methylthioaspartic acid	Aspartate	methylthio	MT	Anywhere	C1 H2 S1	Amino acid side chain
Pyrrolidone carboxylic acid	Glutamine	NH3loss	LoA	N-terminal	H-3 N-1	Amino acid backbone
Pyruvic acid (Ser)	Serine	NH3loss	LoA	N-terminal	H-3 N-1	Amino acid backbone
2-oxobutanoic acid	Threonine	NH3loss	LoA	N-terminal	H-3 N-1	Amino acid backbone
O-(pantetheine 4'-phosphoryl)serine	Serine	pantetheinephosphoryl	PaP	Anywhere	C11 H21 N2 O6 P1 S1	Amino acid side chain
Phosphoarginine	Arginine	Phospho	P	Anywhere	H1 O3 P1	Amino acid side chain
4-aspartylphosphate	Aspartate	Phospho	P	Anywhere	H1 O3 P1	Amino acid side chain
Phosphocysteine	Cysteine	Phospho	P	Anywhere	H1 O3 P1	Amino acid side chain
Phosphohistidine	Histidine	Phospho	P	Anywhere	H1 O3 P1	Amino acid side chain
Pros-phosphohistidine	Histidine	phospho	P	Anywhere	H1 O3 P1	Amino acid side chain
Tele-phosphohistidine	Histidine	phospho	P	Anywhere	H1 O3 P1	Amino acid side chain
Phosphoserine	Serine	Phospho	P	Anywhere	H1 O3 P1	Amino acid side chain
Phosphothreonine	Threonine	Phospho	P	Anywhere	H1 O3 P1	Amino acid side chain

Phosphotyrosine	Tyrosine	Phospho	P	Anywhere	H1 O3 P1	Amino acid side chain
O-(phosphoribosyl dephospho-coenzyme A)serine	Serine	phosphoribosyl	PR	Anywhere	C26 H42 N7 O19 P3 S1	Amino acid side chain
N6-(pyridoxal phosphate)lysine	Lysine	pyridoxalphospho	PyP	Anywhere	C8 H8 N1 O5 P1	Amino acid side chain
S-selanylcysteine	Cysteine	selanyl	Se	Anywhere	Se1	Amino acid side chain
N6-succinyllysine	Lysine	succinyl	succinyl	Anywhere	C4 H4 O3	Amino acid side chain
N2-succinyltryptophan	Tryptophan	succinyl	succinyl	N-terminal	C4 H4 O3	Amino acid backbone
1-thioglycine	Glycine	thio	thio	Protein core	O-1 S1	Amino acid backbone
N,N,N-trimethylalanine	Alanine	trimethyl	tMe	N-terminal	C3 H7	Amino acid backbone
N,N,N-trimethylglycine	Glycine	trimethyl	tMe	N-terminal	C3 H7	Amino acid backbone
N6,N6,N6-trimethyllysine	Lysine	trimethyl	tMe	Anywhere	C3 H7	Amino acid side chain
N,N,N-trimethylserine	Serine	trimethyl	tMe	N-terminal	C3 H7	Amino acid backbone
O-UMP-tyrosine	Tyrosine	UMP	UMP	Anywhere	C9 H11 N2 O8 P1	Amino acid side chain
N6-(3,6-diaminohexanoyl)-5-hydroxylysine	Lysine	diaminohexanoylhydroxy	DAHH	N-terminal	C6H12N2O2	Amino acid side chain

Scheme S1. OIE_CARE source code as implemented in ProteinGoggle

```
using System;
using SystemCollectionsGeneric;
using SystemIO;
using SystemRuntimeSerializationFormattersBinary;
using SystemLinq;
using SystemText;
using SystemData;
using MySqlDataMySqlClient;
using ProteinGoggleDAL;
using ProteinGoggleModel;
using ProteinGoggleBLLBase64Csharp;
using SystemReflection;
namespace ProteinGoggleBLL
{
	public class PeptideModResBLL
	{
		/// <summary>
		///
		/// </summary>
		public bool getComparePercentage(List<MassPoint> msReturn, int pmIndex, List<MassPoint> msFromDB, double fat)
		{
			for (int i = 0; i < msReturnCount; i++)
			{
				if (msReturn[i]Intensity < 005)
					msReturn[i]Intensity = 00;
			}
			double maxInten = msReturn[pmIndex]Intensity;
```

```
double nowInten = 0;
int IPADOCount = 0;
int IPMDOCount = 0;
int IPMDOMCount = 0;
int IPADOMCount = 0;
int k = 0;
for (int i = 0; i < msReturnCount; i++)
{
    if (msFromDB[i]Intensity * 100 > fat)
    {
        k++;
        nowInten = msReturn[i]Intensity / maxInten;
        if (doubleIsNaN(msReturn[i]Mass) || msReturn[i]Mass < 0000001)
        {
            return false;
        }
        if (doubleIsNaN(nowInten))
        {
            nowInten = 00;
        }
        double deviationInten = (nowInten - msFromDB[i]Intensity) * 100;
        double deviationMZ = (msReturn[i]Mass - msFromDB[i]Mass) / msFromDB[i]Mass * 10000000;
        if (deviationMZ > 0)
            msReturn[i]IPMD_R = (int)(deviationMZ + 05);
        else
            msReturn[i]IPMD_R = (int)(deviationMZ - 05);
        msReturn[i]IPMD_R = deviationMZ;
        msReturn[i]IPAD_R = deviationInten;
```

```
        msReturn[i]IntensityPercentage = nowInten;
        if (SystemMathAbs(deviationInten) == 10000000)
        {
            continue;
        }
        if (SystemMathAbs(deviationMZ) == 10000000)
        {
            IPMDOCount = IPMDOCount + 1;
            continue;
        }
        if (SystemMathAbs(deviationMZ) > testParameterIPMDOM)
        {

            IPMDOCount = IPMDOCount + 1;
        }
        if (SystemMathAbs(deviationMZ) > testParameterIPMD && SystemMathAbs(deviationMZ) < testParameterIPMDOM)
        {
            IPMDOCount = IPMDOCount + 1;
        }
        if (SystemMathAbs(deviationInten) > testParameterIPADOM)
        {
            IPADOMCount = IPADOMCount + 1;
        }
        if (SystemMathAbs(deviationInten) > testParameterIPAD)
        {
            IPADOCount = IPADOCount + 1;
        }
    }
```

```
        else
        {
            nowInten = msReturn[i]Intensity / maxInten;
            if (doubleIsNaN(msReturn[i]Mass) || msReturn[i]Mass < 0000001)
            {
                continue;
            }
            if (doubleIsNaN(nowInten))
            {
                nowInten = 00;
            }
            double deviationInten = (nowInten - msFromDB[i]Intensity) * 100;
           double deviationMZ = (msReturn[i]Mass - msFromDB[i]Mass) / msFromDB[i]Mass * 10000000;
            if (deviationMZ > 0)
                msReturn[i]IPMD_R = (int)(deviationMZ + 05);
            else
                msReturn[i]IPMD_R = (int)(deviationMZ - 05);
            msReturn[i]IPMD_R = deviationMZ;
            msReturn[i]IPAD_R = deviationInten;
            msReturn[i]IntensityPercentage = nowInten;
        }
    }
    if (IPMDOCount == 0)
    {
        IPMDOCount = 0;
    }
    if (IPMDOCount / ConvertToDouble(k) * 100 > testParameterIPMDO)
    {
```

```
        return false;
}
int pmIndexLeft = pmIndex;
int pmIndexRight = pmIndex;
int tempPmIndex = pmIndex;
bool needNext = true; ;
for (int lr = 0; lr < 2; lr++)
{
        do
        {
                if ((IPADOCount / ConvertToDouble(k) * 100 > testParameterIPADO) || IPADOMCount > 0)
                {
                        if (lr == 0)
                        {
                                pmIndexLeft--;
                                tempPmIndex = pmIndexLeft;
                        }
                        else
                        {
                                pmIndexRight++;
                                tempPmIndex = pmIndexRight;
                        }
                        if (msReturnCount == 1)
                                return false;
                        if (tempPmIndex < 0 || tempPmIndex >= msFromDBCount())
                                break;
                        if (msReturn[tempPmIndex]Intensity == 00)
                                continue;
```

```
        if (msFromDB[tempPmIndex]Intensity * 100 < testParameterIPACO)
            break;
        maxInten = msReturn[tempPmIndex]Intensity / msFromDB[tempPmIndex]Intensity;
        IPADOCount = 0;
        for (int i = 0; i < msReturnCount; i++)
        {
            nowInten = msReturn[i]Intensity / maxInten;
            if (doubleIsNaN(nowInten))
            {
                nowInten = 00;
            }
            msReturn[i]IntensityPercentage = nowInten;
            double deviationInten = (nowInten - msFromDB[i]Intensity) * 100;
            msReturn[i]IPAD_R = deviationInten;
            if (SystemMathAbs(deviationInten) > testParameterIPAD && (msFromDB[i]Intensity * 100 > fat))
            {
                IPADOCount = IPADOCount + 1;
            }
        }
        if (IPADOCount / ConvertToDouble(k) * 100 > testParameterIPADO)
        {
            needNext = true;
        }
        else
        {
            needNext = false;
        }
    }
```

```
                else
                    return true;
            }
            while (needNext);
            if (!needNext)
                return true;
        }
        return false;
    }
    public ModResModel maxIntenIonSelect(List<ModResModel> lsMaxOver)
    {
        ModResModel mr = new ModResModel();
        List<ModResModel> ls_MH = new List<ModResModel>();
        List<ModResModel> ls_y = new List<ModResModel>();
        List<ModResModel> ls_yNL = new List<ModResModel>();
        List<ModResModel> ls_b = new List<ModResModel>();
        List<ModResModel> ls_bNL = new List<ModResModel>();
        List<ModResModel> ls_a = new List<ModResModel>();
        List<ModResModel> ls_aNL = new List<ModResModel>();

        List<ModResModel> ls_x = new List<ModResModel>();
        List<ModResModel> ls_xNL = new List<ModResModel>();
        List<ModResModel> ls_c = new List<ModResModel>();
        List<ModResModel> ls_cNL = new List<ModResModel>();
        List<ModResModel> ls_z = new List<ModResModel>();
        List<ModResModel> ls_zNL = new List<ModResModel>();

        List<ModResModel> ls_xp1 = new List<ModResModel>();
```

```
List<ModResModel> ls_ap1 = new List<ModResModel>();
List<ModResModel> ls_ys1 = new List<ModResModel>();

List<ModResModel> ls_i = new List<ModResModel>();
List<ModResModel> ls_iNL = new List<ModResModel>();
foreach (ModResModel mrm in lsMaxOver)
{

    if (mrmIDFirst() == 'y' && (mrmIDContains("a") || mrmIDContains("b")) && (mrmIDContains("H2O") || mrmIDContains("NH3")))
        ls_iNLAdd(mrm);
    if (mrmIDFirst() == 'y' && !mrmIDContains("H2O") && !mrmIDContains("NH3") && (mrmIDContains("a") || mrmIDContains("b")))
        ls_iAdd(mrm);
    if (mrmIDFirst() == 'a' && (mrmIDContains("H2O") || mrmIDContains("NH3")))
        ls_aNLAdd(mrm);
    if (mrmIDFirst() == 'b' && (mrmIDContains("H2O") || mrmIDContains("NH3")))
        ls_bNLAdd(mrm);
    if (mrmIDFirst() == 'y' && !mrmIDContains("a") && !mrmIDContains("b") && (mrmIDContains("H2O") || mrmIDContains("NH3")))
        ls_yNLAdd(mrm);
    if (mrmIDFirst() == 'a' && !mrmIDContains("H2O") && !mrmIDContains("NH3") && !mrmIDContains("+H-"))
        ls_aAdd(mrm);
    if (mrmIDFirst() == 'b' && !mrmIDContains("H2O") && !mrmIDContains("NH3"))
        ls_bAdd(mrm);
    if (mrmIDFirst() == 'y' && !mrmIDContains("H2O") && !mrmIDContains("NH3") && !mrmIDContains("a") && !mrmIDContains("-H-")
&& !mrmIDContains("b"))
        ls_yAdd(mrm);

    if (mrmIDFirst() == 'c' && (mrmIDContains("H2O") || mrmIDContains("NH3")))
        ls_cNLAdd(mrm);
```

```
        if (mrmIDFirst() == 'z' && (mrmIDContains("H2O") || mrmIDContains("NH3")))
            ls_zNLAdd(mrm);
        if (mrmIDFirst() == 'x' && !mrmIDContains("a") && !mrmIDContains("b") && (mrmIDContains("H2O") || mrmIDContains("NH3")))
            ls_xNLAdd(mrm);
        if (mrmIDFirst() == 'c' && !mrmIDContains("H2O") && !mrmIDContains("NH3"))
            ls_cAdd(mrm);
        if (mrmIDFirst() == 'z' && !mrmIDContains("H2O") && !mrmIDContains("NH3"))
            ls_zAdd(mrm);
        if (mrmIDFirst() == 'x' && !mrmIDContains("H2O") && !mrmIDContains("NH3") && !mrmIDContains("+H-"))
            ls_xAdd(mrm);

        if (mrmIDFirst() == 'x' && mrmIDContains("+H-"))
            ls_xp1Add(mrm);
        if (mrmIDFirst() == 'M')
            ls_MHAdd(mrm);
        if (mrmIDFirst() == 'a' && mrmIDContains("+H-"))
            ls_ap1Add(mrm);
        if (mrmIDFirst() == 'y' && mrmIDContains("-H-"))
            ls_ys1Add(mrm);

    };
    mr = lsIonSelect(ls_y);
    if (mr != null)
    {
        return mr;
    }
    mr = lsIonSelect(ls_b);
    if (mr != null)
```

```
{
        return mr;
}
mr = lsIonSelect(ls_c);
if (mr != null)
{
        return mr;
}
mr = lsIonSelect(ls_z);
if (mr != null)
{
        return mr;
}
mr = lsIonSelect(ls_x);
if (mr != null)
{
        return mr;
}
mr = lsIonSelect(ls_a);
if (mr != null)
{
        return mr;
}
mr = lsIonSelect(ls_yNL);
if (mr != null)
{
        return mr;
}
```

```
mr = lsIonSelect(ls_bNL);
if (mr != null)
{
        return mr;
}
mr = lsIonSelect(ls_cNL);
if (mr != null)
{
        return mr;
}
mr = lsIonSelect(ls_zNL);
if (mr != null)
{
        return mr;
}
mr = lsIonSelect(ls_xNL);
if (mr != null)
{
        return mr;
}

mr = lsIonSelect(ls_aNL);
if (mr != null)
{
        return mr;
}
mr = lsIonSelect(ls_ys1);
if (mr != null)
```

```
{
    return mr;
}
mr = lsIonSelect(ls_ap1);
if (mr != null)
{
    return mr;
}
mr = lsIonSelect(ls_xp1);
if (mr != null)
{
    return mr;
}
mr = lsIonSelect(ls_MH);
if (mr != null)
{
    return mr;
}



mr = lsIonSelect(ls_i);
if (mr != null)
{
    return mr;
}
mr = lsIonSelect(ls_iNL);
if (mr != null)
```

```
        {
                return mr;
        }
        return lsMaxOver[0];


}
public ModResModel lsIonSelect(List<ModResModel> ls_ionseries)
{
        ModResModel mr = new ModResModel();
        if (ls_ionseriesCount == 1)
                mr = ls_ionseries[0];
        else if (ls_ionseriesCount > 1)
        {
                int maxN = 0;
                int tempN, tempNAboveIPACO, tempN_TheoAboveIPACO;
                int nomissing = 0;
                double MaxR = 00;
                int indexN = 0;
                for (int i = 0; i < ls_ionseriesCount; i++)
                {
                        tempN = (from p in ls_ionseries[i]Mass_Point_FromFile where pMass != 00 select i)Count();
                        if (tempN > maxN)
                        {
                                maxN = tempN;
                                indexN = i;
                        }
                }
```

```
                mr = ls_ionseries[indexN];
            }
            else
                mr = null;
            return mr;
        }
        public void lsIonDivide(List<ModResModel> lst, List<ModResModel> nomissing, List<ModResModel> missing)
        {
            if (lstCount > 0)
            {
                for (int i = 0; i < lstCount; i++)
                {
                    if (lst[i]NoMissing)
                        nomissingAdd(lst[i]);
                    else
                        missingAdd(lst[i]);
                }
            }
        }

        public List<ModResModel> calcu_overlap(List<ModResModel> MatchedMS2, List<List<string>> overlapProducts, List<ModResModel> lsdele, Dictionary<double, List<string>> overlapIE, Dictionary<double, double> InterpretedIPs, List<string> id_M, List<string> id_S, string scanType)
        {
            #region// IonSeries
            int[] id_S_index = new int[id_SCount];
            int[] id_M_index = new int[id_MCount];
            List<ModResModel>[] matchedMS2_IonSeries = new List<ModResModel>[8];
```

```
for (int i = 0; i < 8; i++)
{
        matchedMS2_IonSeries[i] = new List<ModResModel>();
}
if (scanType == "CID" || scanType == "HCD")
{
        #region//CID
        foreach (ModResModel mrm in MatchedMS2)
        {

                string id = mrmID + "-" + mrmZ + "+";
                if (idContains("("))
                        id = idSplit(new char[] { '(', ')' })[0] + idSplit(new char[] { '(', ')' })[2];
                if (id_SContains(id))
                        id_S_index[id_SIndexOf(id)] = MatchedMS2IndexOf(mrm);
                if (id_MContains(id))
                {
                        mrmIonStyle = 'M';
                        id_M_index[id_MIndexOf(id)] = MatchedMS2IndexOf(mrm);
                        for (int imrm = 0; imrm < mrmMass_Point_FromFileCount; imrm++)
                        {
                                if (mrmMass_Point_FromFile[imrm]Mass != 00)
                                        overlapIE[mrmMass_Point_FromFile[imrm]Mass]Remove(id);
                        }

                }
                else
                {
```

```
if ((mrmIDFirst() == 'y' || mrmIDFirst() == 'b'))
{
        if (mrmIDContains("-a") || mrmIDContains("-b"))
        {
                if (mrmIDContains("-2H2O") || mrmIDContains("-2NH3") || mrmIDContains("-H2O-NH3"))
                {//i-2NL--7
                        matchedMS2_IonSeries[7]Add(mrm);
                }
                else if (mrmIDContains("-H2O") || mrmIDContains("-NH3"))
                {//i-NL--6
                        matchedMS2_IonSeries[6]Add(mrm);
                }
                else
                {//i--5
                        matchedMS2_IonSeries[5]Add(mrm);
                }

        }
        else
        {
                if (mrmIDContains("-2H2O") || mrmIDContains("-2NH3") || mrmIDContains("-H2O-NH3"))
                {//y,b-2NL--3
                        matchedMS2_IonSeries[3]Add(mrm);
                }
                else if (mrmIDContains("-H2O") || mrmIDContains("-NH3"))
                {//y,b-NL--2
                        matchedMS2_IonSeries[2]Add(mrm);
```

```
                }
                else
                {//y,b nomissing--0
                    if (mrmNoMissing)
                        matchedMS2_IonSeries[0]Add(mrm);
                    else//y,b missing--1
                        matchedMS2_IonSeries[1]Add(mrm);
                }

            }
        }
        else if (mrmIDFirst() == 'a')
        {

            if (mrmIDContains("H2O") || mrmIDContains("NH3"))
            {//a-NL--3
                matchedMS2_IonSeries[3]Add(mrm);
            }
            else
            {//a--2
                matchedMS2_IonSeries[2]Add(mrm);
            }
        }
        else
        {//MH--4
            matchedMS2_IonSeries[4]Add(mrm);
        }
    }
```

```
        }
        #endregion
}
else if (scanType == "ETD" || scanType == "ECD")
{
        #region//ETD
        foreach (ModResModel mrm in MatchedMS2)
        {
                string id = mrmID + "-" + mrmZ + "+";
                if (idContains("("))
                        id = idSplit(new char[] { '(', ')' })[0] + idSplit(new char[] { '(', ')' })[2];
                if (id_SContains(id))
                        id_S_index[id_SIndexOf(id)] = MatchedMS2IndexOf(mrm);
                if (id_MContains(id))
                {
                        mrmIonStyle = 'M';
                        id_M_index[id_MIndexOf(id)] = MatchedMS2IndexOf(mrm);
                        for (int imrm = 0; imrm < mrmMass_Point_FromFileCount; imrm++)
                        {
                                if (mrmMass_Point_FromFile[imrm]Mass != 00)
                                        overlapIE[mrmMass_Point_FromFile[imrm]Mass]Remove(id);
                        }

                }
                else
                {

                        if ((mrmIDFirst() == 'z' || mrmIDFirst() == 'c'))
```

```
                {

                    if (mrmIDContains("-2H2O") || mrmIDContains("-2NH3") || mrmIDContains("-H2O-NH3"))
                    {//c,z-2NL--2
                        matchedMS2_IonSeries[2]Add(mrm);
                    }
                    else if (mrmIDContains("-H2O") || mrmIDContains("-NH3"))
                    {//c,z-NL--1
                        matchedMS2_IonSeries[1]Add(mrm);
                    }
                    else
                    {//c,z--0
                        matchedMS2_IonSeries[0]Add(mrm);
                    }

                }
                else
                {//MH--3
                    matchedMS2_IonSeries[3]Add(mrm);
                }
            }
        }
        #endregion
    }
    else
    {
```

```
#region//UVPD
foreach (ModResModel mrm in MatchedMS2)
{
    string id = mrmID + "-" + mrmZ + "+";
    if (idContains("("))
        id = idSplit(new char[] { '(', ')' })[0] + idSplit(new char[] { '(', ')' })[2];
    if (id_SContains(id))
        id_S_index[id_SIndexOf(id)] = MatchedMS2IndexOf(mrm);
    if (id_MContains(id))
    {
        mrmIonStyle = 'M';
        id_M_index[id_MIndexOf(id)] = MatchedMS2IndexOf(mrm);
        for (int imrm = 0; imrm < mrmMass_Point_FromFileCount; imrm++)
        {
            if (mrmMass_Point_FromFile[imrm]Mass != 00)
                overlapIE[mrmMass_Point_FromFile[imrm]Mass]Remove(id);
        }

    }
    else
    {

        if ((mrmIDFirst() == 'y' || mrmIDFirst() == 'b' || mrmIDFirst() == 'c' || mrmIDFirst() == 'z' || mrmIDFirst() == 'x'))
        {
            if (mrmIDContains("-H-") || mrmIDContains("+H-"))
            {
                //a+1,x+1,y-1     --3
                matchedMS2_IonSeries[3]Add(mrm);
```

```
        }
        else
        {
            if (mrmIDContains("-2H2O") || mrmIDContains("-2NH3") || mrmIDContains("-H2O-NH3"))
            {//y,b-2NL--2
                matchedMS2_IonSeries[2]Add(mrm);
            }
            else if (mrmIDContains("-H2O") || mrmIDContains("-NH3"))
            {//y,b-NL--1
                matchedMS2_IonSeries[1]Add(mrm);
            }
            else
            {//y,b--0
                matchedMS2_IonSeries[0]Add(mrm);
            }

        }
    }
    else if (mrmIDFirst() == 'a')
    {

        if (mrmIDContains("H2O") || mrmIDContains("NH3"))
        {//a-NL--2
            matchedMS2_IonSeries[2]Add(mrm);
        }
        else
        {//a--1
            matchedMS2_IonSeries[1]Add(mrm);
```

```
                }
            }
            else
            {//MH--4
                matchedMS2_IonSeries[4]Add(mrm);
            }
        }
    }
    #endregion
}
for (int iid_m = id_M_indexLength - 1; iid_m >= 0; iid_m--)
{
    bool isNotMulti = false;
    {

        for (int mpf = 0; mpf < MatchedMS2[id_M_index[iid_m]]Mass_Point_FromFileCount && mpf < MatchedMS2[id_S_index[iid_m]]Mass_Point_FromFileCount; mpf++)
        {
            if (MatchedMS2[id_M_index[iid_m]]Mass_Point_FromFile[mpf]Mass != MatchedMS2[id_M_index[iid_m]]Mass_Point_FromFile[mpf]Mass)
            {
                isNotMulti = true;
                break;
            }
        }
    }
    if (isNotMulti)
    {
```

```
ModResModel mrm = MatchedMS2[id_M_index[iid_m]];
id_M_index[iid_m] = -1;
id_S_index[iid_m] = -1;
mrmIonStyle = '\0';
if (scanType == "CID" || scanType == "HCD")
{
        #region//CID


        if ((mrmIDFirst() == 'y' || mrmIDFirst() == 'b'))
        {
                if (mrmIDContains("-a") || mrmIDContains("-b"))
                {
                        if (mrmIDContains("-2H2O") || mrmIDContains("-2NH3") || mrmIDContains("-H2O-NH3"))
                        {//i-2NL--7
                                matchedMS2_IonSeries[7]Add(mrm);
                        }
                        else if (mrmIDContains("-H2O") || mrmIDContains("-NH3"))
                        {//i-NL--6
                                matchedMS2_IonSeries[6]Add(mrm);
                        }
                        else
                        {//i--5
                                matchedMS2_IonSeries[5]Add(mrm);
                        }

                }
                else
```

```
            {
                if (mrmIDContains("-2H2O") || mrmIDContains("-2NH3") || mrmIDContains("-H2O-NH3"))
                {//y,b-2NL--3
                    matchedMS2_IonSeries[3]Add(mrm);
                }
                else if (mrmIDContains("-H2O") || mrmIDContains("-NH3"))
                {//y,b-NL--2
                    matchedMS2_IonSeries[2]Add(mrm);
                }
                else
                {//y,b nomissing--0
                    if (mrmNoMissing)
                        matchedMS2_IonSeries[0]Add(mrm);
                    else//y,b missing--1
                        matchedMS2_IonSeries[1]Add(mrm);
                }

            }
        }
        else if (mrmIDFirst() == 'a')
        {

            if (mrmIDContains("H2O") || mrmIDContains("NH3"))
            {//a-NL--3
                matchedMS2_IonSeries[3]Add(mrm);
            }
            else
            {//a--2
```

```
                matchedMS2_IonSeries[2]Add(mrm);
            }
        }
        else
        {//MH--4
            matchedMS2_IonSeries[4]Add(mrm);
        }

        #endregion
    }
    else if (scanType == "ETD" || scanType == "ECD")
    {
        #region//ETD
        if ((mrmIDFirst() == 'z' || mrmIDFirst() == 'c'))
        {


            if (mrmIDContains("-2H2O") || mrmIDContains("-2NH3") || mrmIDContains("-H2O-NH3"))
            {//c,z-2NL--2
                matchedMS2_IonSeries[2]Add(mrm);
            }
            else if (mrmIDContains("-H2O") || mrmIDContains("-NH3"))
            {//c,z-NL--1
                matchedMS2_IonSeries[1]Add(mrm);
            }
            else
            {//c,z--0
                matchedMS2_IonSeries[0]Add(mrm);
```

```
                }


        }
        else
        {//MH--3
                matchedMS2_IonSeries[3]Add(mrm);
        }

        #endregion
}
else
{
        #region//UVPD

        if ((mrmIDFirst() == 'y' || mrmIDFirst() == 'b' || mrmIDFirst() == 'c' || mrmIDFirst() == 'z' || mrmIDFirst() == 'x'))
        {
                if (mrmIDContains("-H-") || mrmIDContains("+H-"))
                {
                        //a+1,x+1,y-1      --3
                        matchedMS2_IonSeries[3]Add(mrm);
                }
                else
                {
                        if (mrmIDContains("-2H2O") || mrmIDContains("-2NH3") || mrmIDContains("-H2O-NH3"))
                        {//y,b-2NL--2
                                matchedMS2_IonSeries[2]Add(mrm);
                        }
```

```
            else if (mrmIDContains("-H2O") || mrmIDContains("-NH3"))
            {//y,b-NL--1
                matchedMS2_IonSeries[1]Add(mrm);
            }
            else
            {//y,b--0
                matchedMS2_IonSeries[0]Add(mrm);
            }

        }
    }
    else if (mrmIDFirst() == 'a')
    {

        if (mrmIDContains("H2O") || mrmIDContains("NH3"))
        {//a-NL--2
            matchedMS2_IonSeries[2]Add(mrm);
        }
        else
        {//a--1
            matchedMS2_IonSeries[1]Add(mrm);
        }
    }
    else
    {//MH--4
        matchedMS2_IonSeries[4]Add(mrm);
    }
    #endregion
```

```
            }
        }

    }


    #endregion
    for (int iIs = 0; iIs < matchedMS2_IonSeriesLength; iIs++)
    {
        int nextcount = 0;
        for (int k = iIs + 1; k < matchedMS2_IonSeriesLength; k++)
        {
            nextcount += matchedMS2_IonSeries[k]Count();
        }
        List<ModResModel> nextlevel;
        if (nextcount > 0)
            nextlevel = new List<ModResModel>();
        else
            nextlevel = null;
        if (matchedMS2_IonSeries[iIs]Count != 0)
        {
            matchedMS2_IonSeries[iIs] = matchedMS2_IonSeries[iIs]OrderBy(ms => msID)ToList();
            matchedMS2_IonSeries[iIs] = matchedMS2_IonSeries[iIs]OrderBy(ms => msMass_Point[0]Mass)ToList();
            List<ModResModel> cluster = new List<ModResModel>();
            List<ModResModel> sameincluster = new List<ModResModel>();
            List<int> same = new List<int>();
            List<int> ionNumber = new List<int>();
            List<int> clusterIonNumber = new List<int>();
```

```
List<int> clusterAllOverlapIon = new List<int>();
List<int> overlapNumber = new List<int>();
List<int> countOfOverlap = new List<int>();
List<ModResModel> matchedSame = new List<ModResModel>();
List<int> indexOfMathcedSame = new List<int>();
for (int m = 0; m < matchedMS2_IonSeries[iIs]Count; m++)
{
    if (ionNumberContains(m))
        continue;
    cluster = new List<ModResModel>();
    clusterIonNumber = new List<int>();
    clusterAllOverlapIon = new List<int>();
    clusterAdd(matchedMS2_IonSeries[iIs][m]);
    matchedMS2_IonSeries[iIs][m]IonStyle = 'O';
    cluster[clusterCount - 1]M = m;
    clusterIonNumberAdd(m);
    int k = 0;
    while (k < clusterCount)
    {
        for (int p = 0; p < cluster[k]Mass_PointCount; p++)
        {
            if (cluster[k]Mass_Point_FromFile[p]Mass == 00 || cluster[k]Mass_Point_FromFile[p]IPMDOM_R == -1)
                continue;
            if (cluster[k]Mass_Point_FromFile[p]Intensity == 00)
            {
                continue;
            }
            for (int n = clusterIonNumber[k] - 10 < 0 ? 0 : clusterIonNumber[k] - 10; n < matchedMS2_IonSeries[iIs]Count; n++)
```

```
{
    if (matchedMS2_IonSeries[iIs][n] != cluster[k])
    {
        if (cluster[k]Mass_Point[p]Mass < matchedMS2_IonSeries[iIs][n]Mass_Point[0]Mass - 10)
            break;
        for (int q = 0; q < matchedMS2_IonSeries[iIs][n]Mass_PointCount && q < matchedMS2_IonSeries[iIs][n]Mass_Point_FromFileCount; q++)
        {
            MassPoint loopUsePoint = matchedMS2_IonSeries[iIs][n]Mass_Point_FromFile[q];
            if (cluster[k]Mass_Point_FromFile[p]Mass == loopUsePointMass && loopUsePointMass != 00 && loopUsePointIPMDOM_R != -1)
            {
                if (InterpretedIPs[cluster[k]Mass_Point_FromFile[p]Mass] == 00)
                {
                    loopUsePointIntensity = 00;
                    continue;
                }
                cluster[k]Mass_Point_FromFile[p]IPMDOM_R = -1;
                matchedMS2_IonSeries[iIs][n]Mass_Point_FromFile[q]IPMDOM_R = -1;
                if (!clusterContains(matchedMS2_IonSeries[iIs][n]))
                {
                    clusterAdd(matchedMS2_IonSeries[iIs][n]);
                    matchedMS2_IonSeries[iIs][n]IonStyle = 'O';
                    cluster[clusterCount - 1]M = n;
                    clusterIonNumberAdd(n);
                }
            }
        }
```

```
                }
            }
            if (cluster[k]Mass_Point_FromFile[p]Mass != 00 && InterpretedIPs[cluster[k]Mass_Point_FromFile[p]Mass] == 00)
                cluster[k]Mass_Point_FromFile[p]Intensity = 00;
        }
        k++;
    }
    if (clusterCount == 1)
    {
        ModResModel mrm = cluster[0];
        mrmIonStyle = new char();
        for (int i = 0; i < mrmMass_Point_FromFileCount; i++)
        {
            if (mrmMass_Point_FromFile[i]Mass > lt && mrmMass_Point_FromFile[i]Mass < rt)
                i = i;
            if (mrmMass_Point_FromFile[i]Mass != 00)
            {
                if (mrmMass_Point_FromFile[i]Intensity > InterpretedIPs[mrmMass_Point_FromFile[i]Mass] && InterpretedIPs[mrmMass_Point_FromFile[i]Mass] != 00)
                    mrmIonStyle = 'O';
                mrmMass_Point_FromFile[i]Intensity = InterpretedIPs[mrmMass_Point_FromFile[i]Mass];
                mrmMass_Point_FromFile[i]IPMDOM_R = 00;
                InterpretedIPs[mrmMass_Point_FromFile[i]Mass] = 00;
            }
        }
        if (!getComparePercentage(mrmMass_Point_FromFile, mrmMS2MaxIntensityIndex, mrmMass_Point, testParameterIPACO) && mrmNoMissing)
        {
```

```
if (nextlevel != null)
{
    if (scanType == "HCD" || scanType == "CID")
    {
        if ((mrmIDFirst() == 'y' || mrmIDFirst() == 'b') && !mrmIDContains("H2O") && !mrmIDContains("NH3")
&& !mrmIDContains("-a") && !mrmIDContains("-b"))
        {
            for (int i = 0; i < mrmMass_Point_FromFileCount; i++)
            {
                if (mrmMass_Point_FromFile[i]Mass != 0)
                    InterpretedIPs[mrmMass_Point_FromFile[i]Mass] += mrmMass_Point_FromFile[i]Intensity;
            }
            nextlevelAdd(mrm);
        }
    }
    else if (scanType == "ETD" || scanType == "ECD")
    {
        if ((mrmIDFirst() == 'z' || mrmIDFirst() == 'c') && !mrmIDContains("H2O") && !mrmIDContains("NH3")
&& !mrmIDContains("-a") && !mrmIDContains("-b"))
        {
            for (int i = 0; i < mrmMass_Point_FromFileCount; i++)
            {
                if (mrmMass_Point_FromFile[i]Mass != 0)
                    InterpretedIPs[mrmMass_Point_FromFile[i]Mass] += mrmMass_Point_FromFile[i]Intensity;
            }
            nextlevelAdd(mrm);
        }
    }
```

```
				else
				{
					if ((mrmIDFirst() == 'z' || mrmIDFirst() == 'c') || (mrmIDFirst() == 'y' || mrmIDFirst() == 'b' || mrmIDFirst() == 'x')
						&& !mrmIDContains("H2O") && !mrmIDContains("NH3")
						&& !mrmIDContains("-a") && !mrmIDContains("-b")
						&& !mrmIDContains("-H-") && !mrmIDContains("+H-"))
					{
						for (int i = 0; i < mrmMass_Point_FromFileCount; i++)
						{
							if (mrmMass_Point_FromFile[i]Mass != 0)
								InterpretedIPs[mrmMass_Point_FromFile[i]Mass] += mrmMass_Point_FromFile[i]Intensity;
						}
						nextlevelAdd(mrm);
					}
				}
			}
		}
		if (mrmIonStyle == '\0')
			for (int i = 0; i < mrmMass_Point_FromFileCount; i++)
			{

				if (mrmMass_Point_FromFile[i]Mass != 00)
				{
					if (InterpretedIPs[mrmMass_Point_FromFile[i]Mass] > 0)
					{
						mrmIonStyle = 'O';
						break;
					}
```

```
                        }
                    }
                    continue;
                }
                else
                {
                    ionNumberAddRange(clusterIonNumber);
                    cluster = clusterOrderBy(ms => msMass_Point[0]Mass)ToList();
                    for (int i = 0; i < clusterCount; i++)
                    {
                        bool isallOverLap = true;
                        cluster[i]IonStyle = 'O';
                        ModResModel mr = cluster[i];
                        for (int j = 0; j < cluster[i]Mass_Point_FromFileCount; j++)
                        {
                            if (cluster[i]Mass_Point_FromFile[j]Intensity != 00 && cluster[i]Mass_Point_FromFile[j]Mass != 00 &&
cluster[i]Mass_Point_FromFile[j]IPMDOM_R != -1
                                && cluster[i]Mass_Point[j]Intensity * 100 > testParameterIPACO)
                            {
                                if (isallOverLap == true || cluster[i]Mass_Point[cluster[i]MS2BasePeakIndex]Intensity <
cluster[i]Mass_Point[j]Intensity)
                                    cluster[i]MS2BasePeakIndex = j;
                                isallOverLap = false;
                                if (cluster[i]Mass_Point[cluster[i]MS2BasePeakIndex]Intensity < cluster[i]Mass_Point[j]Intensity)
                                    break;
                            }
                        }
                        if (isallOverLap)
```

```
                {
                    for (int j = 0; j < i; j++)
                    {
                        if (cluster[i]Mass_Point_FromFile[cluster[i]MS2MaxIntensityIndex]Mass == cluster[j]Mass_Point_FromFile[cluster[j]MS2MaxIntensityIndex]Mass
                            && cluster[i]Z == cluster[j]Z
                            && i != j)
                        {
                            isallOverLap = false;
                            sameAdd((int)(cluster[j]M));
                            sameinclusterAdd(cluster[i]);
                            cluster[i]IonStyle = 'M';
                            break;
                        }
                    }
                    if (isallOverLap)
                    {
                        cluster[i]Alloverlapp = true;
                        cluster[i]IonStyle = 'E';
                        clusterAllOverlapIonAdd(i);
                    }
                }
            }
            if (clusterAllOverlapIonCount == 0)
            {
                cluster = calcu_clusteroverlap(cluster, null, lsdele, sameincluster, overlapIE, InterpretedIPs, nextlevel);
            }
            else
```

```
{
    cluster = calcu_clusteroverlap(cluster, clusterAllOverlapIon, lsdele, sameincluster, overlapIE, InterpretedIPs, nextlevel);
}
for (int i = 0; i < sameinclusterCount; i++)
{
    if (lsdeleContains(matchedMS2_IonSeries[iIs][same[i]]))
    {
        lsdeleAdd(sameincluster[i]);
    }
    else
    {
        for (int j = 0; j < sameincluster[i]Mass_Point_FromFileCount && j < 
matchedMS2_IonSeries[iIs][same[i]]Mass_Point_FromFileCount; j++)
        {
            if (sameincluster[i]Mass_Point_FromFile[j]Mass != 00)
            {
                if (sameincluster[i]Mass_Point_FromFile[j]Mass == 
matchedMS2_IonSeries[iIs][same[i]]Mass_Point_FromFile[j]Mass)
                    sameincluster[i]Mass_Point_FromFile[j]Intensity = 
matchedMS2_IonSeries[iIs][same[i]]Mass_Point_FromFile[j]Intensity;
                else
                {
                    if (overlapIE[sameincluster[i]Mass_Point_FromFile[j]Mass]Count == 0)
                        overlapIE[sameincluster[i]Mass_Point_FromFile[j]Mass]Remove(sameincluster[i]ID + "-" + 
sameincluster[i]Z + "+");
                    if (InterpretedIPsKeysContains(sameincluster[i]Mass_Point_FromFile[j]Mass) && 
overlapIE[sameincluster[i]Mass_Point_FromFile[j]Mass]Count == 0)
                        sameincluster[i]Mass_Point_FromFile[j]Intensity = 
```

```
InterpretedIPs[sameincluster[i]Mass_Point_FromFile[j]Mass];
                                                else
                                                    sameincluster[i]Mass_Point_FromFile[j]Intensity = 00;
                                            }
                                        }
                                    }
                                }
                            }
                            if (clusterCount > 1)
                            {
                                overlapProductsAdd((from p in cluster select pID + "-" + pZ + "+")ToList());
                            }
                        }
                    }
                }
                if (iIs + 1 < matchedMS2_IonSeriesCount())
                {
                    if (nextlevel != null)
                        matchedMS2_IonSeries[iIs + 1]AddRange(nextlevel);
                }
            }
            for (int ide = 0; ide < lsdeleCount; ide++)
            {
                foreach (MassPoint mp in lsdele[ide]Mass_Point_FromFile)
                {
                    if (mpMass != 00 && mpMass != null)
                        mpIntensity = InterpretedIPs[mpMass];
                }
```

```
            }
            for (int iid_m = 0; iid_m < id_M_indexLength; iid_m++)
            {
                if (id_S_index[iid_m] == -1)
                    continue;
                for (int mpf = 0; mpf < MatchedMS2[id_M_index[iid_m]]Mass_Point_FromFileCount; mpf++)
                {
                    if (mpf < MatchedMS2[id_S_index[iid_m]]Mass_Point_FromFileCount)
                    {
                        if (MatchedMS2[id_M_index[iid_m]]Mass_Point_FromFile[mpf]Mass == MatchedMS2[id_S_index[iid_m]]Mass_Point_FromFile[mpf]Mass)
                            MatchedMS2[id_M_index[iid_m]]Mass_Point_FromFile[mpf]Intensity = MatchedMS2[id_S_index[iid_m]]Mass_Point_FromFile[mpf]Intensity;
                        else if (MatchedMS2[id_M_index[iid_m]]Mass_Point_FromFile[mpf]Mass != 00)
                            MatchedMS2[id_M_index[iid_m]]Mass_Point_FromFile[mpf]Intensity = InterpretedIPs[MatchedMS2[id_M_index[iid_m]]Mass_Point_FromFile[mpf]Mass];
                    }
                    else if (MatchedMS2[id_M_index[iid_m]]Mass_Point_FromFile[mpf]Mass != 00)
                        MatchedMS2[id_M_index[iid_m]]Mass_Point_FromFile[mpf]Intensity = InterpretedIPs[MatchedMS2[id_M_index[iid_m]]Mass_Point_FromFile[mpf]Mass];
                }
                if (lsdeleContains(MatchedMS2[id_S_index[iid_m]]))
                    lsdeleAdd(MatchedMS2[id_M_index[iid_m]]);
            }
            return MatchedMS2;
        }
        public List<ModResModel> calcu_clusteroverlap(List<ModResModel> MatchedMS2, List<int> clusterAllOverlapIon, List<ModResModel> lsdeleAll, List<ModResModel> sameincluster, Dictionary<double, List<string>> overlapIEs, Dictionary<double, double> InterpretedIPs, List<ModResModel> nextlevel)
```

```
        {
            #region calcu_simple
            List<double> lsMass = new List<double>();
            List<ModResModel> lsAllOver = new List<ModResModel>();
            if (clusterAllOverlapIon != null)
            {
                List<ModResModel> lsDele = new List<ModResModel>();
                List<ModResModel> lsMaxOver = new List<ModResModel>();
                List<int> lsMaxOverInd = new List<int>();
                for (int iA = 0; iA < clusterAllOverlapIonCount; iA++)
                {
                    if (lsMaxOverIndContains(clusterAllOverlapIon[iA]))
                        continue;
                    ModResModel mA = MatchedMS2[clusterAllOverlapIon[iA]];
                    int MaxDBindex = mAMS2MaxIntensityIndex;
                    double MaxMass = mAMass_Point_FromFile[MaxDBindex]Mass;
                    lsMaxOver = (from p in clusterAllOverlapIon where MatchedMS2[p]Mass_Point_FromFile[MatchedMS2[p]MS2MaxIntensityIndex]Mass ==
MaxMass select MatchedMS2[p])ToList();
                    lsMaxOverIndAddRange((from p in clusterAllOverlapIon where
MatchedMS2[p]Mass_Point_FromFile[MatchedMS2[p]MS2MaxIntensityIndex]Mass == MaxMass select p)ToList());
                    if (lsMaxOverCount == 0)
                        continue;
                    ModResModel mr = new ModResModel();
                    List<ModResModel> lsMaxOverNOmissing = new List<ModResModel>();
                    List<ModResModel> lsMaxOverMissing = new List<ModResModel>();
                    lsIonDivide(lsMaxOver, lsMaxOverNOmissing, lsMaxOverMissing);
                    if (lsMaxOverNOmissingCount > 0)
                        mr = maxIntenIonSelect(lsMaxOverNOmissing);
```

```
else
        mr = maxIntenIonSelect(lsMaxOverMissing);
mA = mr;
lsDeleAddRange(lsMaxOver);
lsDeleRemove(mr);
lsAllOverAdd(mA);
lsMassAdd(MaxMass);
MaxDBindex = mAMS2MaxIntensityIndex;
double MaxInten = InterpretedIPs[mAMass_Point_FromFile[MaxDBindex]Mass];
List<ModResModel> lsM = new List<ModResModel>();
List<int> lsI = new List<int>();
for (int iMM = 0; iMM < MatchedMS2Count; iMM++)
{
        if (!clusterAllOverlapIonContains(iMM))
                for (int iMs = 0; iMs < MatchedMS2[iMM]Mass_Point_FromFileCount; iMs++)
                {
                        if (MatchedMS2[iMM]Mass_Point_FromFile[iMs]Mass == MaxMass)
                        {
                                if (MatchedMS2[iMM]Mass_Point[iMs]Intensity == 10 && MatchedMS2[iMM]Z == mAZ)
                                        continue;
                                else if (!sameinclusterContains(MatchedMS2[iMM]))
                                {
                                        lsMAdd(MatchedMS2[iMM]);
                                        lsIAdd(iMs);
                                }
                        }
                }
}
```

```
        double[] a = new double[lsICount];
        for (int ilsm = 0; ilsm < lsMCount; ilsm++)
        {
            a[ilsm] = lsM[ilsm]Mass_Point[lsI[ilsm]]Intensity *
                    InterpretedIPs[lsM[ilsm]Mass_Point_FromFile[lsM[ilsm]MS2BasePeakIndex]Mass]
                     / lsM[ilsm]Mass_Point[lsM[ilsm]MS2BasePeakIndex]Intensity;
        }
        if (aSum() < MaxInten || lsMCount() == 0 || lsM == null)
        {
            mAMass_Point_FromFile[MaxDBindex]IntensityPercentage = MaxInten - aSum();
        }
        else
        {
            lsDeleAdd(mA);
            lsAllOverRemove(mA);
            lsMassRemove(MaxMass);
        }
    }
    if (lsAllOverCount > 1)
    {
        List<ModResModel> cluster = new List<ModResModel>();
        List<int> ionNumber = new List<int>();
        List<int> clusterIonNumber = new List<int>();
        lsAllOver = lsAllOverOrderBy(m => mMass_Point[0]Mass)ToList();
        for (int m = 0; m < lsAllOverCount; m++)
        {
            if (ionNumberContains(m))
                continue;
```

```
cluster = new List<ModResModel>();
clusterIonNumber = new List<int>();
clusterAdd(lsAllOver[m]);
ionNumberAdd(m);
clusterIonNumberAdd(m);
int k = 0;
while (k < clusterCount)
{
    double tempmass = cluster[k]Mass_Point_FromFile[cluster[k]MS2MaxIntensityIndex]Mass;
    for (int n = 0; n < lsAllOverCount; n++)
    {
        if (ionNumberContains(n))
            continue;
        if (tempmass < lsAllOver[n]Mass_Point[0]Mass - 10)
            break;
        for (int q = 0; q < lsAllOver[n]Mass_PointCount && q < lsAllOver[n]Mass_Point_FromFileCount; q++)
        {
            MassPoint loopUsePoint = lsAllOver[n]Mass_Point_FromFile[q];
            if (tempmass == loopUsePointMass)
            {
                if (!clusterContains(lsAllOver[n]))
                {
                    clusterAdd(lsAllOver[n]);
                    ionNumberAdd(n);
                    clusterIonNumberAdd(n);
                }
            }
        }
```

```
        }
        for (int p = 0; p < cluster[k]Mass_PointCount && p < cluster[k]Mass_Point_FromFileCount; p++)
        {
            MassPoint loopUsePoint = cluster[k]Mass_Point_FromFile[p];
            for (int n = 0; n < lsAllOverCount; n++)
            {
                if (ionNumberContains(n))
                    continue;
                if (loopUsePointMass == lsAllOver[n]Mass_Point_FromFile[lsAllOver[n]MS2MaxIntensityIndex]Mass)
                {
                    if (!clusterContains(lsAllOver[n]))
                    {
                        clusterAdd(lsAllOver[n]);
                        ionNumberAdd(n);
                        clusterIonNumberAdd(n);
                    }
                }
            }
        }
        k++;
    }
    if (clusterCount > 1)
    {
        Matrix mt = new Matrix(clusterCount, clusterCount);
        Matrix mtv = new Matrix(clusterCount, clusterCount);
        double[] vectorFinalInten = new double[clusterCount];
        for (int iA = 0; iA < clusterCount; iA++)
        {
```

```
        ModResModel mA = cluster[iA];
        int MaxDBindex = mAMS2MaxIntensityIndex;
        double MaxMass = mAMass_Point_FromFile[MaxDBindex]Mass;
        double MaxInten = InterpretedIPs[MaxMass];
        mtvwrite(0, iA, MaxInten);
        List<int> lsI = new List<int>();
        for (int iMM = 0; iMM < clusterCount; iMM++)
        {
            double relInt = 0;
            for (int iMs = 0; iMs < cluster[iMM]Mass_Point_FromFileCount; iMs++)
            {
                if (cluster[iMM]Mass_Point_FromFile[iMs]Mass == MaxMass)
                {
                    relInt = cluster[iMM]Mass_Point[iMs]Intensity;
                    break;
                }
            }
            mtwrite(iMM, iA, relInt);
        }
    }
    Matrix mtf = new Matrix(clusterCount, clusterCount);
    Matrix mtr = new Matrix(clusterCount, clusterCount);
    int mtLengh = clusterCount;
    mtf = new Matrix(mtLengh, mtLengh);
    mtr = new Matrix(mtLengh, mtLengh);
    mtinverse(ref mt, ref mtr);
    mtmultiply(ref mtv, ref mtr, ref mtf);
    foreach (ModResModel mrm in cluster)
```

```
                {
                    if (mtfread(0, clusterIndexOf(mrm)) <= 00)
                    {
                        mrmMass_Point_FromFile[mrmMS2MaxIntensityIndex]IntensityPercentage = 1;
                    }
                    else
                    {
                        mrmMass_Point_FromFile[mrmMS2MaxIntensityIndex]IntensityPercentage = mtfread(0, clusterIndexOf(mrm));
                    }
                };

            }
        }
    }
    lsdeleAllAddRange(lsDele);
    foreach (ModResModel mrm in lsDele)
    {
        for (int i = 0; i < mrmMass_Point_FromFileCount; i++)
        {
            double MaxMass = mrmMass_Point_FromFile[i]Mass;
            if (MaxMass != 00)
            {
                List<string> lstemp = overlapIEs[MaxMass];
                if (mrmIDContains("("))
                {
                    string[] strgroup = mrmIDSplit(new char[] { '(', ')' })ToArray();
                    lstempRemove(strgroup[0] + "-" + mrmZ + "+");
                    string pre = strgroup[0]Split(new char[] { '-' })[0];
```

```
                        if (strgroupContains("H2O+NH3"))
                        {
                            lstempRemove(pre + "-H2O-NH3-" + mrmZ + "+");
                        }
                        if (strgroupContains("2NH3"))
                        {
                            lstempRemove(pre + "-2NH3-" + mrmZ + "+");
                        }
                        if (strgroupContains("NH3"))
                        {
                            lstempRemove(pre + "-NH3-" + mrmZ + "+");
                        }
                    }
                    else
                        lstempRemove(mrmID + "-" + mrmZ + "+");
                }
            }
            MatchedMS2Remove(mrm);
        };
    }
    List<int> templ = new List<int>();
    List<int> indexofMathcedsame = new List<int>();
    foreach (ModResModel mrm in sameincluster)
    {
        for (int i = 0; i < mrmMass_Point_FromFileCount; i++)
        {
            double MaxMass = mrmMass_Point_FromFile[i]Mass;
            if (MaxMass != 00)
```

```
                    {
                        List<string> lstemp = overlapIEs[MaxMass];
                        if (mrmIDContains("("))
                        {
                            string[] strgroup = mrmIDSplit(new char[] { '(', ')' })ToArray();
                            lstempRemove(strgroup[0] + "-" + mrmZ + "+");
                            string pre = strgroup[0]Split(new char[] { '-' })[0];
                            if (strgroupContains("H2O+NH3"))
                            {
                                lstempRemove(pre + "-H2O-NH3-" + mrmZ + "+");
                            }
                            if (strgroupContains("2NH3"))
                            {
                                lstempRemove(pre + "-2NH3-" + mrmZ + "+");
                            }
                            if (strgroupContains("NH3"))
                            {
                                lstempRemove(pre + "-NH3-" + mrmZ + "+");
                            }
                        }
                        else
                            lstempRemove(mrmID + "-" + mrmZ + "+");
                    }
                }
                MatchedMS2Remove(mrm);
            };
            if (MatchedMS2Count != 0)
            {
```

```
MatchedMS2 = MatchedMS2OrderBy(ms => msMass_Point[0]Mass)ToList();
List<ModResModel> matched_same = new List<ModResModel>();
List<double> remass = new List<double>();
for (int m = 0; m < MatchedMS2Count; m++)
    for (int n = 0; n < MatchedMS2[m]Mass_PointCount; n++)
    {
        MassPoint loopUsePoint = MatchedMS2[m]Mass_Point_FromFile[n];
        if (remassContains(loopUsePointMass) || loopUsePointMass == 00)
            continue;
        else
            remassAdd(loopUsePointMass);
        MassPoint theo_loopUsePoint = MatchedMS2[m]Mass_Point[n];
        List<int> clustermassindex = new List<int>();
        List<int> clusterindex = new List<int>();
        clusterindexAdd(m);
        clustermassindexAdd(n);
        for (int l = m - 10 > 0 ? m - 10 : 0; l < MatchedMS2Count; l++)
        {
            bool removesame = false;
            for (int k2 = 0; k2 < clusterindexCount; k2++)
            {
                if (!indexofMathcedsameContains(l) && clusterindex[k2] != l && MatchedMS2[l]Z == MatchedMS2[clusterindex[k2]]Z
                    &&            MatchedMS2[l]Mass_Point_FromFile[MatchedMS2[l]MS2MaxIntensityIndex]Mass            ==
MatchedMS2[clusterindex[k2]]Mass_Point_FromFile[MatchedMS2[clusterindex[k2]]MS2MaxIntensityIndex]Mass)
                {
                    templAdd(clusterindex[k2]);
                    matched_sameAdd(MatchedMS2[l]);
                    indexofMathcedsameAdd(l);
```

```
ModResModel mrm = MatchedMS2[l];
for (int iims = 0; iims < mrmMass_Point_FromFileCount; iims++)
{
    double iimass = mrmMass_Point_FromFile[iims]Mass;
    if (iimass != 00)
    {
        List<string> lstemp = overlapIEs[iimass];
        if (mrmIDContains("("))
        {
            string[] strgroup = mrmIDSplit(new char[] { '(', ')' })ToArray();
            lstempRemove(strgroup[0] + "-" + mrmZ + "+");
            string pre = strgroup[0]Split(new char[] { '-' })[0];
            if (strgroupContains("H2O+NH3"))
            {
                lstempRemove(pre + "-H2O-NH3-" + mrmZ + "+");
            }
            if (strgroupContains("2NH3"))
            {
                lstempRemove(pre + "-2NH3-" + mrmZ + "+");
            }
            if (strgroupContains("NH3"))
            {
                lstempRemove(pre + "-NH3-" + mrmZ + "+");
            }
        }
        else
            lstempRemove(mrmID + "-" + mrmZ + "+");
    }
```

```
                }
                removesame = true;
                break;
            }
        }
        if (!removesame)
        {
            for (int k = 0; k < MatchedMS2[l]Mass_Point_FromFileCount; k++)
            {
                if (MatchedMS2[l]Mass_Point_FromFile[k]Mass == loopUsePointMass && loopUsePointMass != 00)
                {
                    if (!clusterindexContains(l))
                    {
                        clusterindexAdd(l);
                        clustermassindexAdd(k);
                        break;
                    }
                }
            }
        }
    }
    if (clustermassindexCount == 1)
    {
        int currentIndex = clusterindex[0];
        int currentMassindex = clustermassindex[0];
        double MaxMass = MatchedMS2[currentIndex]Mass_Point_FromFile[currentMassindex]Mass;
        double intensity = InterpretedIPs[MaxMass];
        MatchedMS2[currentIndex]Mass_Point_FromFile[currentMassindex]Intensity = intensity;
```

```
        ModResModel mrm = MatchedMS2[currentIndex];
        List<string> lstemp = overlapIEs[MaxMass];
        if (mrmIDContains("("))
        {
            string[] strgroup = mrmIDSplit(new char[] { '(', ')' })ToArray();
            lstempRemove(strgroup[0] + "-" + mrmZ + "+");
            string pre = strgroup[0]Split(new char[] { '-' })[0];
            if (strgroupContains("H2O+NH3"))
            {
                lstempRemove(pre + "-H2O-NH3-" + mrmZ + "+");
            }
            if (strgroupContains("2NH3"))
            {
                lstempRemove(pre + "-2NH3-" + mrmZ + "+");
            }
            if (strgroupContains("NH3"))
            {
                lstempRemove(pre + "-NH3-" + mrmZ + "+");
            }
        }
        else
            lstempRemove(mrmID + "-" + mrmZ + "+");
        InterpretedIPs[MaxMass] = 0;
    }
    else
    {
        dividepeaks(lsAllOver, MatchedMS2, clusterindex, clustermassindex, overlapIEs, InterpretedIPs);
    }
```

```
                }
            MatchedMS2 = MatchedMS2OrderBy(ms => msMass_Point[0]Mass)ToList();
        }
        #endregion
        foreach (ModResModel mrm in MatchedMS2)
        {
            if (!indexofMathcedsameContains(MatchedMS2IndexOf(mrm)))
                if (!getComparePercentage(mrmMass_Point_FromFile, mrmMS2MaxIntensityIndex, mrmMass_Point, testParameterIPACO) &&
mrmNoMissing)
                {
                    if (nextlevel != null)
                    {
                        if ((mrmIDFirst() == 'y' || mrmIDFirst() == 'b' || mrmIDFirst() == 'c' || mrmIDFirst() == 'z' || mrmIDFirst() == 'x')
                            && !mrmIDContains("H2O") && !mrmIDContains("NH3") && !mrmIDContains("-a") && !mrmIDContains("-b")
&& !mrmIDContains("-H-") && !mrmIDContains("+H-"))
                        {
                            for (int i = 0; i < mrmMass_Point_FromFileCount; i++)
                            {
                                if (mrmMass_Point_FromFile[i]Mass != 0)
                                {
                                    mrmMass_Point_FromFile[i]IPMDOM_R = 00;
                                    mrmMass_Point_FromFile[i]IPADOM_R = 00;
                                    InterpretedIPs[mrmMass_Point_FromFile[i]Mass] += mrmMass_Point_FromFile[i]Intensity;
                                    mrmMass_Point_FromFile[i]Intensity = InterpretedIPs[mrmMass_Point_FromFile[i]Mass];
                                }
                            }
                            nextlevelAdd(mrm);
                            if (templContains(MatchedMS2IndexOf(mrm)))
```

```
                                    {
                                        nextlevelAdd(MatchedMS2[indexofMathcedsame[templIndexOf(MatchedMS2IndexOf(mrm))]]);
                                    }
                                }
                            }
                        }
            };
            for (int i = 0; i < templCount; i++)
            {
                for (int j = 0; j < MatchedMS2[indexofMathcedsame[i]]Mass_Point_FromFileCount && j < MatchedMS2[templ[i]]Mass_Point_FromFileCount;
j++)
                {
                    if (MatchedMS2[indexofMathcedsame[i]]Mass_Point_FromFile[j]Mass == MatchedMS2[templ[i]]Mass_Point_FromFile[j]Mass)
                        MatchedMS2[indexofMathcedsame[i]]Mass_Point_FromFile[j]Intensity = MatchedMS2[templ[i]]Mass_Point_FromFile[j]Intensity;
                }
            }
            return MatchedMS2;
        }
        public void dividepeaks(List<ModResModel> lsAllOver, List<ModResModel> MatchedMS2, List<int> clusterindex, List<int> clustermassindex,
Dictionary<double, List<string>> overlapIEs, Dictionary<double, double> InterpretedIPs)
        {
            double MaxMass = MatchedMS2[clusterindex[0]]Mass_Point_FromFile[clustermassindex[0]]Mass;
            double intensity = InterpretedIPs[MaxMass];
            if (intensity == 00)
            {
                for (int ii = 0; ii < clusterindexCount; ii++)
                {
                    int currentIndex = clusterindex[ii];
```

```
                int currentMassindex = clustermassindex[ii];
                MatchedMS2[currentIndex]Mass_Point_FromFile[currentMassindex]Intensity = intensity;
                MatchedMS2[currentIndex]Mass_Point_FromFile[currentMassindex]IPADOM_R = -5;
            }
        }
        else
        {
            List<int> temp_clusterindex = new List<int>();
            List<int> temp_clustermassindex = new List<int>();
            for (int ii = 0; ii < clusterindexCount; ii++)
            {
                int currentIndex = clusterindex[ii];
                int currentMassindex = clustermassindex[ii];
                temp_clusterindexAdd(currentIndex);
                temp_clustermassindexAdd(currentMassindex);
            }
            int x = clusterindexCount;
            int havecount = 0;
            bool isDeoverlapOver = false;
            for (int i = 0; i < temp_clusterindexCount(); i++)
            {
                int temp_n = temp_clusterindex[i]Count();
                double[] temp_a = new double[temp_n];
                for (int k = 0; k < temp_n; k++)
                {
                    int currentIndex = temp_clusterindex[i][k];
                    int currentMassindex = temp_clustermassindex[i][k];
                    ModResModel mrm = MatchedMS2[currentIndex];
```

```
        List<string> lstemp = overlapIEs[MaxMass];
        if (mrmIDContains("("))
        {
            string[] strgroup = mrmIDSplit(new char[] { '(', ')' })ToArray();
            lstempRemove(strgroup[0] + "-" + mrmZ + "+");
            string pre = strgroup[0]Split(new char[] { '-' })[0];
            if (strgroupContains("H2O+NH3"))
            {
                lstempRemove(pre + "-H2O-NH3-" + mrmZ + "+");
            }
            if (strgroupContains("2NH3"))
            {
                lstempRemove(pre + "-2NH3-" + mrmZ + "+");
            }
            if (strgroupContains("NH3"))
            {
                lstempRemove(pre + "-NH3-" + mrmZ + "+");
            }
        }
        else
            lstempRemove(mrmID + "-" + mrmZ + "+");
    }
    if (!isDeoverlapOver)
    {
        for (int k = 0; k < temp_n; k++)
        {
            int currentIndex = temp_clusterindex[i][k];
            int currentMassindex = temp_clustermassindex[i][k];
```

```
                            if (!IsAllOverContains(MatchedMS2[currentIndex]))
                            {
                                    temp_a[k] = MatchedMS2[currentIndex]Mass_Point[currentMassindex]Intensity *
                                            MatchedMS2[currentIndex]Mass_Point_FromFile[MatchedMS2[currentIndex]MS2BasePeakIndex]Intensity
                                               / MatchedMS2[currentIndex]Mass_Point[MatchedMS2[currentIndex]MS2BasePeakIndex]Intensity;
                            }
                            else
                            {
                                    temp_a[k] = MatchedMS2[currentIndex]Mass_Point[currentMassindex]Intensity *

MatchedMS2[currentIndex]Mass_Point_FromFile[MatchedMS2[currentIndex]MS2MaxIntensityIndex]IntensityPercentage
                                               / MatchedMS2[currentIndex]Mass_Point[MatchedMS2[currentIndex]MS2MaxIntensityIndex]Intensity;
                            }
                            havecount++;
                    }
                    double temp_aSum = temp_aSum();
                    if (temp_aSum == 00)
                    {
                            for (int k = 0; k < temp_n; k++)
                            {
                                    int currentIndex = temp_clusterindex[i][k];
                                    int currentMassindex = temp_clustermassindex[i][k];
                                    temp_a[k] = MatchedMS2[currentIndex]Mass_Point[currentMassindex]Intensity;
                                    temp_aSum += temp_a[k];
                            }
                    }
                    for (int k = 0; k < temp_n; k++)
                    {
```

```
                    int currentIndex = temp_clusterindex[i][k];
                    int currentMassindex = temp_clustermassindex[i][k];
                    {
                        temp_a[k] = temp_a[k] / temp_aSum;
                        MatchedMS2[currentIndex]Mass_Point_FromFile[currentMassindex]Intensity = temp_a[k] * intensity;
                        MatchedMS2[currentIndex]Mass_Point_FromFile[currentMassindex]IPADOM_R = -5;
                    }
                }
                isDeoverlapOver = true;
                InterpretedIPs[MaxMass] = 00;
            }
        }
    }
  }
 }
}
```
